# Syntax Acquisition in Healthy Adults and Post-Stroke Individuals: The Intriguing Role of Grammatical Preference, Statistical Learning, and Education

**DOI:** 10.3390/brainsci12050616

**Published:** 2022-05-09

**Authors:** Simon Kirsch, Carolin Elser, Elena Barbieri, Dorothee Kümmerer, Cornelius Weiller, Mariacristina Musso

**Affiliations:** 1 Department of Neurology, University Medical Center Freiburg, Breisacherstrasse 64, 79106 Freiburg, Germany; simon.kirsch@uniklinik-freiburg.de (S.K.); carolin.elser@elser-web.de (C.E.); dorothee.kuemmerer@ib.de (D.K.); conelius.weiller@uniklinik-freiburg.de (C.W.); 2 Clinic for Psychiatry and Psychotherapy, Hauptstraße 8, 79104 Freiburg, Germany; 3 Department of Communication Sciences and Disorders, Northwestern University, 2240 Campus Drive, Evanston, IL 60208-2952, USA; elena.barbieri@northwestern.edu; 4 Medizinische Akademie, Schule für Logopädie, Schönauer Str. 4, 79115 Freiburg, Germany

**Keywords:** syntactic predictors, aphasia, education

## Abstract

Previous work has provided contrasting evidence on syntax acquisition. Syntax-internal factors, i.e., instinctive knowledge of the universals of grammar (UG) for finite-state grammar (FSG) and phrase-structure grammar (PSG) but also syntax-external factors such as language competence, working memory (WM) and demographic factors may affect syntax acquisition. This study employed an artificial grammar paradigm to identify which factors predicted syntax acquisition. Thirty-seven healthy individuals and forty-nine left-hemispheric stroke patients (fourteen with aphasia) read syllable sequences adhering to or violating FSG and PSG. They performed preference classifications followed by grammatical classifications (after training). Results showed the best classification accuracy for sequences adhering to UG, with performance predicted by syntactic competence and spatial WM. Classification of ungrammatical sequences improved after training and was predicted by verbal WM. Although accuracy on FSG was better than on PSG, generalization was fully possible only for PSG. Education was the best predictor of syntax acquisition, while aphasia and lesion volume were not predictors. This study shows a clear preference for UG, which is influenced by spatial and linguistic knowledge, but not by the presence of aphasia. Verbal WM supported the identification of rule violations. Moreover, the acquisition of FSG and PSG was related to partially different mechanisms, but both depended on education.

## 1. Introduction

Numerous findings [1,2,3,4,5] consider language acquisition an instinct, i.e., it can be viewed as a form of ‘innately guided learning’, where universal grammar (UG) instructs the learner ‘which cues it should attend to’ ([6], p. 85). UG is the common syntactic design underlying the “Babel’s of languages” [2,3,7]. According to this view, the ability to abstract and use rules adhering to UG principles is necessary to acquire a language. Musso et al. (2003) demonstrated that acquiring linguistic competence in a foreign language in adulthood involves the same brain systems that support language acquisition in the native language, when the new language (regardless of the language family it belongs to) is based on UG principles. Learning without a priori restrictions on the learning space (e.g., when the relationship among amodal variables is governed by abstract mathematical-like rules) is possible in healthy adults, but not in a language savant [8]. Moreover, Musso et al. (2003) showed that the acquisition of UG rules showed a different anatomical framework than learning abstract mathematical-like rules [7]. The conformity of a language to UG principles is also labelled *grammaticality*. According to generative linguistics, the recognition of grammaticality in linguistic stimuli—sentences, but also syllables—is intuitive. Yang ([9] p. 453) summarizes “Schematically, learning goes as follows (see [10] for mathematical details): For an input sentence, the child: (i) with probability Pi selects a grammar Gi, (ii) analyzes s with Gi, (iii) if successful, reward Gi by increasing Pi, otherwise punish Gi by decreasing Pi”. The main question here is if structural knowledge attribution solely reflects language experience, or if it also reflects other metacognitive experiences of knowledge [11]. Another crucial principle for language acquisition is the *depth of embedding* that defines *the grammar type* (GT) [12,13]. In “Chomsky hierarchy”, finite state grammar (FSG) or regular grammar is the easiest form of GT. It is used even by non-human animals [14,15,16] and is characterized by adjacent dependencies, e.g., a sequence such as A1B1A2B2. In contrast, phrase structure grammar (PSG) or supra-regular grammar is defined by non-adjacent dependencies or recursive embeddings, e.g., sequences such as A1A2B2B1, where the [AB] part is embedded, and is generally considered uniquely human [14,15,16]. Despite the importance of such syntax-internal principles for language processing and acquisition, even the so-called Minimalist Program of research in linguistics [12] recognizes that the faculty of language in the narrow sense (FLN)—“the abstract computational system alone” ([14] p. 1571)—interacts with *the faculty of language in the broad sense (FLB).* The FLB consists of the sensorimotor systems (language perception and production) and relates to syntax-external factors including ecological, cultural, and social environments, as well as conceptual–intentional computations—mainly working memory—in order to generate novel linguistic utterances [14]. 

These questions outlined above inspired this behavioral study, which aimed to identify the syntax-internal and -external factors predicting the ability to learn and generalize novel, regular and supra-regular syntactic rules within meaningless linguistic stimuli in healthy adults and post-stroke individuals with and without aphasia. According to Chomsky ([17] p. 3), “linguistic theory is concerned with an ideal speaker-listener in a completely homogeneous speech community, who knows its language perfectly and is unaffected by such grammatically irrelevant conditions as memory limitations”. Therefore, as an “ideal” group of speaker–listeners, we recruited 37 young, healthy adults with higher education, excellent knowledge of their native language (German), a homogeneous sociolinguistic community, and irrelevant memory limitation (see Method) to investigate L2 grammar acquisition in near to “ideal” conditions. This study also tested 49 left-hemispheric stroke patients (LHSP). They can be considered “non-ideal speaker-listeners”, as they suffered a stroke affecting language-related brain regions, which caused impairments within the faculty of language and memory competence [18]. This group of individuals may offer unique insights into the following questions:

### 1.1. First, Is the Depth of Embedding an Overarching Principle Relevant for Syntax Acquisition?

LHSP may present syntactic deficits, such as the tendency to avoid complex sentences in production, as well as difficulty understanding complex sentences in comprehension. Few studies have reported increasing syntactic deficits in LHSP as the number of phrasal nodes in the syntactic tree increases [19,20,21]. Most data show difficulties in processing syntactic movement: individuals with Broca’s aphasia show difficulties in comprehension and production of sentences involving movement of a noun phrase (NP-movement [13]) from an argument position to another argument position such as passive sentences [19,20,22]. Some studies also indicate that individuals with Broca’s aphasia or anomic aphasia present deficits in processing sentences with movements of an argument to a non-argument position (Wh-movement) such as in object relative (OR) clauses [19,21]. The relevance of syntactic movement to syntactic processing is also highlighted by treatment studies, which indicate that training sentences with Wh-movement (e.g., OR structures) does not affect production or comprehension of sentences with NP-movement, although generalization across sentences with similar movement operations is commonly seen (e.g., from OR structures to object Wh-questions) [23,24]. However, syntactic deficits differ even among patients with lesions at similar locations [22,25]. These findings are in favor of growing evidence that Chomsky’s hierarchy is not directly relevant for neurobiological systems [26,27]. To the best of our knowledge, there are no reports that clearly tested FSG and PSG in LHSP. 

The present study aimed to evaluate Chomsky’s hierarchy hypothesis by testing FSG and PSG acquisition in LHSP, with the prediction that, should the depth of embedding be an overarching principle relevant for syntax acquisition, learning PSG will be more impaired than learning FSG.

### 1.2. Second, to What Extent Is Syntax Acquisition Affected by the Presence of Aphasia?

Aphasia—an acquired impairment in understanding or producing language—frequently occurs after a left-hemispheric ischemic stroke. Studies in patients with mild to residual aphasia have shown that grammatical deficits may occur even in the presence of minimal language impairment [20,28]. This finding supports the relevance of syntax for the language domain [13,14]. However, patients with aphasia and grammatical deficits often show impaired processing of music syntax [29], suggesting that the claim that the combinatorial system of syntax is unique to language should be abandoned [14,30]. Rather, speech seems to be a privileged input for rule learning. For example, learning to structurally combine syllables, which are the basic unit of speech [31], is possible in babies, even though the FLB is very limited [32,33], or in syntactically impaired patients with global aphasia [34]. It has been postulated that severely impaired lexical comprehension may mask residual syntactic processing ability and, therefore, tasks involving natural language forms are unable to detect this retained capacity. This evidence, however, is limited to the acquisition of rules with low syntactic complexity. Thus, it remains unclear if the acquisition of syntax in its complexity requires FLB. The Aachen Aphasia Test (AAT) [35,36], the standard diagnostic battery for chronic aphasia in Germany, allows to evaluate and quantify language competence in several domains such as repetition, naming, comprehension, reading, and writing modalities, but not syntax [35]. Therefore, performance on this test can be considered a proxy of FLB’s competence. This study aimed to investigate the extent to which the ability to learn and generalize novel FSG and PSG is predicted by general language competence (as reflected by the AAT).

### 1.3. Third, Is Syntax Acquisition Affected by Working Memory Deficits?

It is well-established that—when learning a novel language—adults often relies on the conscious remembering of previously learned information, for example during explicit grammar-teaching of language-specific word order as in Musso et al. (2003). Explicit learning is very effective mainly when features of the novel (L2) grammar are diametrically opposed to (or radically different from) the manner of expression in the student’s L1. Studies in LHSP significantly contribute to this topic as they show that syntax acquisition in adults can be learned though implicit learning (in a similar way as children), as it has been shown that LHSP with agrammatism are able to learn under implicit, but not explicit, conditions [37]. The question is whether they can extract and use structural patterns from novel complex stimuli because of the intact ability to use implicit procedural learning strategies or the preserved intuitive ability to identify UG-based structural knowledge.

Artificial grammar learning (AGL) studies [38,39] have highlighted the relevance of both rule knowledge, which relies on prior experience, and procedural learning for syntax acquisition. Implicit, probabilistic learning mechanisms identify perceptual and/or structural similarities across test stimuli [40,41]. Such knowledge goes from specific to general [42,43] and is stimulus-specific, i.e., it is closely tied to the perceptual characteristics of the input and is dependent on working memory [41]. Thus, in this view, learning and generalizing beyond the input is driven by properties of the learner that are not exclusively linguistic in nature. However, patients with Parkinson’s disease, who generally have difficulty with rule-based processing [44], showed intact AGL ability when using letter strings [45]. A proposed explanation of this finding was that verbal WM, but not spatial WM, is relevant for acquiring novel linguistic competence [46]. This hypothesis is supported by studies in LHSP with Broca’s aphasia, an aphasia syndrome mostly associated with agrammatism, documenting an association between verbal working memory deficits and difficulties with implicit learning of novel phoneme (but not non-linguistic) sequences [47]. Unfortunately, this study is not sufficient to confirm the necessity of verbal WM competence for syntax acquisition, due to the small number of participants and the use of different stimuli in verbal and visual modalities, which could have biased the results. Moreover, there is evidence first that not only verbal WM but also spatial WM may be related to syntax processing [48]. Second, spatial WM, as measured by spatial span, can be significantly disrupted in a subset of individuals with LHSP [49]. Therefore, our experimental design controlled verbal and spatial WM while testing the ability to learn and generalize novel FSG and PSG in LHSP.

Most importantly, it remains unclear when humans in ideal conditions use the memory-driven learning approach. On the one hand, it has been suggested that human cognition relies on stimulus-specific representations for some tasks, and on abstract learning for others [21]. Namely, memory-driven learning is predominantly used when concrete knowledge cannot be relied upon (for example, in a transfer of learning task). Alternatively, memory may be relevant in tasks requiring monitoring of lexical selections [7,50]. As such, working memory resources were discussed to underlie, even in ideal conditions, the relationship between the processing cost of integrating arguments in the sentence and the distance between fillers and gaps (i.e., the longer the distance, the greater the processing cost) [51]. Based on these premises, we predict that reliance on memory resources will be greater for PSG than for FSG, and that a significant relationship between WM competence and syntax acquisition accuracy will be found in both HP and LHSP.

### 1.4. Fourth, Do Stroke-Induced Brain Damage, Brain Reserve and Brain Cognition Play a Role in Syntax Acquisition in Stroke Patients?

A growing number of studies have shown that such variables can predict language ability after stroke [52,53,54,55,56]. For example, Ditges et al. [20] recently showed that the National Institute for Health Stroke Scale (NIHSS), a score reflecting stroke severity [57], significantly predicted sentence production difficulties in patients with aphasia. In LHSP, education, which is generally considered a proxy for cognitive reserve, and age, as a proxy for brain reserve, predicted motor output [58], but not language recovery [59]. Ditges et al. [20] found that age but not education was a predictor of syntactic processing in patients with aphasia. However, patients with agrammatism could improve after therapy, which can be considered a form of education, confirming that linguistic expertise is learnable. The role of education on syntax is more intriguing than that of other demographic variables. Some authors assert that linguistic expertise is a cultural tool [60]. Other authors argue that formal education affects the ability to use syntactic rules (linguistic performance), while grammatical knowledge (syntactic competence) depends on genetic and universal mechanisms in humans [14,61,62]. We tested the role of both age and education on FSG and PSG learning as a way to evaluate their relevance for syntax acquisition.

This study tested syntax acquisition in 37 healthy adults and 49 LHSP, 20 of which had an aphasia at the time of stroke and 14 at the time of testing, using AGL and AG-generalization (AGG) paradigms. Participants learned to identify and recognize structural relations expressed in the sequence solely based on examples and feedback provided during training sessions. They initially performed preference classifications and, after training, grammaticality judgments. After three training sessions, participants were asked to perform grammaticality judgements on novel sets of syllable sequences adhering to or violating the same rules, thereby requiring generalization of the learned rule to an unknown linguistic environment. Since some authors’ comparative studies have shown that non-human primates can learn some novel (basic) syntactic rules but cannot apply them to a novel context [16]—A finding that points to generalization as a remarkable ability of humans [14]—we analyzed both AG learning (AGL) and generalization (AGG) separately.

To evaluate the influence of syntax-external variables on AGL and AGG, a verbal WM condition was introduced, and the effects of demographic factors (e.g., age, gender, education), stroke severity-related factors (e.g., lesion size and NIHSS score), spatial working memory (e.g., Corsi test [63]), and general language (using the standard language AAT [35] and the Token Test (TT)) [64] and syntactic competence (as reflected by the German tests “komplexe Sätze” [65] and “Sätze verstehen” [66]) were assessed by statistical analyses.

## 2. Materials and Methods

### 2.1. Study Design

The study protocol adhered to strict inclusion and exclusion criteria and provided a collection of detailed demographic and clinical data using assessments suggested by international clinical guidelines for stroke [67].

All participants (healthy adults and stroke patients) were included in the study if they were: (1) native speakers of German, (2) right-handed [68], (3) able to understand task instructions and provide informed consent. Exclusion criteria for all participants were: (1) history or current diagnoses of other medical, neurological, or psychiatric disorders, (2) severe hearing or vision deficits, (3) early bilingualism [69], (4) music education at the professional level [31], (5) contraindications for MRI. Inclusion and exclusion criteria were specifically adapted for testing “ideal” and “non-ideal speaker-listener”. Thus, participants in the “ideal” group met the following criteria: (1) they were attending or had graduated from medical school, (2) they had never received speech therapy, (3) they did not have a history of language or cognitive deficits. Stroke participants were included only if they suffered from a first ever ischemic lesion of the left-middle cerebral artery [70]. Patients with bilateral or hemorrhagic strokes were excluded [54]. All stroke participants underwent a structural MRI, which allows the most precise mapping of stroke lesions, on a 3T Trio scanner (Siemens) on an average of 1.231 days after symptom onset (SD 2.1, range 0–9 days).

Participants’ age, gender and education as well as, for LHSP, data reflecting stroke severity using the NIHSS [57] and brain damage using the lesion volume (see Appendix A for detailed information about its calculation) were obtained from the institutional research database of the clinic Freiburg (Large Scale Project), from which all patients of this study were selected. Demographic and clinical data were entered as variables in statistical analyses.

The presence of aphasia in LHSP was determined through the analysis of spontaneous speech [71] and performance on the TT, a standardized and highly sensitive aphasia test [64] both in the acute phase after stroke and at the time of AG testing. In patients with pathological *TT* and spontaneous speech, the characterization of the language profile and the classification into the classic forms of aphasia were established using the well standardized [35] and highly reliable [72] AAT. The factor “aphasia” in the acute phase after stroke reflected the effects of the lesion of a language-relevant brain network. The factor “aphasia” at the time of AG testing reflected performance on the *AAT*, which encompassed language comprehension, reading, writing, repetition, and naming. Given that syntax is not assessed by the AAT, performance on this task was considered a proxy of FBL. At the time of AG testing, “Komplexe Sätze” [65] and “Sätze verstehen” [66] were used to test syntactic deficits. These two tests investigate the ability to produce and comprehend, respectively, non-canonical clauses (i.e., passive, whom questions and object relative clauses).

Verbal WM was tested in one of the experimental conditions (see Method), while spatial short-term working memory was assessed with the Corsi blocks tasks [63].

The study was approved by the local ethics committee (EK281/13, EK156/14) and was conducted in accordance with the Declaration of Helsinki of the World Medical Association. Full written consent was obtained from all patients or their legal guardians before participation.

### 2.2. Participants

#### 2.2.1. The Ideal Learner Group

Thirty-seven healthy participants (HP) (20 males, 17 females, mean age = 25.77 ± 5.79 years) with higher education (ed. age: 18.18 ± 3.18, range: 13–23) and similar socioeconomic context (26 were students at, and 11 had graduated from, medical school) participated in the experiment. According to the inclusion criteria, HP were unaffected by spatial WM (ceiling accuracy on Corsi forward and backward) and had ceiling accuracy in the two German syntax tests.

#### 2.2.2. Left-Hemispheric Stroke Patients

**Figure 1:**
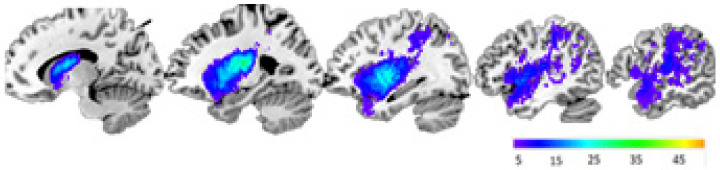
LHSP brain lesion: Overlap of the binarized lesions of the left-hemispheric stroke patients included in the study (*n* = 49) in radiological convention mapped onto the Montreal Neurological Institute (MNI) template. Overlays are thresholded to show lesions present in at least 5 individuals as included in the lesion–symptom mapping analysis. The color bar indicates the degree of overlap of lesions.

Seventy-two LHSP, who were part of the Freiburg University Hospital’s Large-Scale Project, were screened for this study after their stroke (on average, at 2.05 years, range: 5.6 months–7 years) during 2019. Of these participants, two refused to participate in the experiment, two had a new stroke, and 19 dropped out of the study before the AGL experiment was completed. The remaining 49 LHSP—14 female, mean age = 64.55 ± 13.10 (range: 32–85), mean education = 13.33 ± 3.65 (range: 8–23)—completed both grammatical conditions of the AGL experiment (see Method). Group lesion maps indicated that lesions comprised the entire territory of the middle cerebral artery, with maximum lesion overlap in the basal ganglia and insula/peri-insular white matter (Figure 1).

The mean NIHSS at testing time was 1.22 ± 2.02 (range: 0–10). Corsi forward performance was 5.32 ± 0.85 which, according to [63], is still in the normal range, and Corsi backward was 4.92 ± 1.19. TT performance was, according to Cohen (66), in the normal range on average (48.2 points), although there was a large standard deviation (5.99, range 13–50). According to TT and AAT, 15 LHSP never had aphasia, 20 initially had aphasia (severity: 2.5 ± 1.40) but completely recovered over time, and 14 had a well-documented history of aphasia, with stable and predominantly mild language impairment at the time of testing (AAT mean severity = 1.14 ± 0.92, AAT mean global score = 454.77 ± 100.11, range 201–543). Of these, 3 presented with residual, 8 with amnestic, 2 with Broca’s and 1 with Wernicke’s, aphasia. Results of the two tests examining syntactic abilities in German were available only for 33 LHSP, 8 of whom also presented with aphasia on the AAT at the time of testing. For the other 15 LHSP, testing time was too long, and they dropped out before completing the syntactic tests. In line with previous findings [20,23,73], syntax was impaired even in patients without aphasia diagnosis as defined by the AAT. Table 1 shows that the three groups—the aphasia group, no aphasia group, and recovery group—significantly differed in TT, lesion size and Corsi f. In the syntax tests, patients showed most variance in the ability to produce object relative clauses.

### 2.3. The Experimental Design

This study employed an AGL paradigm. An AGL paradigm may be particularly well suited to explore sequential processing ability in the face of aphasia as it allows us to bypass the interference of semantic, pragmatic and morphosyntactic deficits [34]. Moreover, due to potential difficulties in declarative learning in LHSP, a learning approach based on explicit strategies, such as in Musso et al. (2003) [7], is not ideal. Other advantages of the AGL paradigm are that, in contrast to the use of natural language stimuli, artificial grammar allows testing of the effect of prior exposure to linguistic stimuli.

**Figure 2:**
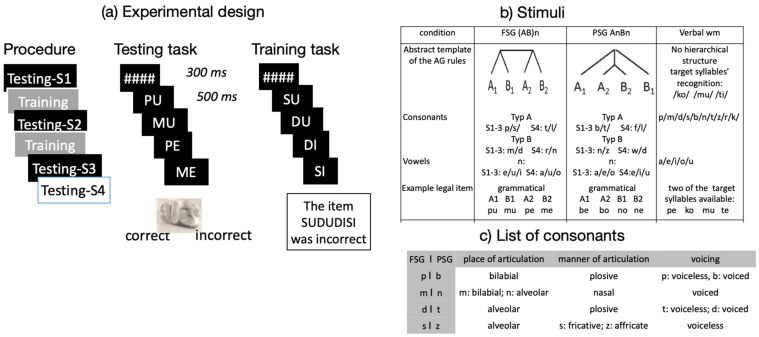
Experimental design and general structure of the stimuli. (**a**) The experiment consisted of three—Finite State Grammar (FSG), Phrase Structure Grammar (PSG) and verbal working-memory (verbal WM)—condition-blocks. Each condition-block included testing sessions and training sessions using the same lexicon (but different items). The fourth testing session of the Artificial Grammar (AG) conditions contains a novel lexicon. In each session, participants’ were asked to press a button on a mouse for correct and another button for incorrect items. (**b**) Grammatical stimuli consisted of syllable sequences observing adherence to a syntactic rule, while in the ungrammatical stimuli, the hierarchical structure was violated by the exchange of two adjacent (in scrambling) or non-adjacent (permutation) elements (vowels or consonants). In the verbal WM condition, the order of the syllables was not relevant, and no abstract template was identifiable. A sequence was defined as correct when it presented at least two of the three target syllables. (**c**) List of consonants used in the AG conditions.

The experiment consisted of three condition-blocks: two testing AG acquisition (one within the framework of FSG and one within that of PSG), and one testing verbal WM (control condition, see Figure 2). The AG condition-blocks contained four testing sessions and two training sessions—between testing session 1 and 2 as well as between 2 and 3 (order: testing session (S)1–training–testing S2–training–testing S3–testing S4). The first testing session reflected the pre-training performance, the third session showed the post-training performance, while in the fourth session, the lexicon was changed so that it reflected the ability to generalize novel syntactic competence. Therefore, we distinguished in the two AG experimental conditions: Artificial Grammar Learning (AGL) and Artificial Grammar Generalization (AGG). The verbal WM condition included only three testing sessions.

Each testing session included 21 items (10/11 correct in a session, 11/10 correct in another session), and each training session included 12 items (6 correct).

The three condition-blocks (FSG, PSG, verbal WM) were presented in a pseudorandomized manner to prevent order effects. In this process, FSG was never directly followed by PSG or vice versa because of the similarity of the rule.

### 2.4. Stimuli

All stimuli consisted of a sequence of four syllables, i.e., consonant–vowel pairs (Figure 2). The order of syllables in a sequence differed between conditions and response type (correct and incorrect). In the two AG conditions, consonants were either alveolar or labial, and were assigned to two classes (A and B) [74,75]. Different syllables were used in the FSG and in the PSG conditions, as well as in testing sessions 1–3 and the fourth session. Training and testing sessions used the same consonants and vowels but different syllable sequences (i.e., different stimuli using the same syllables) [75]. In the fourth session, different syllables belonging to the same classes were employed [76]. Syllables and vowels used in the verbal WM are listed in Figure 2.

- Correct stimuli:

In the FSG condition, grammatical sequences were generated by FSG; therefore, the syllables’ order in correct sequences followed the abstract template A1B1A2B2.

In the PSG condition, grammatical stimuli were generated by PSG, i.e., correct sequences followed the abstract template A1A2B2B1.

In the verbal WM condition, sequences were not generated based on a grammar; thus, there was not a specific structural relationship between the four consonants and vowels. Syllable sequences were considered correct when at least two of the three given target stimuli—“ko”, “mu”, and “ti”—were present.

- Incorrect stimuli

In the two AG conditions, ungrammatical sequences were generated by violating the hierarchical dependency of the elements (consonants or vowels [75]). This was achieved 50% of times via scrambling (i.e., exchanging adjacent elements within a sentence) and 50% of times via permutation (i.e., exchanging non-adjacent elements within a sentence). Violations affected consonants (scrambling in SFG Pu Mu Me Pe and permutation in PSG, Be No Bo Ne) or vowels (permutation in FSG as Pu Me Pe Mu, and scrambling in PSG Be Bo Ne No). Scrambling violations were recognizable on the third syllable, while permutation violations wer already recognized by the second syllable. Notably, counting the number of stimuli of class A and B and monitoring the repetition of vowels [75] were not possible strategies for the identification of ungrammatical sequences. This is because the numbers of An and Bn match in ungrammatical items.

In the verbal WM condition, incorrect stimuli consisted of sequences of four diverse syllables which did not contain two of the target syllables.

### 2.5. Procedure

Participants were required to read the syllable sequence and press the right-side button on a mouse for correct stimuli, and the left-side button for incorrect stimuli. In the verbal WM condition-block, participants were explicitly instructed to remember the three target syllables and to consider a sequence as correct when it contained at least two of the given syllables. In the two AG condition-blocks, participants performed a preference classification in the pre-training sessions, i.e., they were asked to judge based on their preference or “gut feeling” [77]. After receiving training and feedback, through which they could extract the rule governing the dependency of consonants and vowels in a sequence, they performed a grammatical classification. Before starting the experiment, a short trial version of the paradigm using syllable sequences without a regular arrangement (as /la/ /lu//la/ /lu/) was presented in order to familiarize participants with the task.

The experiment started with a short-written explanation of the following block. Figure 2a illustrates the procedure. During the experiment, the syllable sequences were presented to the participants visually, with white text against a black background, on a PC display using Presentation^®^ Version 14.6 software (Neurobehavioural Systems Inc., Albany, CA, USA: www.neurobs.com, accessed on 12 April 2022). Each trial started with a 300 ms presentation of four hashtags (“####”) in the middle of the screen, followed by the four syllables presented one after another for 500 ms each. Participants had max. 4 s to provide their response. Both accuracy and reaction times were recorded.

During the training sessions, participants had to attentively read the stimuli, which were presented in the same way as in the testing sessions, and to judge their accuracy. However, their response was not required and, after 4 sec, a visually presented statement explained whether the actual syllable sequence was correct or not.

After the experiment, the participants were asked to fill out a questionnaire, in which they were asked about task strategies as well as suggestions and criticisms of the paradigm. Unfortunately, data derived from the administration of the questionnaire to the participants were not informative.

### 2.6. Data Analysis

#### 2.6.1. The Data Set

Separate analyses were run to evaluate AGL and AGG.

For AGL, the data set consisted of the data of 37 HP and 49 LHSP for each of the three condition-blocks. As successful learning was defined as a significant positive deviation of classification accuracy from chance level (50 percent) [78], the main analysis focused on the responses derived from the third session, i.e., the post-training session. Data from the pre-training sessions were checked to investigate preference classification and evidence of learning during the first sessions (trial effect). Data from both sessions were used to compute change in accuracy from the pre- to post-training phase, which reflects AG learning from training-induced feedback [79]. 

For the AGG analysis, changes in response accuracy between the third (post-training) and the fourth (generalization) sessions were analyzed. Notably, of the 49 LHSP, 24 dropped out before completing the study; thus, the final number of LHSP for the AGG analysis was 25 (7 female, age mean = ±(range:), educational age (range: 8–23). Of these, according to TT (96.33 ± 5.6), six LHSP had never had aphasia, 11 initially had aphasia but had fully recovered at time of testing, and eight had aphasia at time of testing.

#### 2.6.2. Mixed Effects Logistic Regression Analysis

Participants’ data were analyzed using mixed effects logistic regression models [80]. Data analyses were run in R version 4.0.3 [81] using the glmer function with the bobyqa-optimizer included in the “lme4” package [82]. *p*-values for these models were obtained via Satterthwaite’s degrees of freedom method using the “lmerTest” package [83] and effects were plotted using the “effects” package [84]. Marginal R-squared (variance explained by fixed effects) was calculated via the r.squaredGLMM function in the “MuMIn” package [85]. We used an alpha level of 0.05 for all statistical tests. In all models, accuracy was entered as the binary dependent variable (0 = incorrect, 1 = correct). Subject-related variability was controlled by the use of random effect intercepts [80].

- Basic Models

Based on a priori theoretical motivations explained in the introduction, the following syntax-internal factors were entered as independent variables: Grammaticality (grammatical vs. ungrammatical), Grammar Type (FSG vs. PSG), and their interaction. Thus, the basic mixed effects logistic regression model was Correct~Grammaticality × Grammar Type + (1|Participant).

Moreover, we tested if, during the pre-training sessions, there was a significant trial effect calculating the model Correct~Grammaticality/Grammar Type × trial-Number + (1|Participant).

Lastly, possible effects of error type during the pre- and post-training sessions were analyzed by calculating a model with ungrammatical items, grammar type and all the possible different coding of error type: (a) scrambling vs. permutation errors and (b) articulatory vs. phonological errors.

- Models including syntax-external factors

In HP, the following predictors were considered: the two syntax-internal variables—grammaticality and grammar type—and the syntax-external covariates—the performance on the verbal WM condition, age, educational age, and gender.

In LHSP, the syntactic external covariates were: (1) verbal working memory (i.e., accuracy on the best session of the verbal WM condition-block), (2) spatial working memory (i.e., performance on Corsi Forward Span), (3) general language competence defined by the TT and AAT, (4) severity of neurological symptoms, as reflected by the NIHSS score (calculated at time of testing, and as the score change between time of stroke and time of testing), (5) lesion size, (6) age, (7) education, (8) gender, and (9) grammatical competence in the native language, as indexed by accuracy in production of object relative clauses (ORCP).

Before analyzing these covariates with mixed effects logistic regression, we checked the data for normality using Q–Q plots and the Shapiro–Wilk test. Exploratory correlations between the external factors were assessed using the nonparametric partial Spearman test. Their collinearity was checked using the Variance Inflation Factor. Additional diagnostic checks were performed by examining residual plots (using the resid function inR) and the distributions of the random effects. Diagnostic checks are provided in the Supplementary data analysis.

After the assumptions for the binomial logistic model were tested and none were violated, subsequent mixed effects logistic regression models including syntax internal and external variables were calculated:

First, each covariate was entered into the basic model as a main effect and in interaction with the other dependent variables: Prob(Correct) × Grammaticality × Grammar Type × Covariate + (1|Participant).

Second, we calculated the best-fit model to identify—within the large set of possible predictors—the combination of variables with the best predictive power, while taking model complexity into account. Due to the high number of variables, we used a two-step approach. In the first step, we conducted an exhaustive search across models containing all possible combinations of predictors that were allowed by the collinearity analysis, without including any interaction terms, and identified the model with the lowest Akaike Information Criterion (AIC). In the second step, we built all possible interaction models from the set of variables within the first step in order to test whether allowing for specific interactions between variables increases model fit in terms of AIC. The best-fit model was calculated only for the data of Session Nr. 3, as models of generalization and pre–post training differences require covariates to interact with the Session Nr. variable in the first place, which is not compatible with our two-step approach. However, an exhaustive search for the best-fit model among all covariates including their possible interactions would lead to a combinatorial explosion, exceeding the computational power of our hardware.

Third, session number was entered in the first basic model: Prob(Correct) ~ Session Nr. × Grammar Type × Grammaticality + (1|Participant) to evaluate differences in the combined effect of grammar type and grammaticality between sessions, and in the model that included external variables—Prob(Correct)~Session Nr. × Grammar Type/Grammaticality × Covariate + (1|Participant)—to explore the effect of covariates on AG performance and session number.

To investigate the effect of aphasia in LHSP, participants were assigned to three different groups, based on their AAT and TT scores at two different points in time (see Table 1): (1) no aphasia group (*n* = 15), recovery group (*n* = 20) and chronic aphasia group (*n* = 14).

## 3. Results

Most participants were unable to explain why syllables were well- or ill-formed. 

Only the results of the best-fitting models and of all the model comparisons that were relevant to our hypotheses are reported in the manuscript. Results of the distribution, collinearity, model-fit checks, and descriptive statistics are reported in the Supplementary data analysis, and the results of all other mixed effects logistic regression analyses are reported in the Supplementary results.

For all results, we reported the regression estimates for capturing the impact of the explanatory variables, as well as the standard error of these regression estimates. R-squared values are reported as a goodness-of-fit measure. We additionally reported the standard deviation of random intercepts as an estimate of (unexplained) between-subject variability. Significance was indicated with * for *p* < 0.05, ** for *p* < 0.01, and *** for *p* < 0.0001.

### 3.1. Syntax Acquisition in Healthy Participants (Ideal Native Speaker–Listeners)

#### 3.1.1. Best-Fit Model for AG Learning

The best-fit model for AGL included the AG accuracy of all 37 HP, grammaticality, grammar type, the performance on the verbal WM condition, age, education, and gender. The model with the lowest AIC value was: Correct~Grammar Type × Grammaticality + (1|Participant). Table 2A shows that the only significant main effect was grammaticality: grammatical items were better identified than ungrammatical items. The two-way interaction of grammaticality × GT was also significant: within ungrammatical items, PSG items were less accurately classified than FSG items. The R-squared of the model was low (R^2^ = 0.081) because of participants’ low variance within post-training performance.

Accuracy on the verbal WM condition was high, but not at ceiling (mean 0.86 ± 0.128). Although verbal WM was not significant as a main effect in any of the analyses, a significant interaction was found between verbal WM, Grammaticality, and GT (see Table 2B). Plots in Figure 2c show that verbal WM was positively associated with the identification of ungrammatical PSG (but not FSG) items. After the introduction of verbal WM, the interaction of grammaticality × GT was no longer significant (Table 2B).

Note that age, educational age, and gender were not predictors for AGL, even in the single exploratory basic model (see Supplementary results).

#### 3.1.2. Sessions Effects in AGL

**Figure 3:**
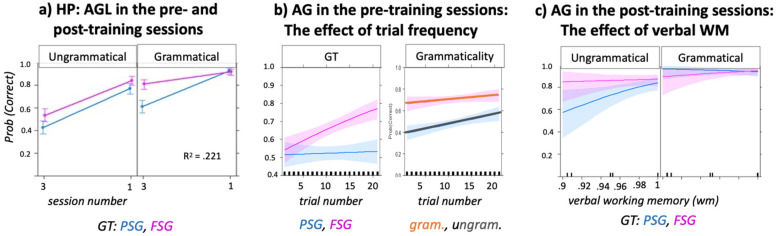
Artificial Grammar Learning (AGL) in ideal native speakers (*n* = 37). (**a**) The figure displays the results of the significant Session × Grammaticality × Grammar Type (GT) interaction in the group of healthy participants (HP). For grammatical trials (gram), but not ungrammatical trials (ungram), HP showed higher accuracy on FSG than PSG items during the pre-training session (preference classification), whereas accuracy on FSG and PSG grammatical items was equally high in the post-training session (grammatical classification). (**b**) The two graphs illustrate the learning behavior exhibited by HP in the pre-training sessions: as they were exposed to more trials, their performance on FSG (but not PSG) trials improved. (**c**) Verbal working memory positively interacts with the identification of ungrammatical PSG (but not FSG) items.

The model fitted to the dataset including both pre-training and post-training sessions (Table 2C) showed high R^2^ (= 0.211). Although accuracy was higher in post-training (88.1%, based on transformation of log-odds to probabilities) than in pre-training (59.5%) sessions, the latter still showed above-chance accuracy. Grammaticality (ungrammatical < grammatical) and GT (PSG < FSG) were both significant as a main effect. In addition, significant interactions were found between Session and GT, and between Session, GT and Grammaticality. The plot in Figure 3a shows that accuracy on grammatical items was better for FSG than for PSG trials in the pre-training, but equal between conditions in the post-training sessions. Conversely, accuracy on ungrammatical items did not differ between FSG and PSG trials in the pre-training but was better for FSG than for PSG trials in the post-training session.

#### 3.1.3. Trial Effects during Pre-Training Sessions

As HP showed good accuracy even before training, we tested if this group presented a trial effect while performing the pre-testing sessions. Thus, the trial number was included in the basic model of the pre-training sessions as an independent variable. The trial effect was significant (see Figure 3b and Table 2E). The interaction between trial number and GT was significant (Table 2E.I), meaning that trial number was significant for FSG but not PSG trials, though it did not have an effect on grammaticality (Table 2E.II). 

#### 3.1.4. The Effect of Error Type on Performance for Ungrammatical Items

Results of the models investigating the effect of error type on accuracy reached a low R^2^ (see Table 2F). However, these results were reported because they may inform the task strategies used by HP. In the post-training sessions, error type was not significant as a main effect, but it was in interaction with GT (see Table 2F.I): permutation manipulations led to lower accuracy in PSG than in FSG, while scrambling manipulations led to lower accuracy in FSG than in PSG (for plot, see Figure S1 in Supplementary results). Table 2.F.II shows the results of models of the error effect, where the errors were differentiated between articulatory (consonant) or phonological (vocal) errors. The effect was significantly worse for articulatory than phonological in both kinds of grammar types.

#### 3.1.5. AG Generalization

The basic model showed a significant main effect only for grammaticality. The session number was not significant, meaning that accuracy did not differ between sessions (see Table 2D). The three-way interaction GT × Grammaticality × Sessions indicated that accuracy on ungrammatical FSG items was worse in S4 than in S3, while accuracy on ungrammatical PSG was better in S4 than in S3 (for plot see Supplementary results).

### 3.2. Syntax Acquisition in Left-Hemispheric Stroke Patients (Non-Ideal Native Speaker–Listeners)

The correlation matrix for all fixed factors and covariates is shown in the Supplementary data analysis. The results of each basic model are reported in the Supplementary results. Notably, all the basic models that included lesion size, TT and NIHSS score at testing time as covariates were not significant. In addition, the data set for the AGL experiment was complete (N participants = 49), while the data set for the AGG experiment included only 25 LHSP.

#### 3.2.1. The Best-Fit Model for AGL

**Figure 4:**
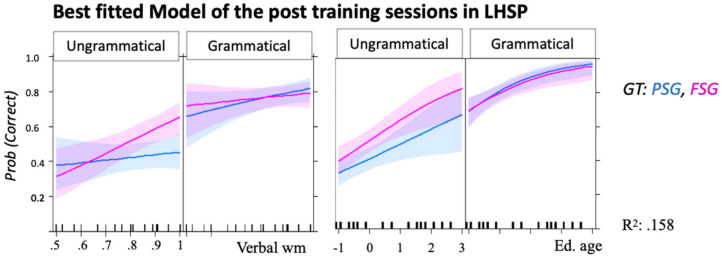
AGL in stroke condition (49 left hemispheric stroke patients, LHSP). Among the eleven possible syntax-internal and -external variables, education was the most significant predictor for grammatical classification accuracy in the post-training sessions: this effect did not interact with any other factors. Grammaticality was the second predictor, and it significantly interacted with grammar type and verbal working memory: the better the verbal WM performance, the better the accuracy of ungrammatical (but not grammatical) FSG (but not PSG) items.

The model with the lowest AIC value was: Correct~Grammar Type × Grammaticality × Working Memory + Education + (1|Participant). Figure 4 and Table 3 show the results of this model. The most significant fixed effect was education (better accuracy for higher education), followed by the Grammaticality effect (better accuracy on grammatical than on ungrammatical trials). A three-way significant interaction was found between grammaticality, GT and verbal WM, where working memory affected accuracy on ungrammatical FSG trials only (Figure 4).

As only 33/49 LHSP also had ORCP data, we calculated the best-fit model for AGL including all previous syntax factors and ORCP. The model with the lowest AIC value was the same.

#### 3.2.2. Sessions Effects in AGL

**Figure 5:**
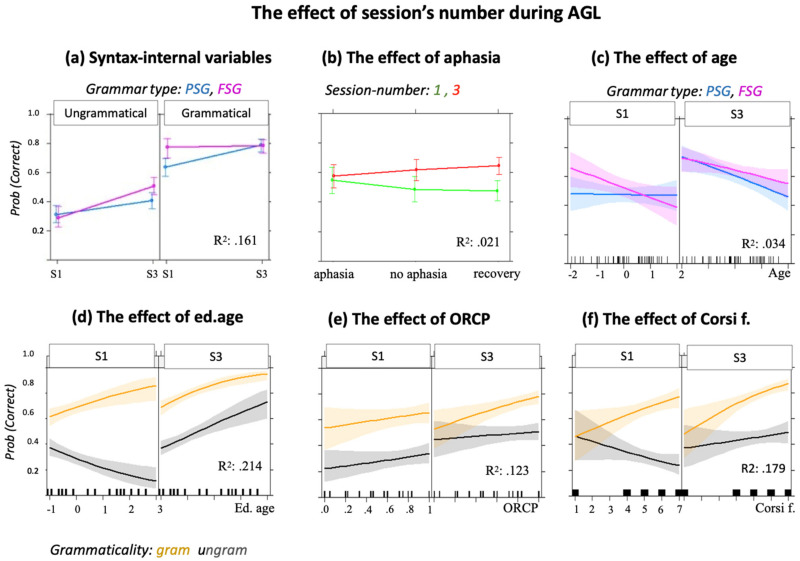
Effects of Session and syntactic variables on AGL (49 LHSP): (**a**) In the model including Session, Grammaticality (gram) and GT, accuracy in pre-training sessions was lower than accuracy in the post-training sessions. Moreover, accuracy on FSG trials was better than PSG trials for grammatical items in the pre-training sessions, but for ungrammatical items in the post-training sessions. (**b**) Effect of Session on accuracy in the three patient groups, showing better accuracy in Session 3 (red) than in Session 1 (green) in the no aphasia and the recovered groups, but not the aphasia group. (**c**) Effect of age: aging negatively interacted with FSG > PSG in pre-training sessions, and with PSG > FSG in post-training sessions. (**d**) Effect of education and (**e**) spatial working memory (Corsi f): both variables positively affected classification accuracy of grammatical items, while negatively affecting accuracy on ungrammatical items in the pre-training. (**f**) Effect of ORCP: the better the performance on ORCP, the better the grammatical classification accuracy, in the post-training sessions more so than pre-training sessions.

Figure 5a and Table 4A show the results of the basic model including the session’s number, GT and Grammaticality. Session was a significant predictor, i.e., accuracy in post-training sessions was better than in the pre-training sessions. Syntactic classification accuracy was 48.5% in the pre-training sessions and 62.9% in the post-training sessions. There was a significant three-way interaction between session number, GT, and Grammaticality (Table 4A): accuracy on PSG trials was worse than that on FSG trials, but only for grammatical trials in pre-training sessions and for ungrammatical trials in post-training sessions.

The model comparing pre- and post-training accuracy in patients with *aphasia*, without aphasia and in those who had recovered from aphasia showed a significant interaction between presence of chronic aphasia and Session (Group (= chronic) E = 0.165, SE = 0.065 Z = 2.557 *). Pairwise post-hoc comparisons indicated a significant difference in accuracy between S1 and S3 in the no aphasia group and in the recovered group, whereas the aphasia group showed no difference in performance between the two sessions (see Figure 5 and Table 4B).

Among the basic models including pre- and post-training accuracy, syntax-internal and syntax-external variables, the only external variables that showed significant fixed effects even following Bonferroni correction were education, age, and ORCP (Figure 5c–e). *Age* was a significant predictor of AGL accuracy (Table 4C,E = −0.166, SE = 0.076, z = −2.177 *, R^2^ = 0.170, in the model with grammaticality). The three-way interaction of age × GT × session was significant, indicating that age predicted accuracy (i.e., the younger, the better) on FSG at pre-training, and accuracy on PSG at post-training (Figure 5c). *Education* showed a significant fixed effect and a significant three-way interaction with Grammaticality and Session (Table 4D), indicating that individuals with the highest education showed the worst performance on ungrammatical items, and best performance on grammatical items in the pre-training sessions. In the post-training sessions, education positively affected both grammatical and ungrammatical items (Figure 5d). In the model including GT, there were no significant interactions between education and the other variables. Finally, *ORCP* was significant as a main effect, but not in interaction with other variables (Figure 5e, Table 4E.I). However, sessions interacted with grammaticality: in the model including ORCP, the difference between grammaticality and ungrammaticality was significant only in the post-training sessions. In these last sessions, ORCP interacted with Grammaticality, i.e., individuals who showed better production of object-relative sentences showed a greater increase in the classification accuracy of grammatical items more than on that of ungrammatical ones. Notably, the logistic regression showed no relation between ORCP accuracy and the presence of chronic aphasia (E = −1.841, SE = 1.278, z = −1.158).

Other syntax-external variables had no significant effect in the session number model, but showed significant interactions with syntax-internal variables as follows:

*Verbal WM* was not significant as a main effect, but it was in the interaction with Session (E = − 0.138, SE = 0.040, z = −3.032 *** in the model with GT, E = −0.103, SE = 0.044, z = −2.281 * in the model with Grammaticality): Verbal WM affected post-training, but not pre-training, sessions.

*Corsi forward* showed a significant interaction with Grammaticality (Figure 5f, Table 4F.I), meaning that Corsi positively affected accuracy on grammatical items, independently of session number. In the model with GT, there was a significant three-way interaction (Corsi × GT × Sessions (Table 4F.II)), indicating that the performance on Corsi forward negatively affected performance on PSG in the pre-training sessions, but positively in the post-training sessions (see also Supplementary results for plot). In the post-training sessions, a significant three-way Corsi × GT × Grammaticality interaction was found, indicating that the better the performance on the Corsi forward, the better the accuracy on grammatical items for PSG trials (see Table 4F.III and plot in Supplementary results).

Contrary to HP, there was no effect of trial number on accuracy during pre-training sessions in LHSP (see Supplementary results). As for the effect of the type of manipulation (scrambling vs. permutation) on accuracy for ungrammatical items, results showed a significant interaction between type of manipulation and GT, with accuracy being lower on PSG than on FSG ungrammatical items derived from permutations, and no differences between PSG and FSG ungrammatical items derived from scrambling (see Supplementary results). In addition, accuracy was lower on ungrammatical trials derived from manipulation of consonants than on those derived from manipulation of vowels (R^2^ = 0.016, E = −0.152, SE = 0.077, z = −1.971 *).

#### 3.2.3. AG Generalization

**Figure 6:**
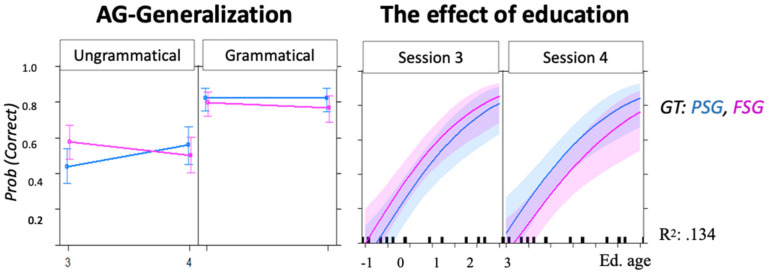
Syntax generalization (25 LHSP): AG generalization effect was significant only for the embedded grammar (in blue). This effect was mainly driven by the ungrammatical items. Educational age (ed. Age) positively influenced generalization of both GT in each session. The effect of grammar type remained significant.

Figure 6 and Table 5 show the results of the models including data from both post-training and generalization sessions: LHSP showed a significant generalization effect for PSG trials only. The interaction effect between sessions and grammar type remained significant, even when including the syntactic external variables (see Supplementary results).

The grammaticality effect was highly significant even in the models including syntax-external variables. In these models, only ORCP significantly interacted with grammaticality (E = −0.592, SE = 0.153, z = −3.875 ***), i.e., a better production of object relative sentences was positively associated with classification accuracy of grammatical items only. In this model, the effect of grammaticality alone was no longer significant (E = −0.014, SE = 0.119, z = −0.112), i.e., participants who showed poor production of object-relative sentences performed at chance on both grammatical and ungrammatical items (Supplementary results).

None of the syntax-external variables significantly interacted with the session number, meaning that their effect did not differ between post-training and generalization sessions. More specifically, within the syntax-external variables, the models including Education showed the highest R^2^ (Table 5 and Supplementary results).

## 4. Discussion

**Figure 7:**
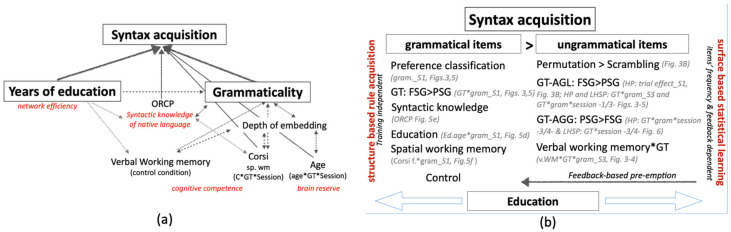
Summary of results. (**a**) List of syntax-internal and syntax-external factors that significantly predicted AGG and AGL. Black arrows display variables that were significant as a fixed effect in AGL and AGG, whereas dotted black arrows indicate interactions between variables, and gray arrows correlations between them (see Supplementary data analysis). Grammaticality and education were the most relevant predictors for syntax acquisition (see Figure 3, Figure 4, Figure 5 and Figure 6). ORCP, age and Corsi showed significant interactions with session and syntax-internal variables in the model including Sessions (Figure 5). The depth of embedding was not a predictor itself, but it interacted with Grammaticality and verbal WM (Figure 4), as well as with sessions and Corsi (PSG) or Age (FSG). Notably, some variables, such as age and education, significantly correlated, but showed different interactions. (**b**) Summary of all the significant predictors of accuracy on grammatical and ungrammatical items. The interaction with the other variables supports the dual-process account of artificial grammar acquisition.

Under both ideal and non-ideal learning conditions, and independently of the amount of exposure to stimuli (shorter vs. longer) and of task instructions (expression of preference vs. grammaticality judgment), grammatical items were always better identified than ungrammatical items. This finding suggests that adherence to UG-conformed rules promotes the ability to learn, detect and generalize novel (regular and supra-regular) syntactic rules in the absence of a lexical context. The depth of embedding was also relevant for syntax acquisition, but only in interaction with grammaticality and with other syntax-external variables. For the stroke patients, syntactic knowledge of the native language, age, working memory and education also predicted artificial grammar learning (AGL) and generalization (AGG) (Figure 7a) independent of stroke severity, lesion size and non-syntactic language impairment.

### 4.1. The Grammaticality Effect and the Dual-Process Account of AG

This study supports the dual-process account of AG syntax acquisition, as summarized in Figure 7b. As mentioned in the introduction, AGL studies have demonstrated that syntax acquisition depends, on the one hand, on prior experience and previous knowledge of syntactic rules, and on the other hand on exemplar-specific information about chunk frequencies and statistical learning (i.e., the ability to identify and memorize regularities in the linguistic input) [26,39,86]. Our results showed that both healthy and stroke participants resorted to one of these two approaches according to the syllable order in a sequence, i.e., their adherence to or violation of the UG syntactic rules.

The first difference between grammatical and ungrammatical items was evident in the pre-training sessions, where participants had to respond based on their immediate preference. Like in Forkstam et al. [77], both healthy and stroke participants showed a clear preference for grammatical items over ungrammatical items, i.e., for items adhering to familiar rules. In HP, classification accuracy of grammatical items progressively improved with increased exposure to trials, a finding that could be interpreted as if preference classification depended on item frequency. However, since LHSP lacked the trial effect during the pre-training sessions, preference for grammatical items, i.e., UG-adherence, did not depend on familiarity with local substrings.

For both healthy and LHSP groups, accuracy on unfamiliar, non UG-adherent, ungrammatical trials was poor in the pre-training sessions, but increased in the post-training sessions (Figure 3 and Figure 5). This finding showed that identification of ungrammatical trials benefitted from examples and feedback, which in turn is generally related to surface-based statistical learning [87]. Its relevance for ungrammatical items was also indicated by the better accuracy on ungrammatical trials derived from permutation rather than from scrambling in the pre-training sessions (Table 2F.I). As no feedback was provided in these sessions, this effect was probably due to an implicit statistic comparison between the first syllables group—A1B1/A1A2 for FSG and PSG, respectively—(used as possible reference) and the second syllable-group—A2B2/B2B1, which is based on perceptual similarity [88,89].

A variable that differently modulated processing of grammatical and ungrammatical items was the depth of embedding (Figure 3, Figure 4, Figure 5 and Figure 6 and Table 2, Table 3, Table 4 and Table 5). In the post-training sessions, the depth of embedding specifically predicted accuracy of ungrammatical sequences (Figure 3 and Figure 4). The finding that accuracy on FSG trials was better than that on PSG trials is in line with previous studies [13,14,88] and has been attributed to the higher frequency of FSG (vs. PSG) in written and oral language [88] or to the use of FSG across multiple domains, such as motor planning [89], music [90], and action control [41]. However, the finding that accuracy on PSG trials exceeded accuracy on the FSG trials during generalization not only in LHSP (Figure 6 and Table 5) but also in HP (Table 2D) is not in line with this explanation. Rather, it supports Chomsky’s theory that FSG and PSG relate to different syntactic structures [13], which explains the difference in complexity [14]. Given that the more representations are shaped by experience-driven learning, the more difficult it is to apply these representations to novel situations, it appears that the identification of ungrammatical FSG items relies on stimulus-specific perceptual representations, whereas identification of ungrammatical PSG item relies on stimulus-specific structural similarity.

Another variable that predicted classification accuracy of ungrammatical items after training was verbal working memory. As known from previous AGL studies, perceptual similarity between test stimuli and training stimuli benefited from memory competence, as it temporarily (during training) maintains previously presented linguistic fragments or whole instances, as well as their (implicit) recall and serial ordering during the testing tasks [77,86,91]. Our data showed first that in both our participant groups—and thus even in ideal conditions—the role of verbal WM was significant only after receiving feedback (in the post-training sessions). Second, the effect of verbal working memory was not fixed: in HP, the better the WM, the better the accuracy of PSG ungrammatical items, to the point that the difference in accuracy between grammatical and ungrammatical items disappeared. Conversely, in LHSP, the better the verbal WM performance, the better the accuracy of FSG ungrammatical items and the larger the difference between both grammar types (see Figure 4 and gram × GT × verbal WM, Table 3). The finding that verbal WM affected PSG in HP, but FSG in LHSP, indicate that the role of verbal WM was not specific to the processing of distant dependencies as often discussed [51,92], but more generally supported sensorimotor integration [93]. HP data might indicate that verbal WM positively affect the integration of long-distance dependencies, which differentiate PSG from FSG. In contrast, stroke participants presented difficulty in integrating local dependency, likely resulting from disruption of the dorsal language system [94] and causing impairments of the sensorimotor integration system [95]. In these participants, the finding that verbal WM predicted classification of ungrammatical FSG items suggests that verbal WM may help compensate for sensorimotor deficits.

In our results, verbal WM did not influence grammatical items. These findings are in line with generative grammar, which assumes that WM capacity is too limited to support the infinite number of combinations allowed by UG [3,14]. Recent studies suggested that verbal WM does not influence syntax, but instead the opposite is true [14,96,97]. Namely, they have suggested that the repeated use of specific syntactic structures may impose specific cognitive challenges to speakers, or foster specific processing habits, which in the long term might enhance specific ways of processing information beyond the linguistic domain.

Evidence for statistical learning, based on trial frequency and exemplar-specific information, was substantial for ungrammatical items, but also present for preference and classification of grammatical items in HP, as indicated by a significant effect of trial number (Figure 3, Table 2E), and in both populations by the increase in syntactic accuracy even of grammatical items after feedback was provided (Figure 3 and Figure 5 and Table 2 and Table 4). It has been argued that the trial number effect reflected the role of item familiarity [39], while feedback should allow competing familiarity signals to be contextualized [43,98,99]. According to this view, we found that trial frequency in HP increased the accuracy of grammatical classification of the most familiar grammar type, FSG (Figure 3b). We speculated that grammatical items adhering to FSG were better recognized than grammatical items generated by PSG even in stroke patients (Figure 5), who did not show a trial effect. As earlier mentioned, according to linguistics, FSG is characterized by an easier structure than PSG; therefore, we speculated that patients had a better access to FSG than to PSG.

Increasing exposure to examples and feedback improved performance mainly on PSG grammatical items in all participants (Figure 3 and Figure 5). We proposed that the role of feedback is to control grammatical judgments [43,98,99]. Through feedback, our participants (like children through parental feedback) implicitly recognized that one grammar type is the appropriate formulation in such a context, and that the other one is not appropriate, i.e., they restricted the use of FSG, strengthening the access to another, more appropriate, grammatical structure for the context, PSG. This phenomenon is labeled statistical preemption. It has been shown that statistical preemption allows people to restrict the use of a rule [43,98,99]. In keeping with this, preemption appears relevant for generalization within embedded (PSG) grammar, as the syntactic accuracy classification increased even in the generalization sessions (Figure 6 and Table 5).

The interaction of the grammaticality effect with the syntax-external variables confirmed this hypothesis. Results from the stroke group showed that, in the pre-training (but not the post-training) sessions, participants with higher education or better spatial WM showed higher accuracy on grammatical items but lower accuracy on ungrammatical items (Figure 5d,f). This finding resembles the grammaticality bias found in children, which is a well-known phenomenon in linguistic and developmental psychology [100,101,102]: children can produce ungrammatical verbs and sentences because they incorrectly apply known rules (overgeneralization). In support of this idea, after training, there was no more overgeneralization. In these sessions, education positively predicted accuracy on both grammatical and ungrammatical items, while spatial WM selectively predicted accuracy on grammatical items. The effect of education and spatial WM was similar to that of feedback, in that these factors preempted the initial excessive acceptance of items as grammatical items, by exercising a control over grammatical judgments. Chomsky has differentiated between the notion of ‘acceptable’ and the notion of ‘grammatical’ [17]. Linguistic sequences are grammatical when they are adherent to a rule, even if not meaningful, whereas linguistic sequences are ‘acceptable’ when they are meaningful within a context. As formal education provides individuals with greater exposure to reading and writing, as well as with greater sociolinguistic competence (i.e., “the competence as to when to speak, when not, and as to what to talk about with whom, when, where, in what manner” [103] p. 277), we suggest that higher education results in greater flexibility in accepting variations of a rule. Regarding the effect of spatial working memory on grammatical items, as mentioned in the introduction, the idea that spatial factors influence language has a long tradition within certain linguistic theories such as Cognitive Grammar [5,48]. In these theories, spatial organization contributes to the development of conceptual representation and some syntactic operations are represented as movement through an abstract spatial representation. Clark and Carpenter [104] found that two-year old children use the locative preposition ‘from’ correctly, when indicating the spatial or temporal origin and location of an object, but also in nonconventional cases, i.e., where adults would use the preposition by or with or because. The authors concluded that the use of ‘from’ before other propositions provides evidence that agents and causes are conceptually related to the notion of origin or starting point, direction, and spatial change. The fact that children used ‘from’ instead of ‘by’ suggests that they overgeneralize. Later in development, as children become aware that their form does not match the conventional ones, they adopt new forms from the input to express function (using ‘by’ for oblique agents). We also observed this phenomenon in our LHSP group. Before training, they classified most of the ungrammatical items as grammatical (correct). The feedback rewarded a specific spatial organization of the elements by increasing grammatical accuracy; otherwise, feedback preempted the erroneous grammar and corrected their grammatical classification. In line with this explanation, we found that Corsi performance positively influenced only items that were grammatical and adhering to PSG: it then had negative effects in the pre-training sessions, while it had positive effects in the post-training sessions (see Table 4(F.II,F.III)). It is intuitive that the schema A1A2B2B1 referring to PSG required more spatial working memory competence (organization and storage) than A1B2A2B2 referring to FSG. Support for this notion comes from functional imaging studies using eye tracking during reading or picture description. These studies showed that there is a strong connection between syntactic processes and decisions (e.g., passive structure) and eye movements (e.g., look left) [105]. Furthermore, the mean proportion and duration of fixations increased with increasing syntactic complexity [106]. To our knowledge, our results provide the first evidence that spatial working memory competence plays a significant role in knowledge-driven learning mechanisms involved during AG acquisition.

As expected, another crucial component of the knowledge-driven learning mechanism was syntactic competence in the native language, as indexed by the production of OR clauses (see [20]), which predicted accuracy on grammatical items only (Table 4(E.I,E.II)). Both OR clauses and our grammatical items adhere to UG principles. It appears that syntax acquisition is simply a matter of finding out what the local clothing is for the universal concepts we already have [5].

The effect of broad non-linguistic abilities—such as language comprehension reflected by TT performance, or the overall non-syntactic language competence tested by the AAT—was minimal for both AGL and AGG. As expected, according to previous findings [34], patients with aphasia did not increase their performance with training (Figure 5b), while also showing a preference for grammatical items in the pre-training sessions. Thus, it appears that patients with aphasia had difficulty with surface-based procedural learning, rather than with structurally based acquisition. This finding needs to be replicated, but it was not surprising, given that preceding studies have suggested that AGL paradigms may be better suited to reveal residual syntactic competence in view of their independence from lexical comprehension, which—if impaired—may prevent access to syntactic knowledge.

Taken together, the identification of novel, linguistically meaningless, grammatical items that conform to UG principles was intuitive and predicted by previous syntactic speaker’s competence, as well as by spatial knowledge. Learning to identify rule violations was mediated by item frequency and feedback and was reinforced by verbal working memory competence. In contrast to Forkstam et al. [77], who claimed that preference classification was behaviorally equivalent to the typical grammaticality classification, we found that in LHSP, feedback via preemption also strengthened performance on grammatical items. Interestingly, within LHSP, participants with aphasia did not significantly improve the classification accuracy of the grammatical PSG items after training. Post-stroke participants differed from those who never developed an aphasia or patients who recovered from it, because of impaired language competence and spatial working memory (see Table 2). Thus, damage to brain regions that support not only core language functions—which, if disrupted, cause chronic language impairment—but also spatial working memory is a crucial obstacle for learning beyond the instinctive preference for grammatical items.

### 4.2. The Effect of Education

Our study revealed that within the syntactic external variables the amount of formal education was the most relevant predictor for learning and generalizing novel AG rules. Education acted as a pusher of grammatical classification accuracy (Figure 4, Figure 5 and Figure 6).

Education correlated with age (r = 0.343, *p* = 0.016). However, the models including age explained less variance than models including education; during preference classification, education (and not age) showed a significant interaction with grammaticality, by positively affecting accuracy on grammatical items and negatively affecting that of ungrammatical items (Figure 5). On the other hand, age, but not education, showed a three-way significant interaction with GT and session, indicating [107] a negative interaction between aging and FSG particularly during the pre-training session (Figure 5). Previous studies have evaluated the effect of both demographic variables and considered age as a more direct index of brain reserve (because of age-related brain atrophy), while education has been linked to the concept of cognitive reserve (in relation to brain functionality, plasticity, and adaptability) [108]. However, it has been shown that despite age-related declines in the gray matter of language-related brain regions [108], the functionality of the language network does not show age-related decline in within-network connectivity or in responsiveness to syntactic processing demands [109,110]. Rather, aging seems associated with abnormal connectivity between the language network and the default mode network [110]. Namely, difficulties in deactivating the default mode network (DMN) during task performance lead to a more diffuse and less focal recruitment of task-relevant brain networks (e.g., [109,110]). This finding suggests that, even if the language network remains functionally intact with age, its ability to interact with other networks in the service of task goals may be affected. This could explain the variability of syntactic acquisition related to aging, although further evidence is needed. The same mechanism, i.e., an imbalance between the language network and the DMN, has been suggested as the mechanism underlying language (naming) deficits after a stroke, and the subsequent rebalancing of connectivity between the two networks as a mechanism underlying recovery from aphasia [111,112].

The contribution of education to syntax acquisition may relate more to the concept of brain efficiency. Initially, it has been discussed that individuals who have spent significant time engaged in intellectually enriching activities have a higher cognitive reserve and this reserve is linked to better functioning of brain networks [113]. Under this account, WM capacity was considered a variable mediating the relationship between brain challenge and cognitive performance. Our study found that formal education correlated with verbal working memory (r = 0.319, *p* = 0.034), but not with spatial working memory (r = 0.319, *p* = 0.034). However, we also showed that education and verbal WM interacted with grammaticality and GT in different ways, suggesting that their mechanisms of action for syntax acquisition are unlikely to be the same. Thus, we cannot exclude the possibility that formal education facilitated verbal working memory, but this last competence did not explain how and why education was a predictor for syntax acquisition. More recent data provide evidence that even healthy highly educated speakers show better production and comprehension of non-canonical sentences, and particularly ORCP [19,20,114], compared to less educated individuals. Performance of less educated HP improved dramatically after additional training on the ungrammatical syntactic constructions [115], supporting the link between syntactic processing and syntactic exposure. Considering the results of the preference classification in LHSP, the evidence of overgeneralization in highly educated stroke participants indicates that the greater exposure to constructions did not lead to better entrenched syntactic schemas, but rather to a more automatic access to known representations, i.e., education might improve linguistic performance (the ability to use linguistic knowledge) and not linguistic competence (i.e., the linguistic knowledge). In line with this interpretation, it has been shown that exposure and training of a task leads to ease of performance due to an increase in efficiency (i.e., functional connectivity, cortical thickness) of the underlying network [115].

## 5. Conclusions

The present study indicates that all (ideal and non-ideal) native speakers learned to better classify novel linguistic sequences based on universal syntactic construction type compared to sequences violating such constructions.

Our data confirmed that, on the one hand, GT was not a predictor for syntax acquisition in itself and, on the other hand, the two kinds of grammar acted differently. FSG appears to be more related to a surface-based learning mechanism, as grammatical items referring to the easier [14,15,116] and likely more frequent GT (i.e., FSG) were preferred to the more complex and less frequent (i.e., PSG [114]) ones. In addition, FSG items responded to increased trial exposure (at least in HP) and mostly responded to the training-induced feedback (in both HP and LHSP). Such a mechanism was supported in LHSP by verbal working memory, which facilitates the recognition of syntactic violations by maintaining the elements during sensorimotor integration. PSG seems to be more linked to knowledge-driven learning mechanisms and, therefore, could be better generalized. Structural knowledge was linked not only to linguistic competence (as the ability to produce object relative sentences) but also to spatial knowledge, as indicated by the finding that both previous syntactic and spatial knowledge predicted the ability to identify specific syntactic constructions in the novel stimuli. Most importantly, patients’ data provide evidence that linguistic acquisition depends on the amount of formal education. Education improved linguistic performance, strengthening the ability to learn and generalize novels, as well as to recognize UG rules and their violations in a novel context.

## 6. Limitations

It should be noted that the group of individuals with aphasia was small (*n* = 14) and heterogeneous; therefore, all our results relating to this population group should be verified in future studies. In particular, it could be very interesting to prove the relationship between spatial working memory, which in our participants with aphasia was worse performed than in participants without aphasia (but not in [49]), and syntax acquisition.

Moreover, we discussed in the manuscript that stroke participants presented with difficulty in integrating local dependency likely resulting from disruption of the dorsal language system [94] and causing impairments of the sensorimotor integration system [95]. Further functional imaging studies should prove to what extent specifically FSG deficits in LHSP are related to the dorsal system (in HP, Friederici et al. [74] claimed that only PSG is related to the dorsal system). Moreover, functional imaging data are necessary to demonstrate the relationship between functional connectivity, specifically within the syntax network, and age or the level of education.

Another important limitation of this study was the absence of the intelligent quotient. It is well known that such a quotient could influence the level of education, so that the observed effects could be more related to intelligence (genetic factors) than to education (culture). The debate on this point is hotly disputed and controversial. Unfortunately, our results could not contribute to this topic.

## Figures and Tables

**Table 1:** Patients’ demographic and clinical data.

Variable	Groups	Descriptive Statistics					One-Way ANOVA			
		N	Mean	SD	Range	Shapiro-Wilk *p*	F	df1	df2	*p*
Age	Aphasia	14	64.4	11.8	42	0.816	0.004	2	46	0.996
	Recovery	20	64.8	14.7	50	0.064				
	no Aphasia	15	64.5	12.8	48	0.485				
Education	Aphasia	14	12.6	3.4	11	0.557	0.402	2	46	0.671
	Recovery	20	13.2	4.2	14	0.003				
	no Aphasia	15	13.8	2.9	10	<0.001				
Lesion Vol nativ	Aphasia	14	46.9	58.9	216	0.006	4.246	2	46	0.020
	Recovery	20	24.9	29.0	119	<0.001				
	no Aphasia	15	7.0	10.7	34	<0.001				
NIHSS	Aphasia	14	2.1	1.4	4	0.002	3.036	2	46	0.058
	Recovery	20	0.4	1.0	4	<0.001				
	no Aphasia	15	1.5	3.0	10	<0.001				
Corsi	Aphasia	14	4.6	1.2	5	<0.001	4.229	2	46	0.021
	Recovery	20	5.4	0.9	3	0.023				
	no Aphasia	15	5.6	0.9	3	0.025				
TT	Aphasia	14	65.6	9.9	27	0.002	7.643	2	46	0.001
	Recovery	20	72.2	2.1	7	<0.001				
	no Aphasia	15	72.7	1.0	4	<0.001				
							**Kruskal-Wallis Test**			
							**χ^2^**	**df**		***p***
Syntax comprehension	Aphasia	8	0.91	0.15	0.40	0.003	2.579	2		0.275
	Recovery	13	0.96	0.06	0.15	<0.001				
	no Aphasia	12	0.99	0.05	0.17	<0.001				
Syntax production	Aphasia	8	0.78	0.17	0.52	0.009	0.949	2		0.622
	Recovery	13	0.88	0.17	0.52	0.002				
	no Aphasia	12	0.90	0.11	0.33	0.047				
ORCP	Aphasia	8	0.69	0.40	1.00	0.033	0.479	2		0.787
	Recovery	13	0.80	0.33	0.95	<0.001				
	no Aphasia	12	0.8	0.25	0.67	0.007				
							**χ^2^-Test**			
						**χ^2^-Test** ***p***	**χ^2^**	**df**		*p*
Gender	Aphasia	14	4	10		0.109	0.467	2		0.977
	Recovery	20	6	14		0.074				
	no Aphasia	15	4	11		0.071				

**Table 2:** HP results. Fixed effects and their interactions for all relevant statistical models.

A. Best Fitted Model (S3) R^2^ = 0.081, RI = 0.846				B. Verbal Working Memory S3 R^2^ = 0.095, RI = 0.836			
Predictor	Log (Odds)	SE	z-Value	Predictor	Log (Odds)	SE	z-Value
Intercept	2.165	0.169	12.807 ***	Intercept	2.175	0.168	12.94 ***
GT (=E)	−0.089	0.083	−1.084	vwm	0.137	0.162	0.844
**Gram (=no)**	**−0.564**	**0.083**	−6.784 ***	**Gram (=no)**	**−0.562**	**0.084**	−6.697 ***
**GT × Gram.**	**−0.165**	**0.083**	−1.998 *	GT (=E)	−0.09	0.083	−1.077
				GT × Gram	−0.153	0.083	−1.83
				GT × vwm	−0.006	0.08	−0.077
				Gram × vwm	0.11	0.081	1.36
				**GT × gram × vwn**	**0.194**	**0.08**	2.419 *
**C Session-number (S3, S1) R^2^ = 0.211, RI = 0.339**				**D. Generalization (S3, S4) R^2^ = 0.007, RI = 0.853**			
Predictor	Log (Odds)	SE	z-Value	Predictor	Log (Odds)	SE	z-Value
Intercept	1.198	0.075	15.998 ***	Intercept	2.233	0.157	14.212
**Session (=1)**	**−0.763**	**0.05**	−15.356 *	GT (=E)	−0.052	0.06	−0.861
**G T (=E)**	**−0.218**	**0.049**	−4.408 **	**Gram (=0)**	−0.533	**0.06**	−8.846 ***
**Gram (=no)**	**−0.516**	**0.05**	−10.427 ***	Session (=3)	−0.074	0.06	−1.228
**Session × GT**	**−0.139**	**0.049**	−2.806 **	GT × S	−0.018	0.06	−0.603
S × Gram	0	0.049	−0.003	Gram × S	−0.02	0.06	−0.603
GT × Gram	−0.007	0.049	−0.145	Gram × GT	−0.018	0.06	−0.297
**GT × Gram × S**	**0.144**	**0.049**	2.913 **	**GT × Gram × S**	**−0.145**	**0.06**	−2.424 *
**E.I Trial Effect_S1 R^2^ = 0.041, RI = 0.140**				**E.II. Trial Effect_S1 R^2^= 0.07, RI = 0.143**			
Predictor	Log (Odds)	SE	z-Value	Predictor	Log (Odds)	SE	z-Value
Intercept	0.085	0.111	0.765	Intercept	0.093	0.115	0.809
GT (=E)	−0.03	0.109	−0.277	**Gram (=no)**	**−0.568**	**0.112**	−5.05 ***
**Trial**	**0.028**	**0.009**	3.166 **	**Trial**	**0.028**	**0.009**	3.118 **
**GT × Trial**	**−0.024**	**0.009**	−2.754 **	Gram × Trial	0.009	0.009	0.942
**F.I Error_type (ET)_S3 R^2^ = 0.066, RI = 0.946**				**F.II Error code (EC)_S3 R^2^ = 0.063, RI = 0.908**			
Predictor	Log (Odds)	SE	z-Value	Predictor	Log (Odds)	SE	z-Value
Intercept	1.618	0.191	8.459 ***	Intercept	1.591	0.185	8.607 ***
**GT (=E)**	**−0.22**	**0.095**	−2.309 *	**GT (=E)**	**−0.209**	**0.095**	−2.205 *
ET (=Perm)	0.075	0.095	0.784	EC (=Art)	**−0.463**	**0.095**	−4.856 ***
**ET × GT**	**−0.476**	**0.096**	−4.956 ***	ET × GT	0.064	0.095	0.681

Abbreviation: Std. Error = standard error, GT: grammar type, Gram = grammaticality, S = session number. Significance was indicated with * for *p* < 0.05, ** for *p* < 0.01, and *** for *p* < 0.0001. RI = Random intercept SD.

**Table 3:** Fixed effects and their interactions derived from the best-fit model for post-training sessions in LHSP.

Best Fitted Model R^2^ = 0.158, RI = 0.575			
Predictor	Log (Odds)	SE	z-Value
Intercept	0.009	0.59	0.015
GT (=E)	0.166	0.313	0.53
**Gram (=no)**	**−0.816**	**0.316**	−2.582 **
vwm	0.692	0.69	1.003
**Ed. age (z-t)**	**0.409**	**0.117**	3.507 ***
GT × Gram	0.525	0.311	1.69
GT × Ed.age	−0.327	0.368	−0.888
**GT × Gram × vwm**	**−0.771**	**0.364**	−2.114 *

Abbreviation: Std. Error = standard error, GT: grammar type, Gram = grammaticality, S = session number. Significance was indicated with * for *p* < 0.05, ** for *p* < 0.01, and *** for *p* < 0.0001. RI = Random intercept SD.

**Table 4:** LHSP results. Fixed effects and their interactions for all the models describing performance in LHSP.

A. Session-Number (S1 vs. S3) R^2^ = 0.167, RI = 0.443				B. Aphasia S1, S3 R^2^ = 0.021, RI = 0.468				C. Age S1, S3 R^2^ = 0.034, RI = 0.421			
Predictor	Log(Odds)	SE	z-Value	Predictor	Log(Odds)	SE	z-Value	Intercept	0.279	0.075	3.406 ***
Intercept	0.293	0.08	3.67 ***	Intercept	0.24	0.082	2.923 **	**GT (=0)**	**−0.773**	**0.044**	−2.167 *
**GT (=E)**	**−0.117**	**0.046**	−2.535 *	Chronic Aphasia	0.011	0.121	0.088	**Age**	**−0.116**	**0.070**	−2.570 *
**Gram (=no)**	**−0.803**	**0.046**	−17.408 ***	no Aphasia	−0.003	0.117	−0.028	**Session (=1)**	**−0.257**	**0.044**	−5.942 ***
**Session N.**	**−0.268**	**0.047**	−5.709 ***	**Session (=1)**	**−0.227**	**0.043**	−5.523 ***	Session × GT	−0.009	0.044	−0.220
S × GT	−0.022	0.046	−0.475	Aphasia * Session	**0.176**	**0.065**	2.848 *	Age × GT	0.054	0.045	1.188
GT × Gram	0.041	0.046	0.902	no Aphasia × Session	−0.035	0.063	−0.578	Session × age	0.055	0.045	1.143
S × Gram	−0.068	0.046	−1.475	*Pairwaise S3 vs. S1*				**GT × S × age**	**0.09**	**0.044**	2.157 *
**S × Gram × GT**	**0.148**	**0.046**	3.232 **	**no aphasia**	**−0.542**	**0.157**	−3.459 ***				
				**recovery**	**−0.750**	**0.124**	−5.815 ***				
				aphasia	0.155	−0.761	0.974				
**D. Educational age S1, S3 R^2^ = 0.214, RI = 0.373**				**E.I ORCP S1, S3 R^2^ = 0.123, RI = 0.452, RI = 0.439**				**E.II ORCP S3 R^2^ = 0.978, RI = 0.717**			
Intercept	0.272	0.071	3.805 ***	Intercept	−0.302	0.25	−1.206	Intercept	0.373	0.808	−0.317
**Gram (=no)**	**−0.800**	**0.045**	−17.501 ***	**Gram (=0)**	**−0.433**	**0.128**	−3.384 ***	Gram (=0)	−0.152	0.159	−0.954
**Session N.**	**−0.322**	**0.046**	−6.962 ***	**ORCP**	**0.624**	**0.304**	2.056 *	ORCP	0.803	0.452	1.778
**Ed. age (z-t)**	**0.222**	**0.074**	2.973 **	**Session (=1)**	**0.254**	**0.129**	−1.968 *	GT	−0.196	0.161	−1.125
S × Gram	−0.050	0.045	−1.118	**Session × Gram**	**−0.27**	**0.128**	−2.115 *	**ORCP × Gram**	**−0.499**	**0.197**	−2.535 *
**Gram × Ed.age**	**−0.224**	**0.048**	−4.603 ***	ORCP × Gram	−0.203	0.155	−1.34	ORCP × GT	0.064	0.198	0.323
**S × Ed.age**	**−0.249**	**0.049**	−5.079 ***	S × ORCP	−0.079	0.157	−0.506	GT × gram	0.056	0.159	0.352
**S × Gram× Ed.age**	**−0.140**	**0.049**	−2.874 **	S × Gram × ORCP	0.247	0.155	1.559	ORCP × GT × gram	−0.186	0.196	0.402
**F.I Corsi f. S1, S3 R^2^ = 0.179, RI = 0.4296**				**F.II Corsi f. S1, S3 R^2^ = 0.031, R.I. = 0.439**				**F.III Corsi f. S3 R^2^ = 0.174, RI = 0.708**			
Intercept	0.267	0.077	3.454 ***	Intercept	0.247	0.077	3.223 **	Intercept	−0.612	0.58	−1.055
**Gram (=no)**	**−0.786**	**0.045**	−17.556 ***	**GT (=E)**	**−0.096**	**0.042**	−2.287 *	**GT (E)**	**−0.909**	**0.269**	−3.375 ***
**Session N.**	**−0.302**	**0.045**	−6.653 ***	**Session N.**	**−0.244**	**0.042**	−5.703 ***	**Corsi (forward)**	**0.236**	**0.11**	2.149 *
Corsi f.	0.133	0.080	1.670	Corsi f.	0.126	0.080	1.582	**Gram (=no)**	**−0.02**	**0.269**	**−0.076**
S × Gram	−0.039	0.444	−0.885	S × GT	−0.006	0.043	−0.164	**GT × Corsi**	**0.162**	**0.052**	3.11 **
**Gram × Corsi**	**−0.118**	**0.042**	−4.367 ***	GT × Corsi	0.399	0.040	0.976	GT × Gram	0.47	0.267	1.758
**S × Corsi**	**−0.103**	**0.428**	−2.422 *	S × Corsi	−0.077	0.041	−1.186	**Corsi × Gram**	**−0.148**	**0.052**	−2.847 **
S × Gram × Corsi	−0.043	−0.043	0.303	**S × GT × Corsi**	**−0.109**	**0.040**	−2.690 **	**GT × Corsi × Gram**	**−0.118**	**0.052**	−2.284 *

Abbreviations: Std. Error = standard error, GT: grammar type, Gram = grammaticality, S = session number, Gr = groups, P = LHSP, ORCP: Object relative clause production, Corsi f.: Corsi forward. Significance was indicated with * for *p* < 0.05, ** for *p* < 0.01, and *** for *p* < 0.0001. RI = Random intercept SD.

**Table 5:** AGG results in LHSP. Fixed effects and their interactions were listed.

A. Generalization (S4, S3) R^2^ = 0.0118, RI = 0.814				B. Education (S4, S3) R^2^ = 0.134, RI = 0.657			
Predictor	Log (Odds)	SE	z-Value	Predictor	Log (Odds)	SE	z-Value
Intercept	0.063	0.123	4.979 ***	Intercept	0.737	0.124	5.928 ***
GT (=E)	0.016	0.051	0.422	GT (=E)	0.023	0.049	0.463
**Gram (=0)**	**−0.068**	**0.046**	−14.856 ***	**Ed.age**	**0.794**	**0.141**	5.64 ***
Session (=3)	−0.005	0.047	−0.121	Session (=3)	−0.006	0.049	−0.117
**GT × S**	**−0.07**	**0.049**	−2.534 *	**GT × S**	**−0.135**	**0.049**	−2.739 **
Gram × S	−0.044	0.048	−1.542	S × Ed.Age	0.044	0.063	0.708
Gram × GT	−0.099	0.049	−1.549	GT × Ed.Age	0.014	0.063	0.224
GT × S × Gram	−0.076	0.049	−1.052	GT × S × Ed.Age	−0.025	0.063	−0.395

Abbreviation: Std. Error = standard error, GT: grammar type, Gram = grammaticality, S = session number. Significance was indicated with * for *p* < 0.05, ** for *p* < 0.01, and *** for *p* < 0.0001. RI = Random intercept SD.

## Data Availability

All the data anaylsis and all random end fixed effect analysis presented in this study are reported in Supplementary data analysis and Supplementary results respectively. Individual raw data (including fMRI) are available on request due to ethical restrictions.

## Supplementary Materials

Supplementary materials can be downloaded from: https://www.mdpi.com/article/10.3390/brainsci12050616/s1. Supplementary data analysis included: Table S1: Data distribution, Figure S1: Results of collinearity analysis, Figure S2: Results of descriptive statistics, Table S2: Results of correlation analysis in HP: A. Correlation across predictors(r), B. Correlation across predictors (p-values), C. Variance Inflaction Factor; Table S3: Results of correlation analysis in LHSP: A. Correlation across predictors(r), B. Correlation across predictors (*p*-values), C. Variance Inflaction Factor; Figure S3 Model Fit checks: A. Best fitted Model in LHSP, B. Best fitted Model in HP, C: Model with the variable Aphasia and session-number; D. Model with the variable ORCP and session-number, and GT (D1), or grammaticality (D2), and model with ORCP, GT and grammaticality_S3 (D3); E Model with the variable Educational age and session-number, and GT (E1), or grammaticality (E2), and model with educational age, GT and grammaticality_S1 (E3) and in S3 (E4); F Model with the variable Corsi f. and session-number, and GT (F1), or grammaticality (F2), and model with educational age, GT and grammaticality_S1 (F3) and in S3 (F4). Supplementary results included: Figure S1: The effect of Error type, Figure S2: The Effect of generalization in HP, Figure S3: The effect of external predictors in LHSP. Figure S4: The effecto of Corsi. Table S1: Syntax-internal predictors in LHSP in S3 (A), S1 and S3 (B), Table S2: The Effect of Error type in S3 in HP and LHSP; Table S3: Fixed effects results of the logistic regression analysis conducted on the model including: A. Age, B: Education; C: Gender; D: verbal working memory; E: spatial working memory (Corsi f. and backward), F.: Stroke severity; G: Difference between NIHSS at testing and NIHSS_admission (acute stroke phase), H Aphasia, I: ORCP. Table S4: Fixed effects results of the logistic regression analysis for S3 and 4 (generalization) conducted on the model including: a) verbal working memory, B, Age; C: Corsi f., D: Corsi backward, E: Gender; F: Lesion size, G: NIHSS difference; H= Presence of aphasia in T1, I: ORCP.

## Appendix A

Lesion normalization was performed as follows:], the ischemic lesions were first delineated on the diffusion-weighted images based on intensity thresholds using a customized region-of-interest toolbox implemented in SPM8 (http://www.fil.ion.ucl.ac.uk/spm/software/spm8, accessed on 2 March 2015) and manually refined by specially trained research assistants who were blind to the patient’s clinical scores. Prior to normalization, the exact correspondence between lesion map and lesion was checked. Lesion mapping and inspection were performed in MRIcron. (http://www.cabiatl.com/mrico/mricon/stats.html, accessed on 2019). For normalization, the diffusion-weighted images and corresponding lesion maps were co-registered to the anatomical T1 scan. The T1 scans were segmented using the VBM8 toolbox (r435; http://dbm.neuro.uni-jena.de/vbm/download/, accessed on 2015), and deformation field parameters for nonlinear normalization into the stereotactic Montreal Neurological Institute (MNI) standard space were obtained using the DARTEL approach (diffeomorphic anatomical registration through exponentiated lie algebra) implemented in VBM821. Following normalization, the individual lesion maps were again inspected and compared to the lesions in native subject space to ensure that the extent of the ischemic damage was delineated accurately in MNI space.
