# Supplementary material for: Syntax Acquisition in Healthy Adults and Post-Stroke Individuals: The Intriguing Role of Grammatical Preference, Statistical Learning, and Education
Source: Brain Sci. 2022 May 9;12(5):616. doi: 10.3390/brainsci12050616 (PMC9139563; doi:10.3390/brainsci12050616)

Supplementary data analysis

Table S1: Data Distribution

Variable	Groups	Descriptive statistics				
		N	Mean	SD	Range	Shapiro-Wilk
Age	Control	37	25.8	5.88	27.0	<.001
	Patient	49	64.6	13.1	53.3	0.067
Edu Age	Control	37	18.2	3.27	13.0	0.317
	Patient	49	13.3	3.60	15.0	<.001
Lesion Vol nativ	Control					
	Patient	47	15.7	23.2	102	<.001
Lesion Vol hd	Control					
	Patient	49	25.7	39.3	217	<.001
NIHSS	Control					
	Patient	49	1.22	2.02	10	<.001
Corsi	Control					
	Patient	49	5.20	1.06	6	<.001
TT	Control					
	Patient	49	82.4	29.6	99	<.001
Gender	Control	37				<.001
	Patient	49				<.001
Syntax comprehensio	Control					
	Patient	33	0.958	0.089	0.400	<.001
Syntax production	Control					
	Patient	33	0.864	0.192	0.800	<.001
ORCP	Control					
	Patient	33	0.859	0.201	0.600	<.001

Figure S1: Results of collinearity analysis

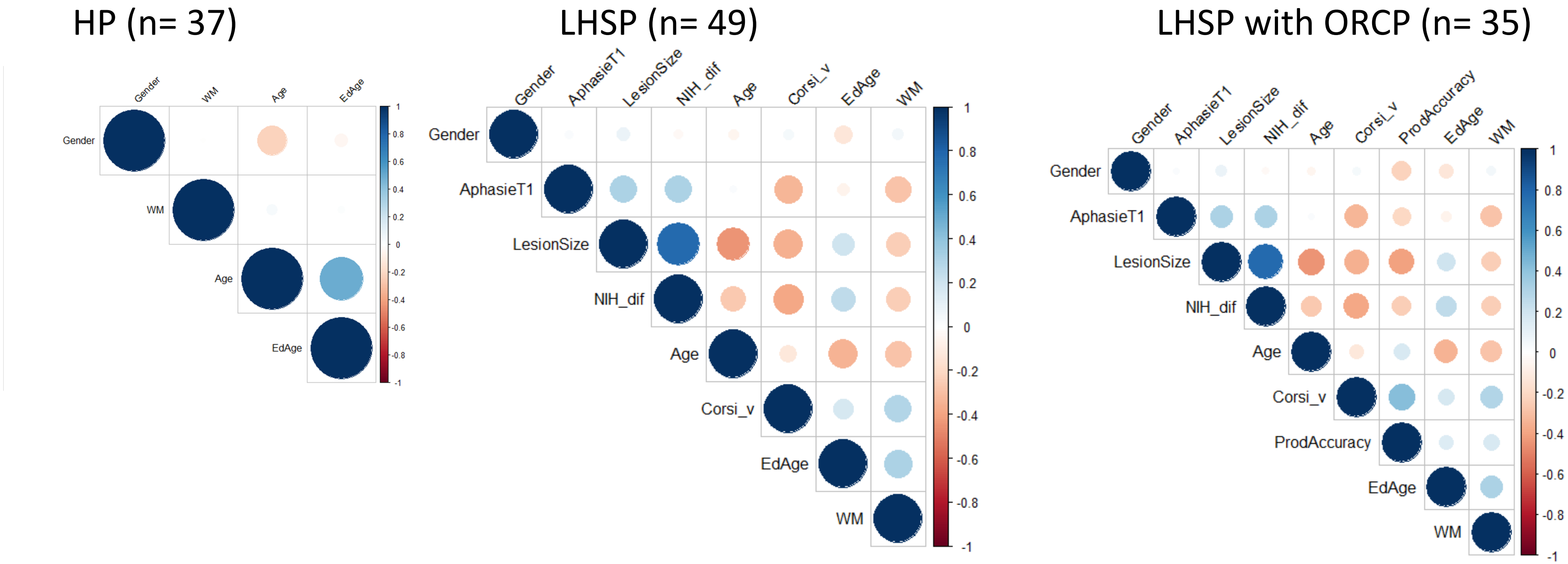

Figure S2: Results of descriptive statistics

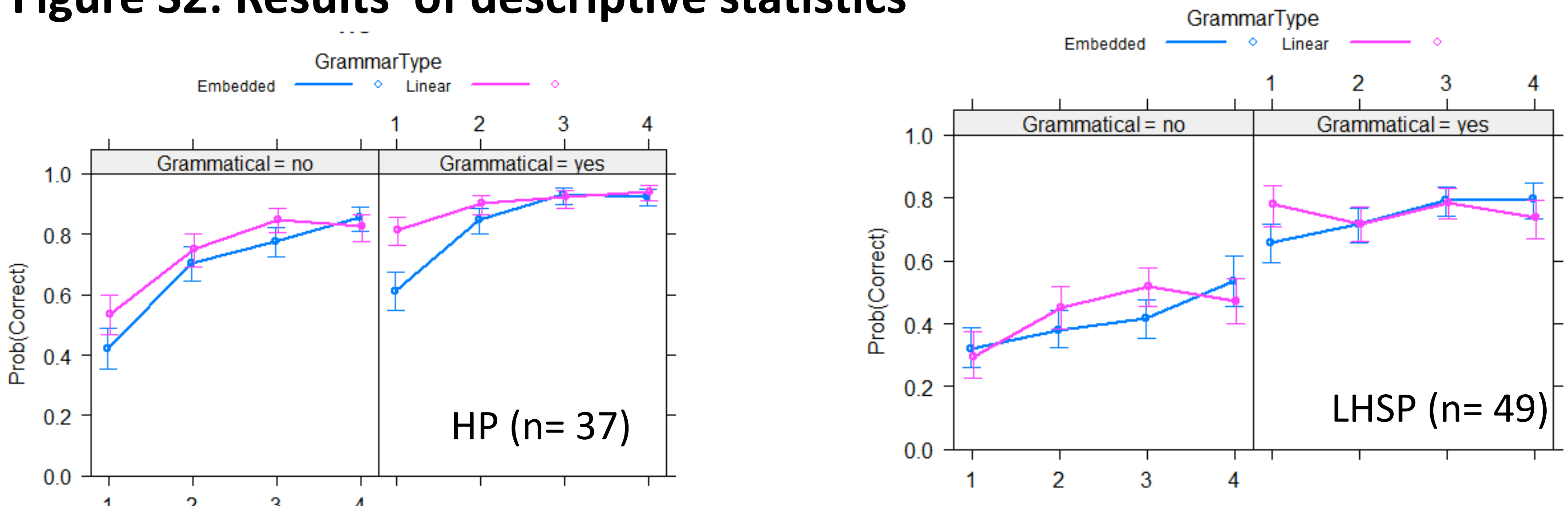

Table S2: Results of correlation analysis: HP

A: Correlation across predictors (r)

	Age	EdAge	Gender	WM
Age	1.00000000	0.49604194	-0.229705952	0.030850146
EdAge	0.49604194	1.00000000	-0.047549133	0.013578601
Gender	-0.22970595	-0.04754913	1.000000000	-0.005865605
WM	0.03085015	0.01357860	-0.005865605	1.000000000

B: Correlation across predictors (p-values)

	Age	EdAge	Gender	WM
Age	NA	0.001794693	0.1714189	0.8561664
EdAge	0.001794693	NA	0.7798910	0.9364249
Gender	0.171418911	0.779891046	NA	0.9725145
WM	0.856166380	0.936424882	0.9725145	NA

C: Variance Inflation Factor

GrammarType	grammatical	scaleAge	scaleEdAge	GenderBin	WorkingMemory
1.000486	1.001107	1.350353	1.279699	1.063885	1.001709

Table S3：Results of correlation analysis in LHSP (n=49)

A: Correlation across predictors (r)

	AphasieT1	LesionSize	Age	EdAge	Gender	WM	NIH_dif	Corsi_v
AphasieT1	1.00000000	0.31320038	0.02566519	-0.06943026	0.02923527	-0.28408907	0.31469671	-0.33711464
LesionSize	0.31320038	1.00000000	-0.44359804	0.20837150	0.08018258	-0.24751302	0.77463981	-0.35753493
Age	0.02566519	-0.44359804	1.00000000	-0.34325052	-0.05186846	-0.28671309	-0.26326914	-0.12527656
EdAge	-0.06943026	0.20837150	-0.34325052	1.00000000	-0.13505999	0.31974955	0.25086767	0.17777236
Gender	0.02923527	0.08018258	-0.05186846	-0.13505999	1.00000000	0.05327427	-0.03703309	0.04919934
WM	-0.28408907	-0.24751302	-0.28671309	0.31974955	0.05327427	1.00000000	-0.24584279	0.29916262
NIH_dif	0.31469671	0.77463981	-0.26326914	0.25086767	-0.03703309	-0.24584279	1.00000000	-0.38975688
Corsi_v	-0.33711464	-0.35753493	-0.12527656	0.17777236	0.04919934	0.29916262	-0.38975688	1.00000000

B: Correlation across predictors (p-values)

	AphasieT1	LesionSize	Age	EdAge	Gender	WM	NIH_dif	Corsi_v
AphasieT1	NA	2.843270e-02	0.861042548	0.63547277	0.8419449	0.06163732	2.764429e-02	0.017851619
LesionSize	0.02843270	NA	0.001410565	0.15078296	0.5839174	0.10526077	6.538214e-11	0.011665185
Age	0.86104255	1.410565e-03	NA	0.01575297	0.7233804	0.05917140	6.759448e-02	0.391067142
EdAge	0.63547277	1.507830e-01	0.015752965	NA	0.3548293	0.03436045	8.209388e-02	0.221691018
Gender	0.84194485	5.839174e-01	0.723380376	0.35482929	NA	0.73125819	8.005572e-01	0.737090988
WM	0.06163732	1.052608e-01	0.059171404	0.03436045	0.7312582	NA	1.077068e-01	0.048526232
NIH_dif	0.02764429	6.538214e-11	0.067594479	0.08209388	0.8005572	0.10770679	NA	0.005635431
Corsi_v	0.01785162	1.166519e-02	0.391067142	0.22169102	0.7370910	0.04852623	5.635431e-03	NA

C: Variance Inflation Factor

GrammarType	Grammatical	AphasieT1	scale_Lesion	scale_Age	scale_edAge	WorkingMemory	Gender	NIH_dif	Corsi_v_Spanne
1.003920	1.005222	1.253112	4.143805	1.769536	1.467916	1.600611	1.098022	3.115143	1.563650

Table S4：Results of correlation analysis in LHSP (n= 33)

A: Correlation across predictors (r)

	AphasieT1	LesionSize	Age	EdAge	Gender	WM	NIH_dif	Corsi_v	ProdAccuracy
AphasieT1	1.00000000	0.31320038	0.02566519	-0.06943026	0.02923527	-0.28408907	0.31469671	-0.33711464	-0.2073848
LesionSize	0.31320038	1.00000000	-0.44359804	0.20837150	0.08018258	-0.24751302	0.77463981	-0.35753493	-0.4007380
Age	0.02566519	-0.44359804	1.00000000	-0.34325052	-0.05186846	-0.28671309	-0.26326914	-0.12527656	0.1660887
EdAge	-0.06943026	0.20837150	-0.34325052	1.00000000	-0.13505999	0.31974955	0.25086767	0.17777236	0.1416543
Gender	0.02923527	0.08018258	-0.05186846	-0.13505999	1.00000000	0.05327427	-0.03703309	0.04919934	-0.2288182
WM	-0.28408907	-0.24751302	-0.28671309	0.31974955	0.05327427	1.00000000	-0.24584279	0.29916262	0.1669942
NIH_dif	0.31469671	0.77463981	-0.26326914	0.25086767	-0.03703309	-0.24584279	1.00000000	-0.38975688	-0.2404316
Corsi_v	-0.33711464	-0.35753493	-0.12527656	0.17777236	0.04919934	0.29916262	-0.38975688	1.00000000	0.4264431
ProdAccuracy	-0.20738482	-0.40073802	0.16608872	0.14165434	-0.22881820	0.16699419	-0.24043165	0.42644308	1.0000000

B: Correlation across predictors (p-values)

	AphasieT1	LesionSize	Age	EdAge	Gender	WM	NIH_dif	Corsi_v	ProdAccuracy
AphasieT1	NA	2.843270e-02	0.861042548	0.63547277	0.8419449	0.06163732	2.764429e-02	0.017851619	0.24684661
LesionSize	0.02843270	NA	0.001410565	0.15078296	0.5839174	0.10526077	6.538214e-11	0.011665185	0.02082670
Age	0.86104255	1.410565e-03	NA	0.01575297	0.7233804	0.05917140	6.759448e-02	0.391067142	0.35561303
EdAge	0.63547277	1.507830e-01	0.015752965	NA	0.3548293	0.03436045	8.209388e-02	0.221691018	0.43166656
Gender	0.84194485	5.839174e-01	0.723380376	0.35482929	NA	0.73125819	8.005572e-01	0.737090988	0.20024632
WM	0.06163732	1.052608e-01	0.059171404	0.03436045	0.7312582	NA	1.077068e-01	0.048526232	0.36097163
NIH_dif	0.02764429	6.538214e-11	0.067594479	0.08209388	0.8005572	0.10770679	NA	0.005635431	0.17772680
Corsi_v	0.01785162	1.166519e-02	0.391067142	0.22169102	0.7370910	0.04852623	5.635431e-03	NA	0.01333376
ProdAccuracy	0.24684661	2.082670e-02	0.355613030	0.43166656	0.2002463	0.36097163	1.777268e-01	0.013333765	NA

C: Variance Inflation Factor

GrammarType	Grammatical	AphasieT1	scale_Lesion	scale_Age	scale_edAge	WorkingMemory	Gender
1.005196	1.004529	1.443696	5.452832	2.430785	1.754624	1.907535	1.412574

NIH_dif	Corsi_v_Spanne	ProdAccuracy
3.715008	2.041651	1.517982

Figure S3 Model Fit checks:

A. Best Fitted Model (LHSP):
Correct ~ GrammarType * Grammatical * WorkingMemory + scale_edAge + (1|Subject)

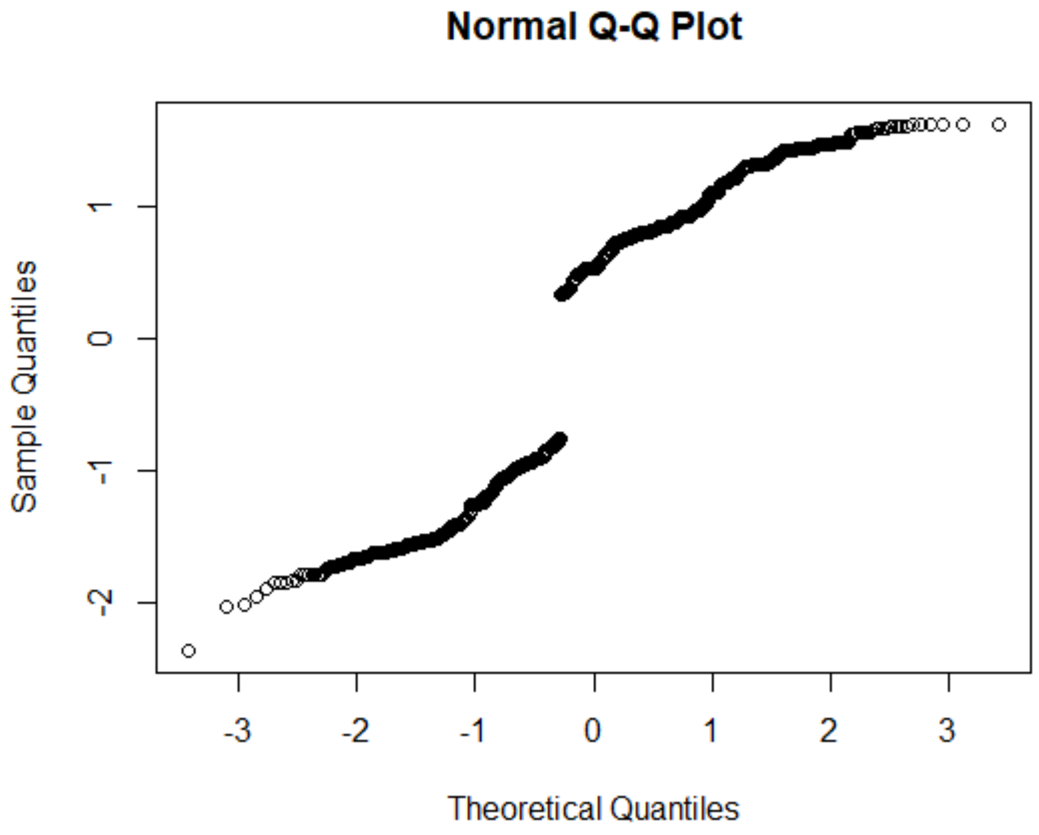

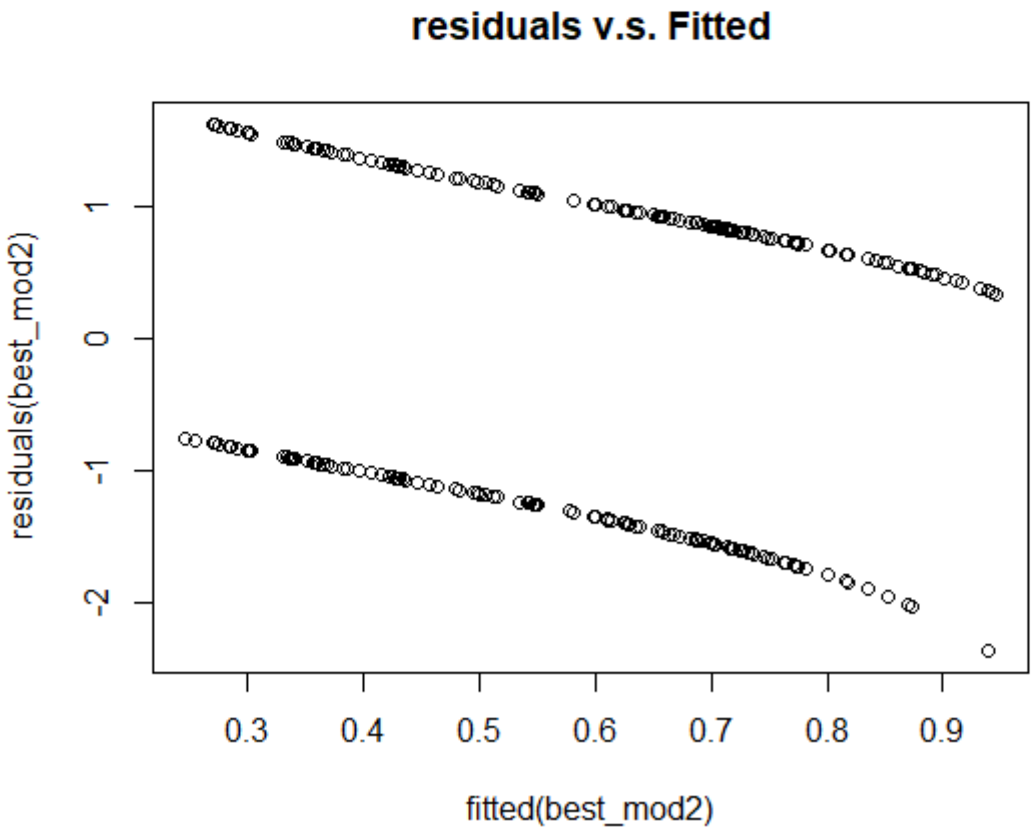

B. Best Fitted Model (HC):
Correct ~ GrammarType * Grammatical + (1|Subject)

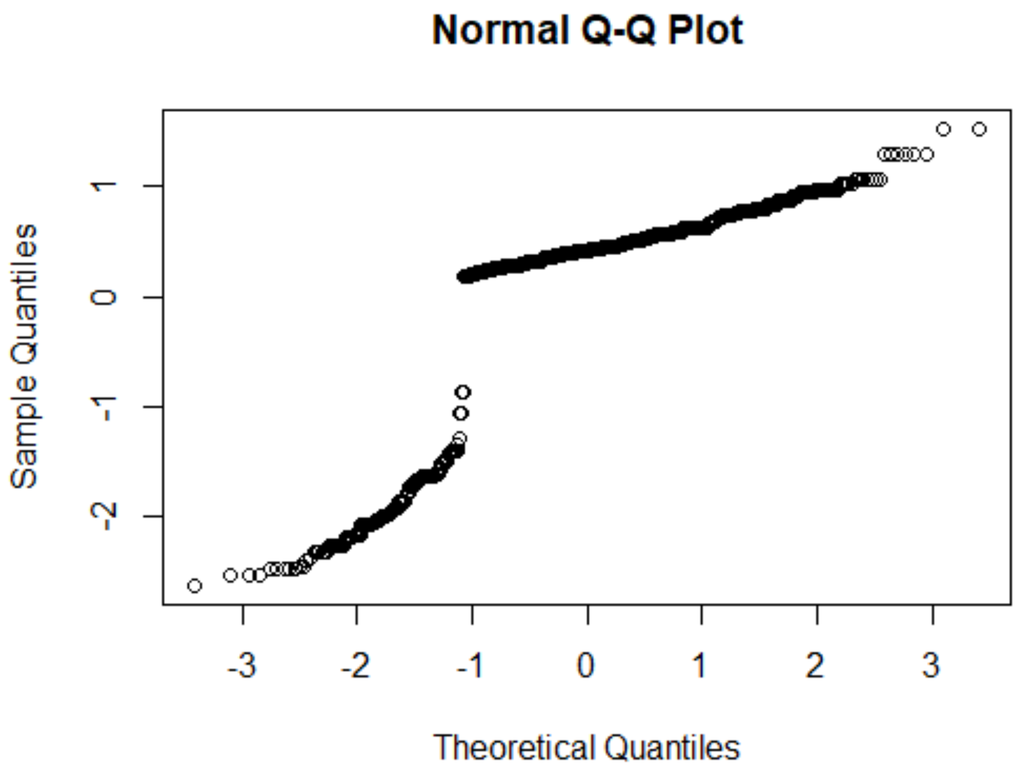

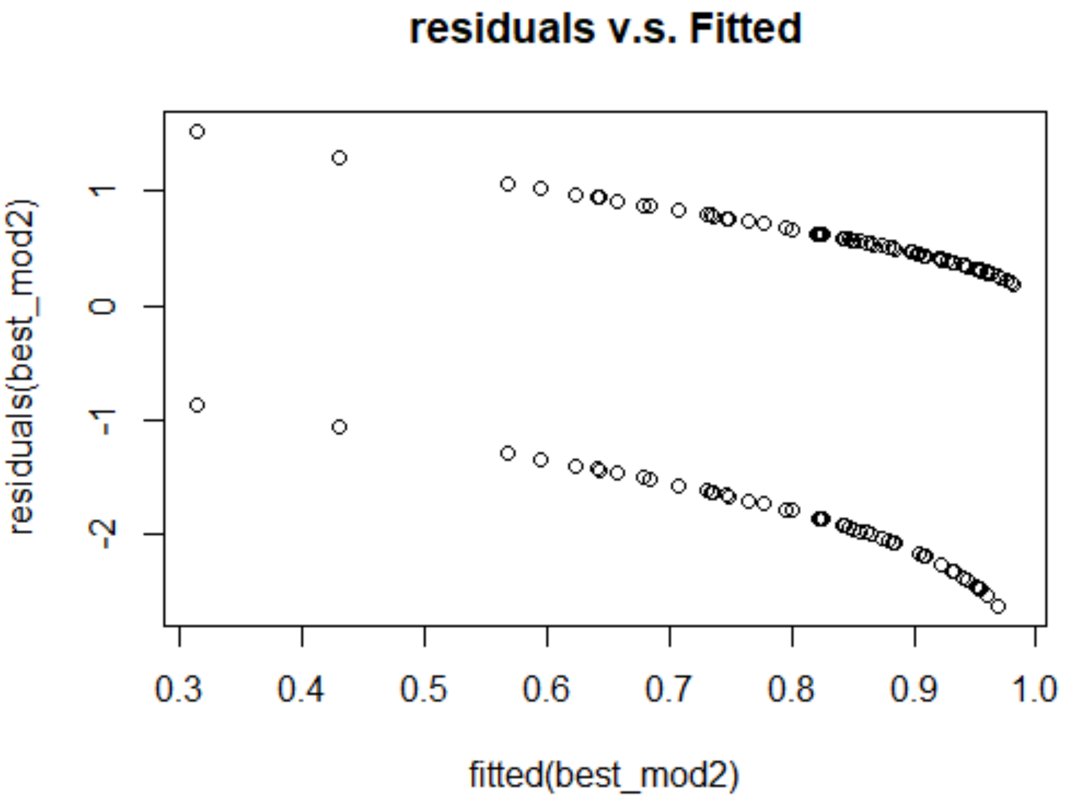

C.Aphasia

Correct ~ Session (1/3) * Aphasia Group + (1|Subject)

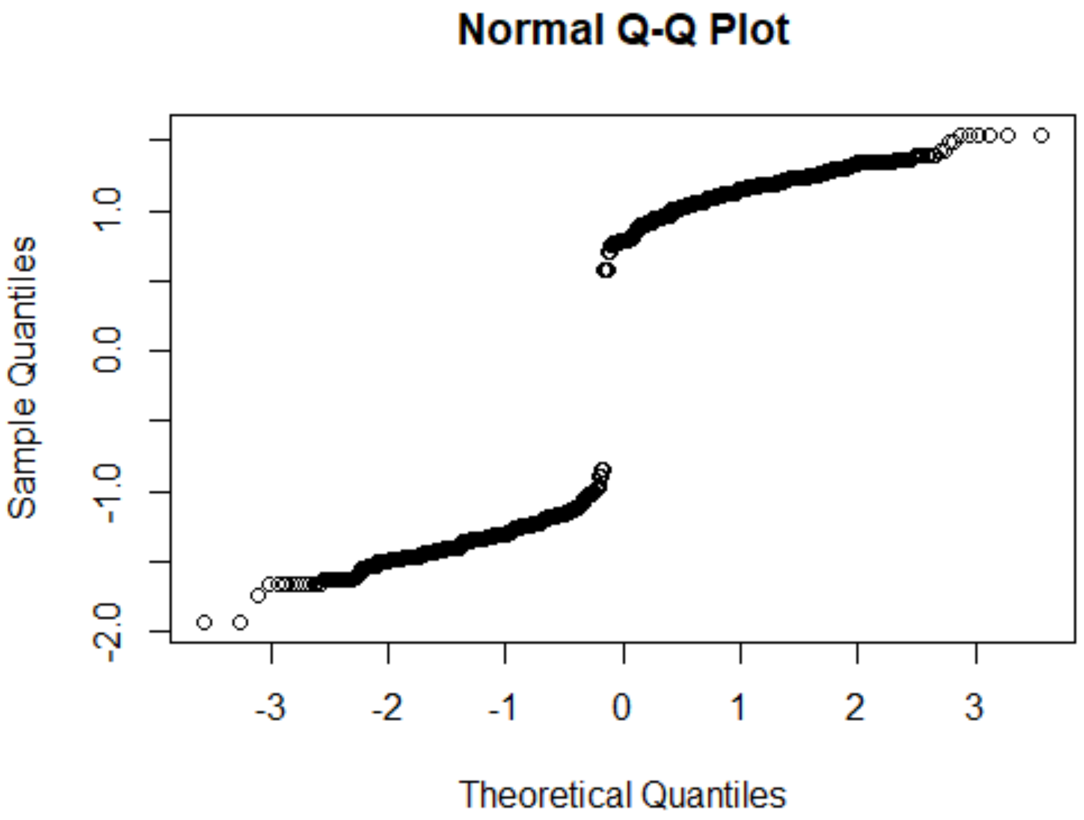

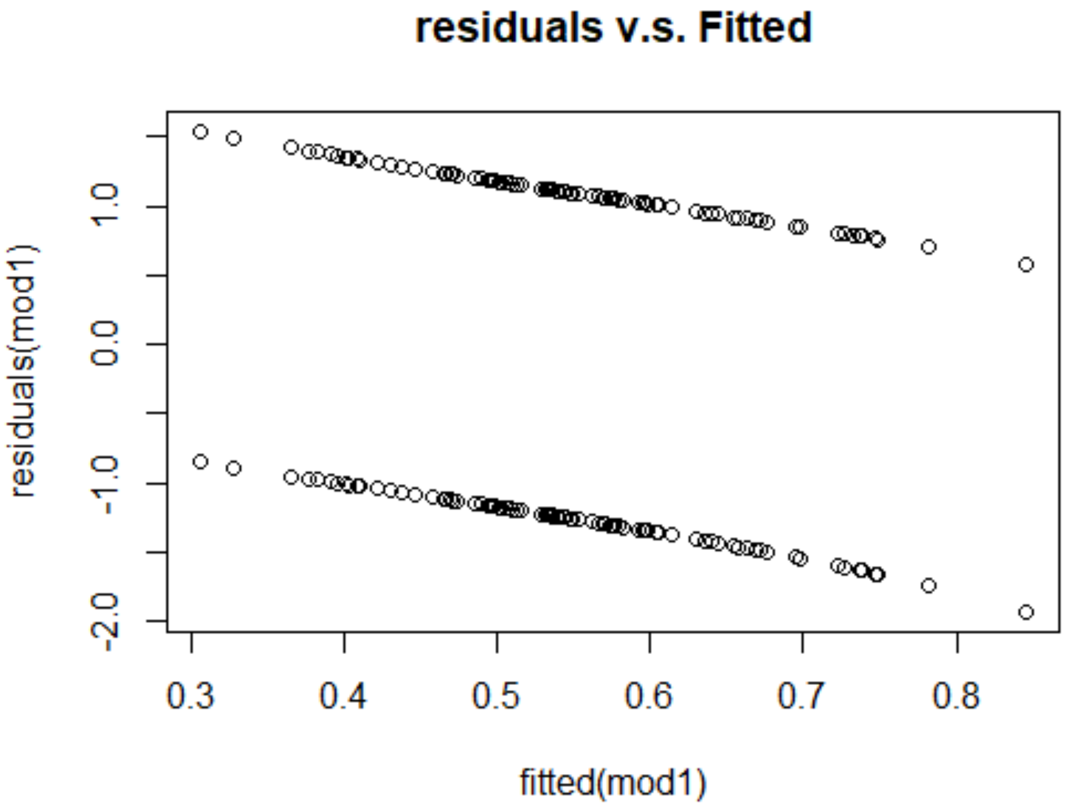

D. ORCP Models

D1: Correct ~ Session (1/3) * Grammar Type * ORCP + (1|Subject)

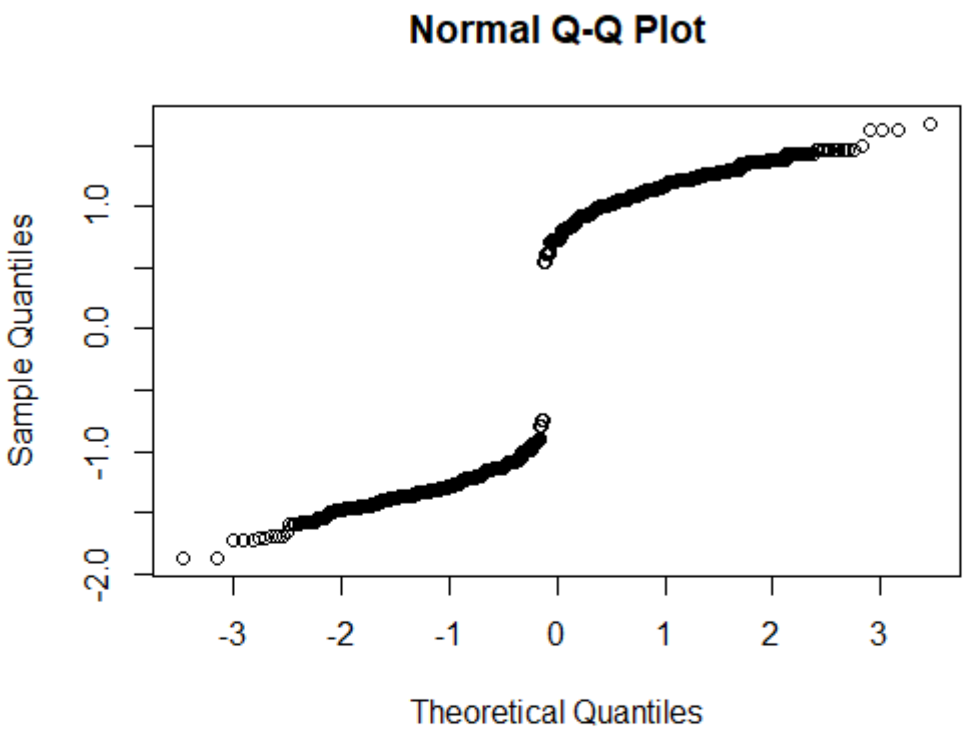

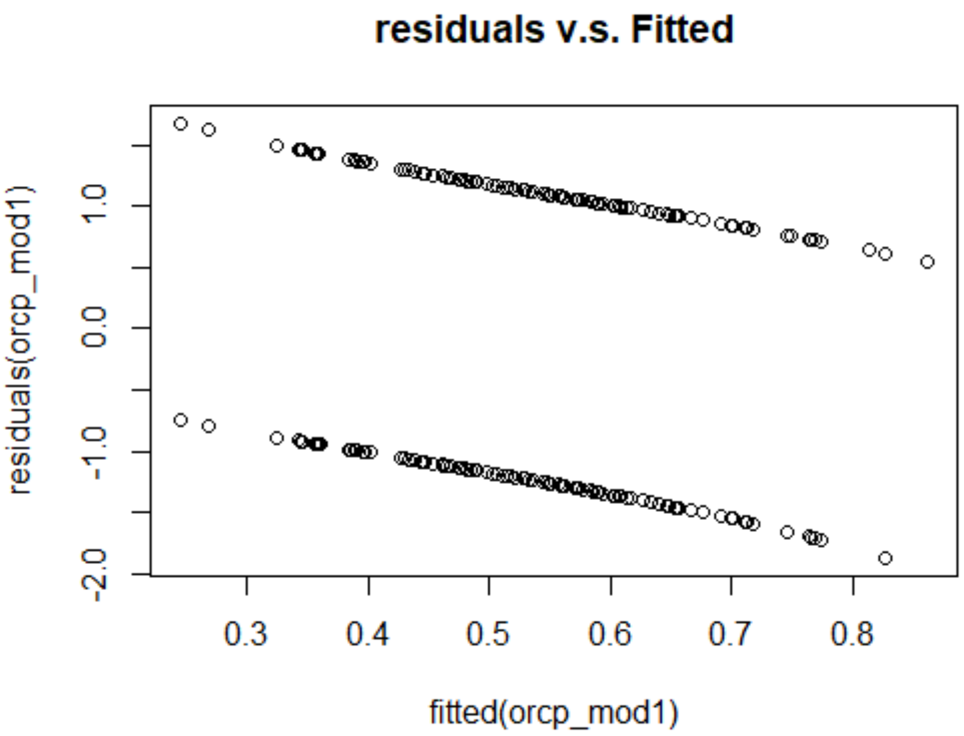

D2: Correct ~ Session (1/3) * Grammatical * ORCP + (1|Subject)

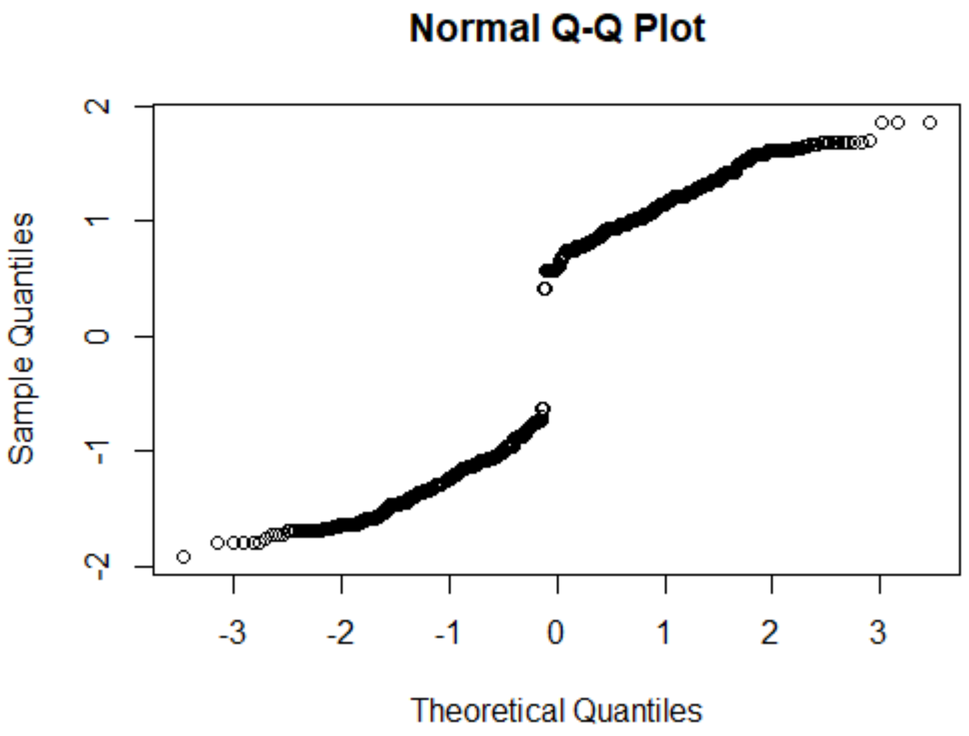

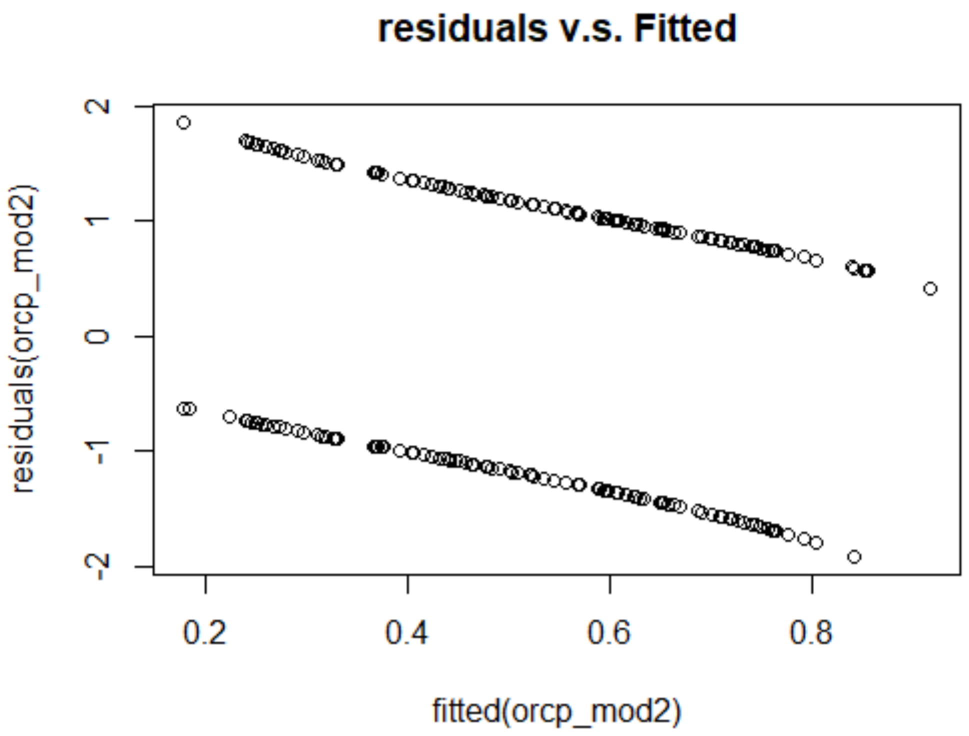

D3: Session 3:

Correct ~ Grammar Type * Grammatical * ORCP + (1|Subject)

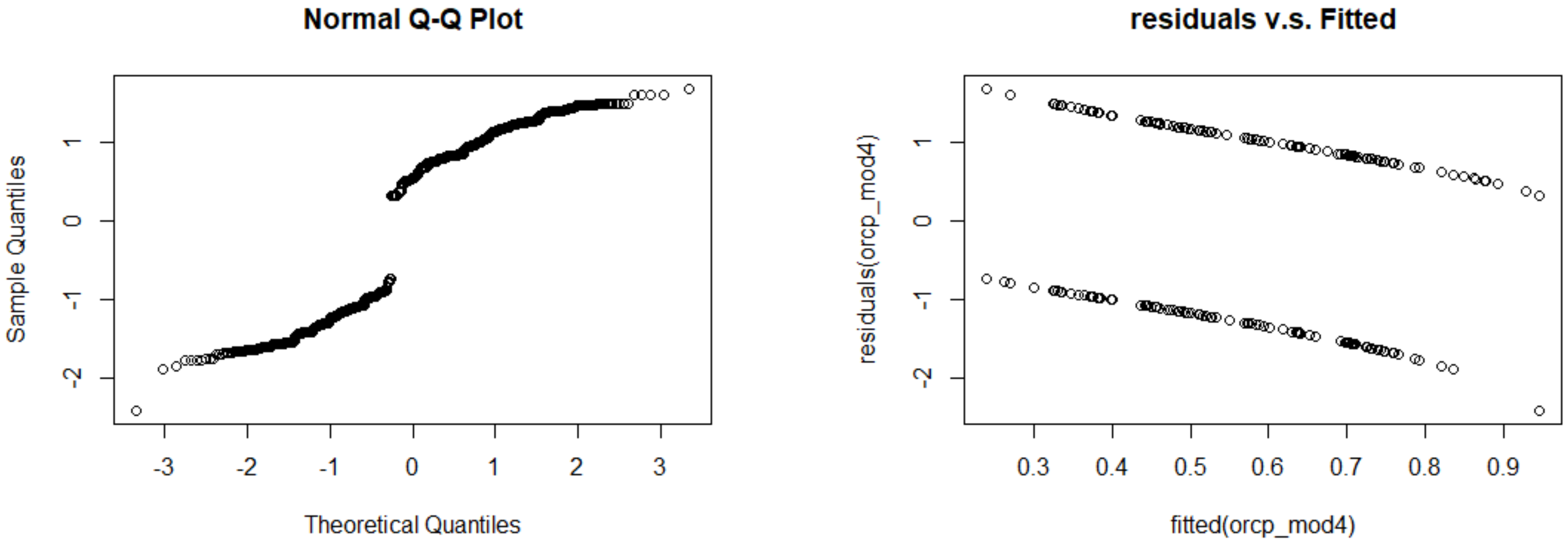

E: Educational Age Models

E1: Correct ~ Session (1/3) * Grammar Type * Ed. Age + (1|Subject)

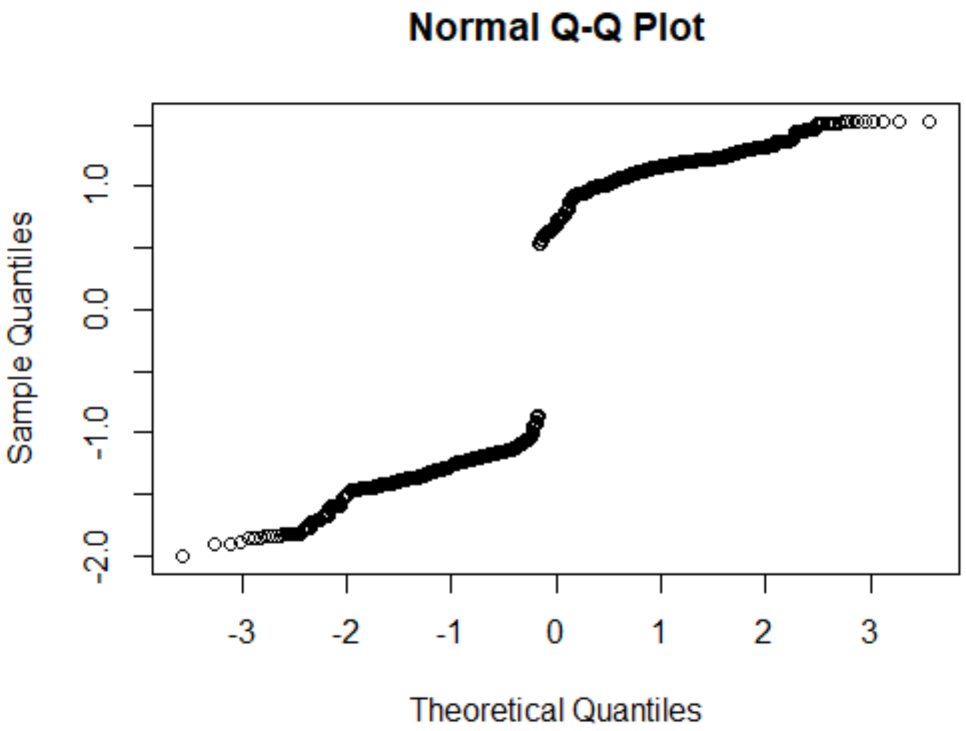

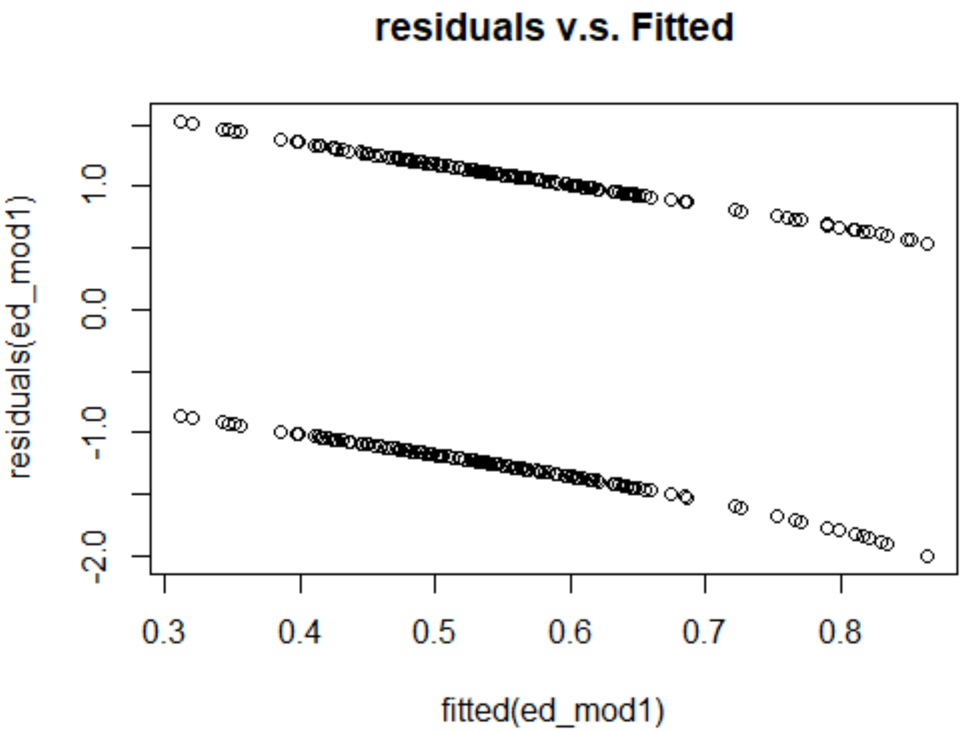

E2: Correct ~ Session (1/3) * Grammatical * Ed. Age + (1|Subject)

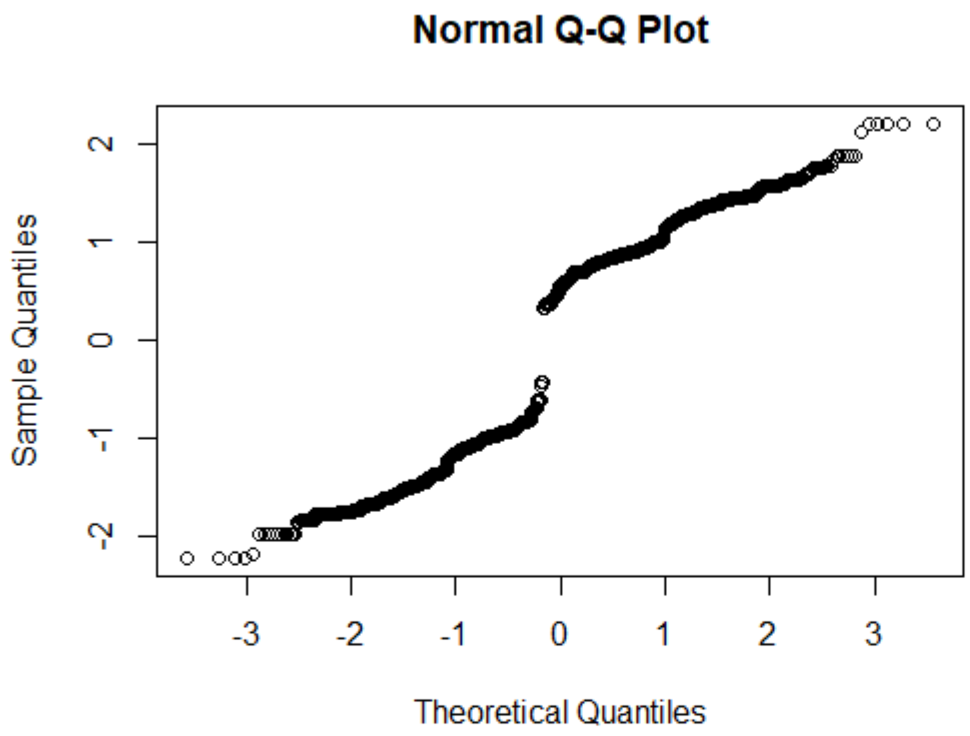

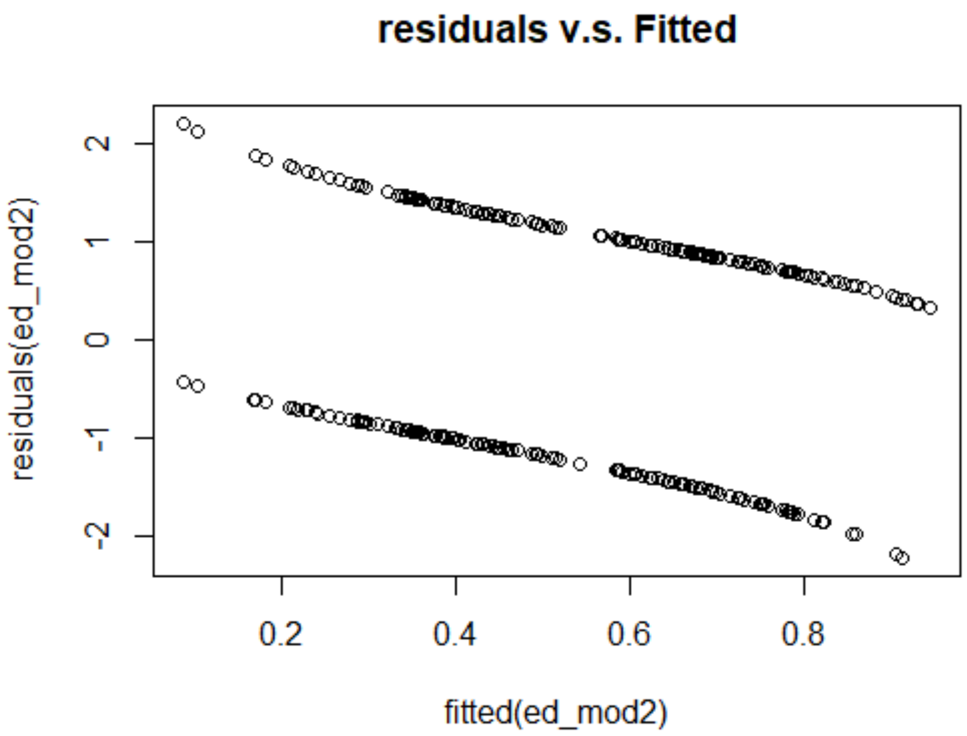

E3: Session 1:

Correct ~ Grammar Type * Grammatical * Ed. Age + (1|Subject)

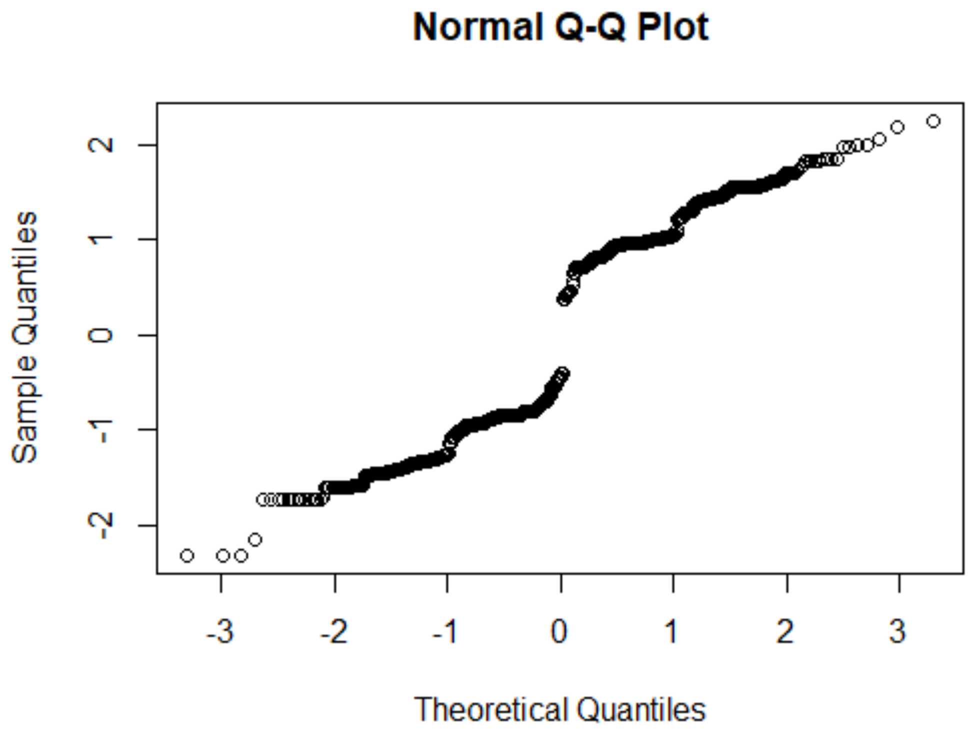

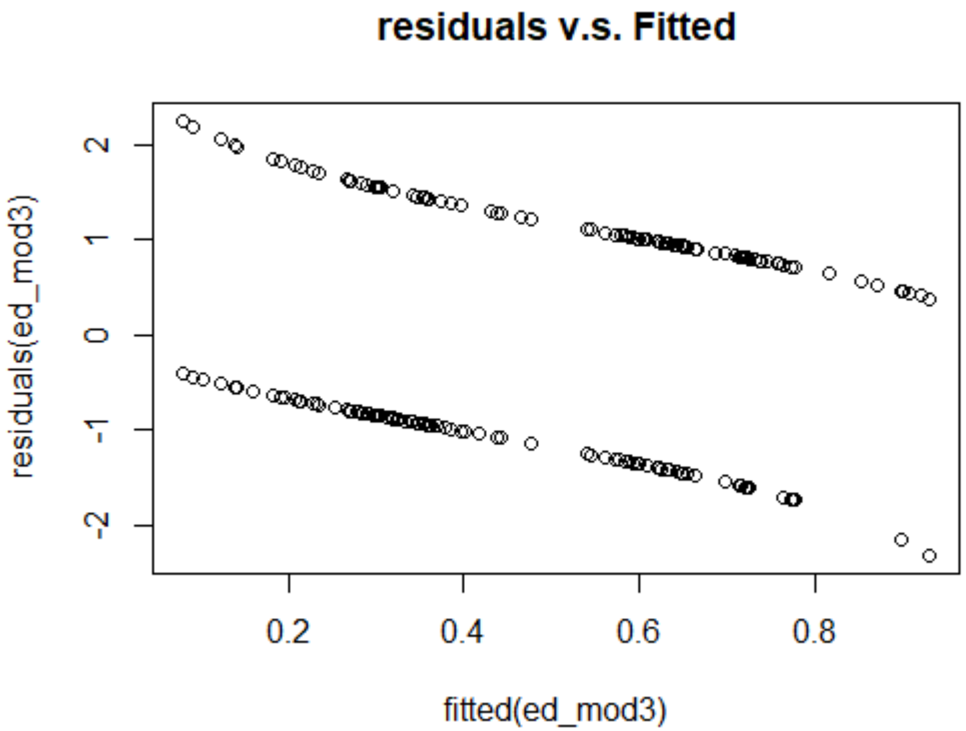

E4: Session 3:

Correct ~ Grammar Type * Grammatical * Ed. Age + (1|Subject)

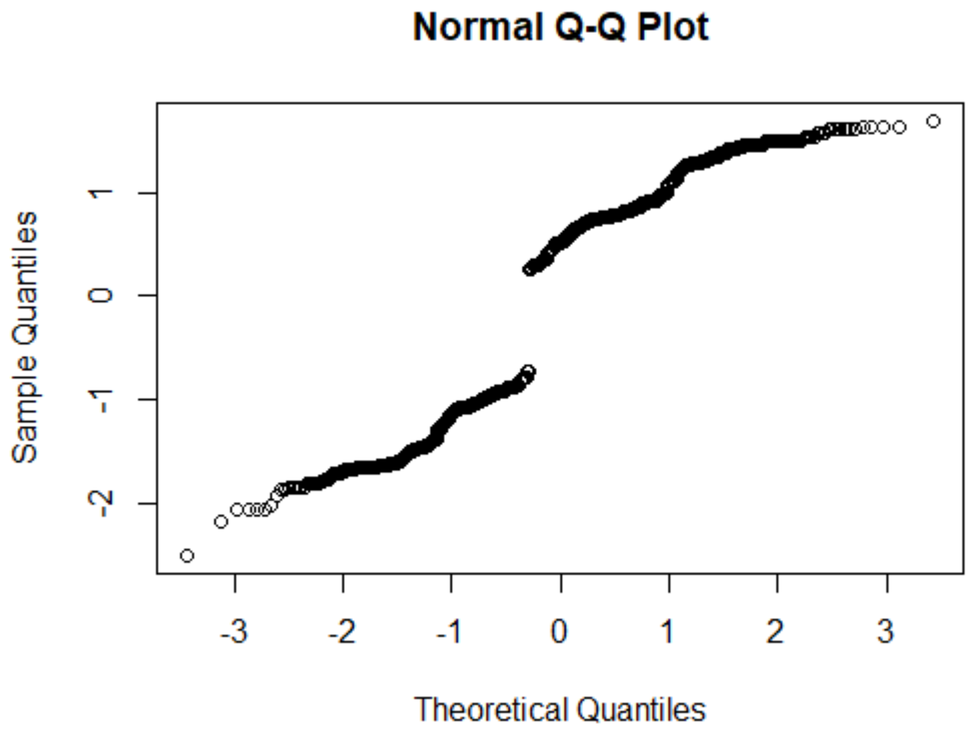

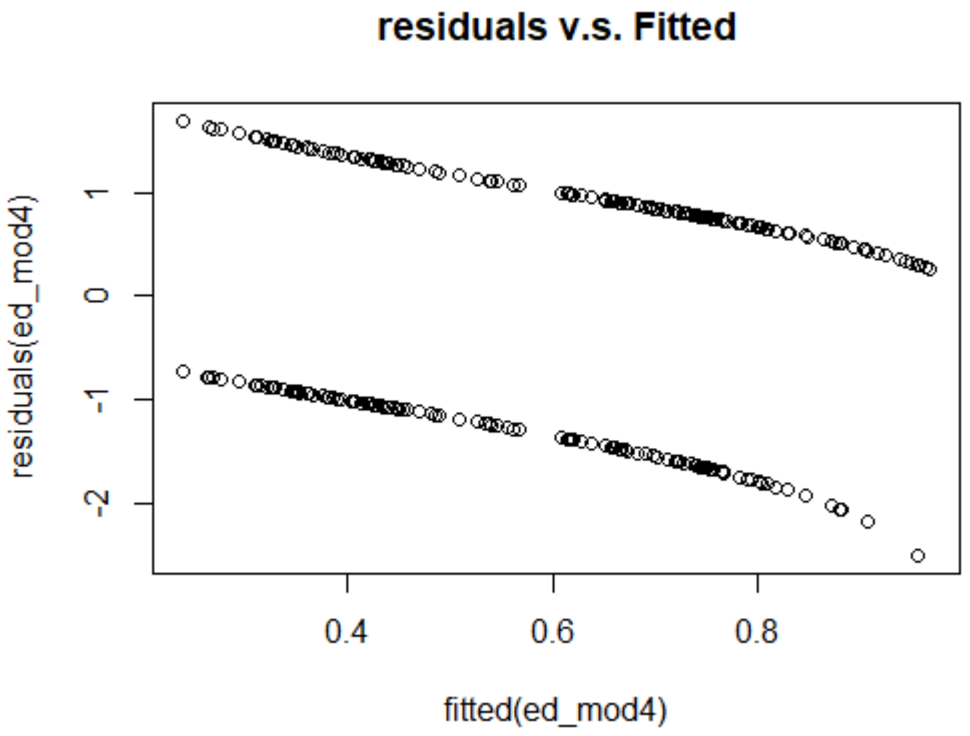

F: Corsi Models

F1: Correct ~ Session (1/3) * Grammar Type * Corsi + (1|Subject)

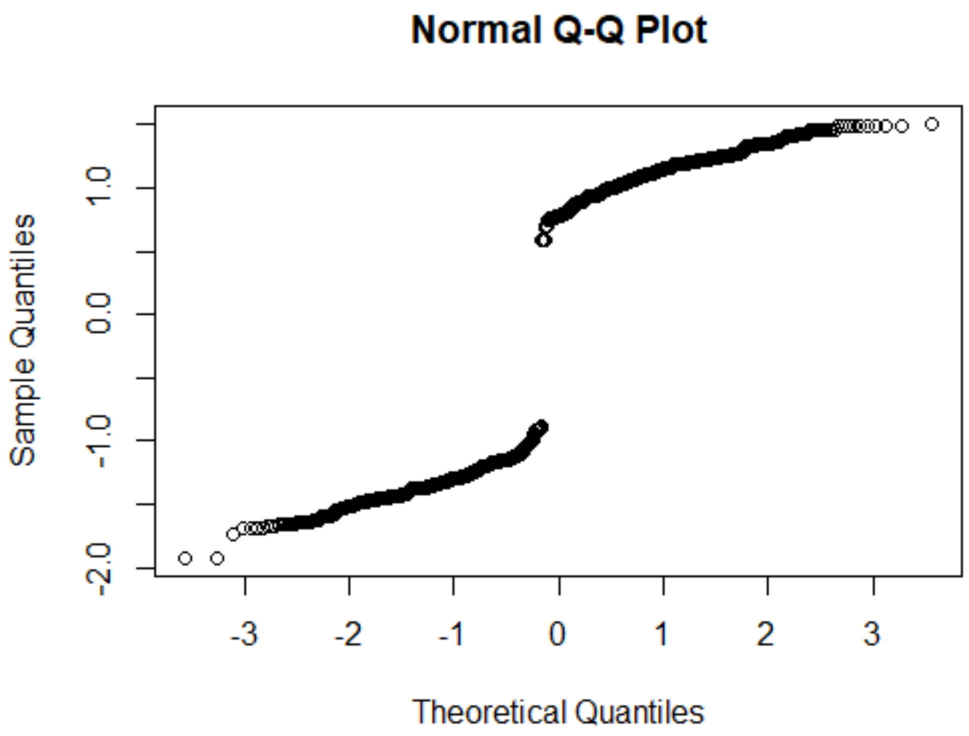

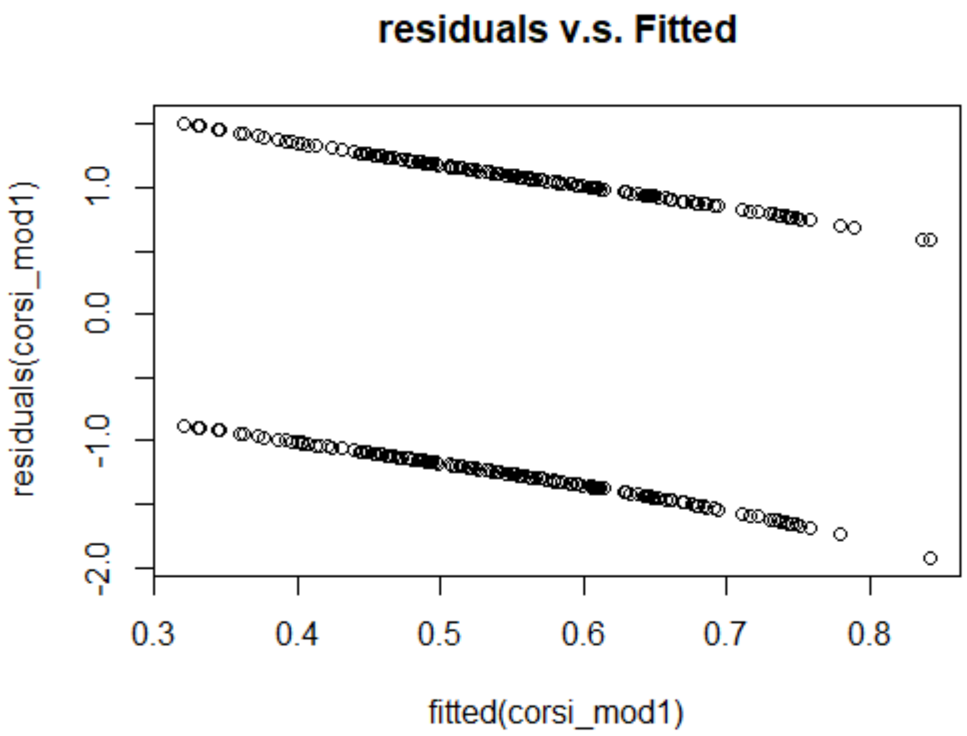

F2: Correct ~ Session (1/3) * Grammatical * Corsi + (1|Subject)

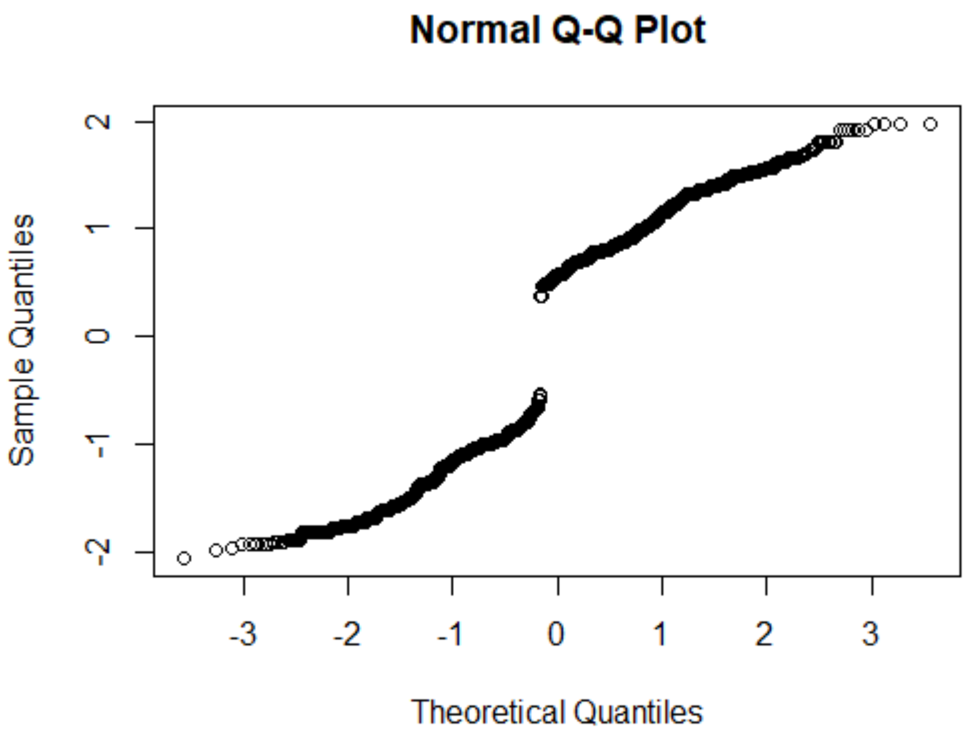

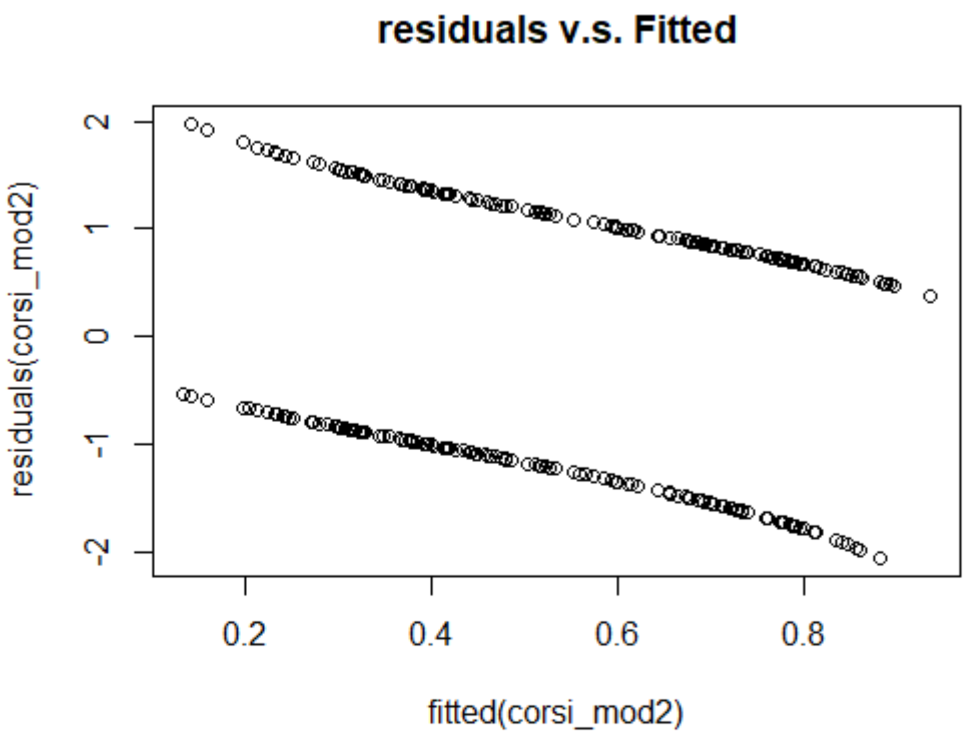

F3: Session 1:

Correct ~ Grammar Type * Grammatical * Corsi + (1|Subject)

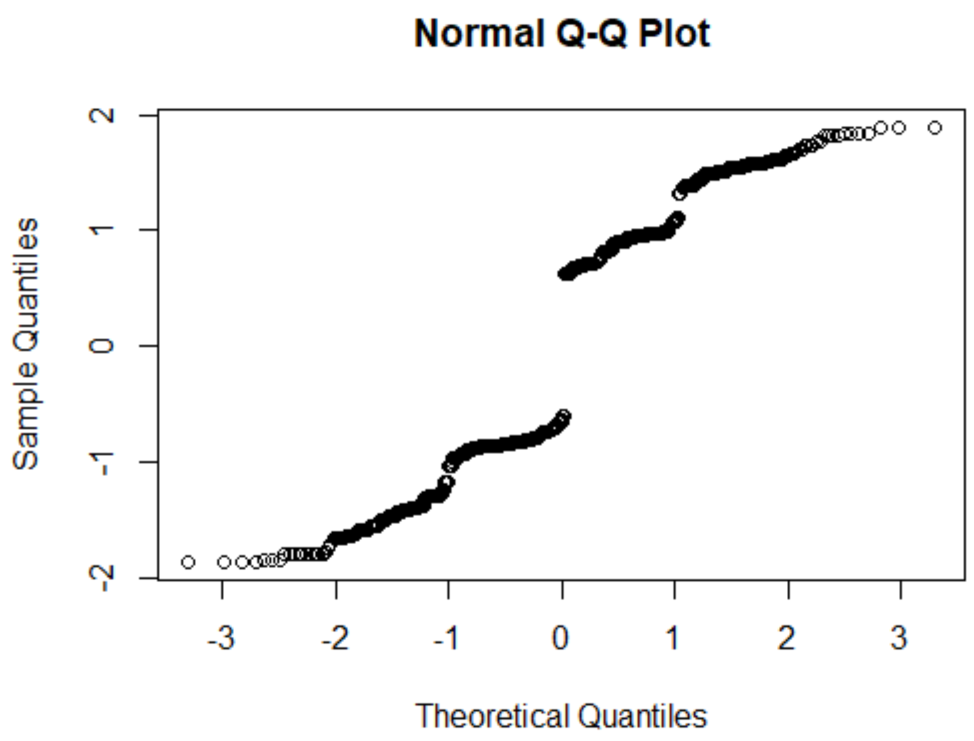

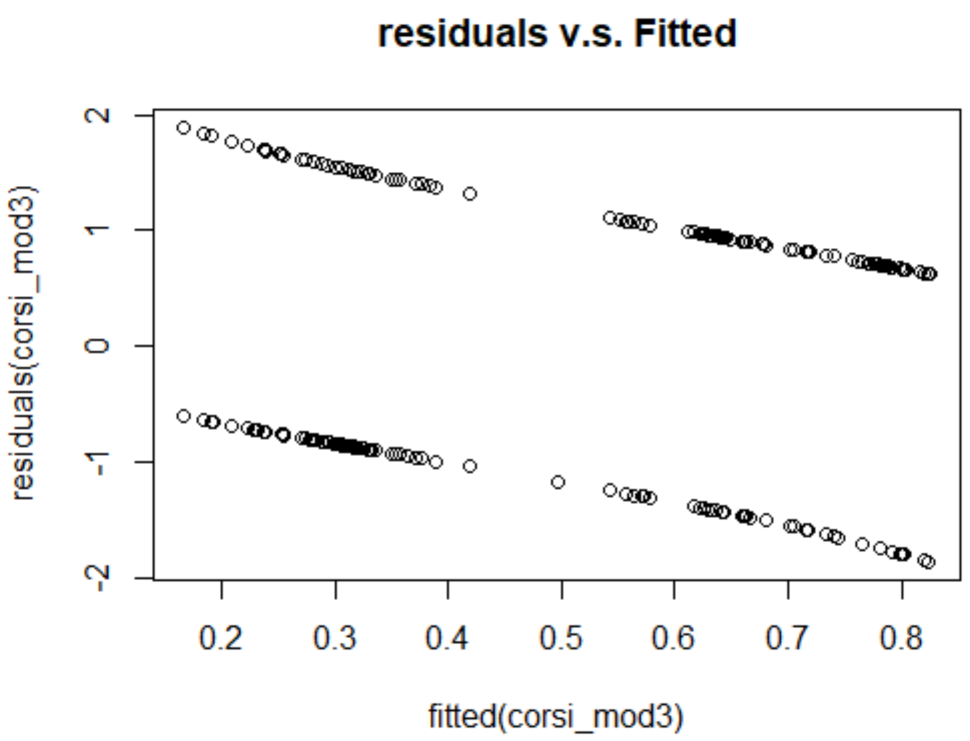

F4: Session 3:

Correct ~ Grammar Type * Grammatical * Ed. Age + (1|Subject)

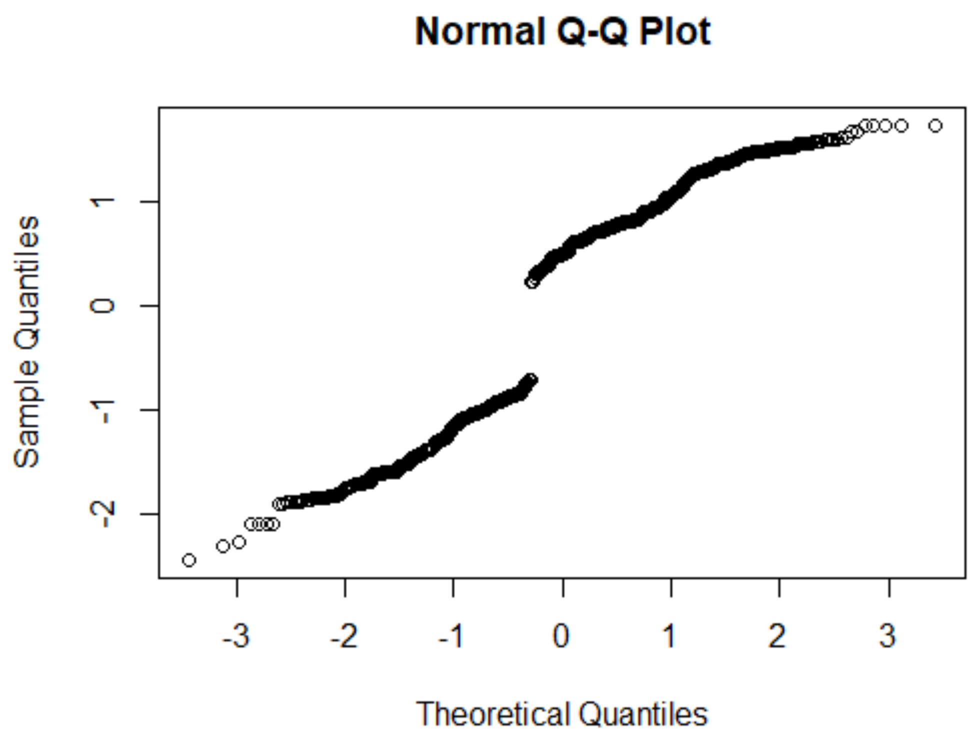

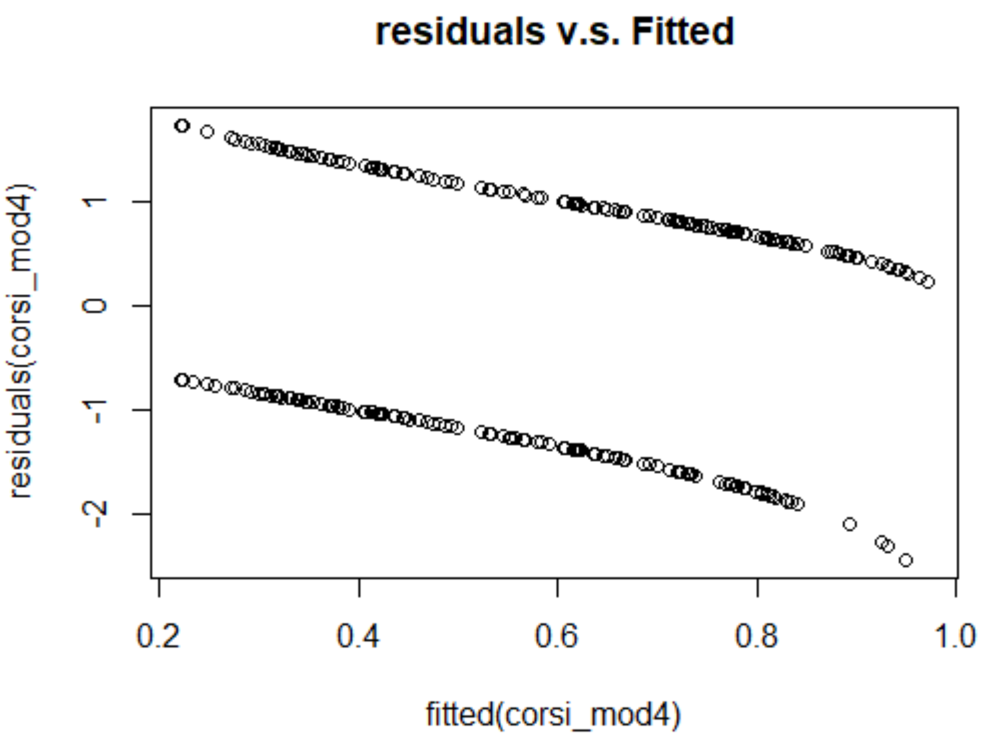

Supplement: Supplementary file 1 [file brainsci-12-00616-s001.zip › Supplementary data analysis.pdf]
