# Supplementary material for: Syntax Acquisition in Healthy Adults and Post-Stroke Individuals: The Intriguing Role of Grammatical Preference, Statistical Learning, and Education
Source: Brain Sci. 2022 May 9;12(5):616. doi: 10.3390/brainsci12050616 (PMC9139563; doi:10.3390/brainsci12050616)
Supplement: Supplementary file 1 [file brainsci-12-00616-s001.zip › Supplementary results.pdf]

Supplementary-results

Results of basic **logistic regression analysis**

a. *Pre-training-sessions*

Basic Models in LHSP

Model Predictor: $R^2 = 0.137$	Estimate (log-odds)	Std. Error	z-Value
Intercept	0,597	0,119	4,999***
Grammaticality (= 0)	-0,762	0,058	-13,201***
Grammar Type (= Embedded)	-0,097	0,057	-1,701
Grammaticality * Grammar	-0,117	0,057	-2,067*

Table S1A. Fixed effects results of the logistic regression analysis conducted on the model including syntax-internal predictors in LHSP_S3

b. *LHSP* ***pre- and post-training session***

Model Predictor: $R^2 = .167$	Estimate (log-odds)	Std. Error	z-Value
Intercept	0,293	0,08	3,67***
Session (= 1)	-0,268	0,047	-5,709***
Grammar Type (= Emb.)	-0,117	0,046	-2,535*
Grammaticality (= 0)	-0,803	0,046	-17,408***
**Session * Grammar**	**-0,022**	**0,046**	**-0,475**
Session * Grammaticality	-0,068	0,046	-1,475
Grammar * Grammaticality	0,041	0,046	0,902
**Session * Grammar * Grammaticality**	**0,148**	**0,046**	**3,232****

Table S1B. Fixed effects results of the logistic regression analysis conducted on the model including syntax-internal predictors in LHSP_S3,S1

c. ***Post-training sessions***

Model Predictor: $R^2 = .081$_S3	Estimate (log-odds)	Std. Error	z-Value

Intercept	2,165	0,169	12,807***
Grammar Type (= Embedded)	-0,089	0,083	-1,084
Grammatical (= no)	-0,564	0,083	-6,784***
Grammar Type * Grammatical	-0,165	0,083	-1,998*

Table S1C. Fixed effects results of the logistic regression analysis conducted on the model including syntax-internal predictors in HP_S3

d. HP pre-and post-training session

Model Predictor: $R^2 = 0.211$_S1&S3			
Intercept	1,198	0,075	15,998***
Session (= 1)	-0,763	0,05	-15,356***
Grammar Type (= Emb.)	-0,218	0,049	-4,408***
Grammaticality (= 0)	-0,516	0,05	-10,427***
Session * Grammar	-0,139	0,049	-2,806**
Session * Grammaticality	0	0,049	-0,003
Grammar * Grammaticality	-0,007	0,049	-0,145
**Session * Grammar * Grammaticality**	**0,144**	**0,049**	**2,913****

Table S1D. Fixed effects results of the logistic regression analysis conducted on the model including syntax-internal predictors in HP_S3&S1

- ***Models including classification accuracy of ungrammatical items, GT, and error type***

$R^2 = 0.055$_LHSP			
Intercept	-1,643	0,786	-2,091*
Grammar Type (= Emb.)	0,704	0,421	1,672
Error Type (= Permutation)	0,802	0,421	1,904
Working Memory (wm)	1,716	0,92	1,865
Grammar Type * Error Type	0,621	0,419	1,483
**Grammar Type * wm**	-1,088	0,494	**-2,2***
Error Type * Working Memory	-0,944	0,493	-1,914

Grammar Type * Error Type * wm	-0,922	0,491	-1,879

R^2 = 0.085_HP			
Intercept	-6,609	5,203	-1,27
**Grammar Type (= Emb.)**	-5,969	2,83	**-2,109***
Error Type (= Permutation)	0,18	2,806	0,064
Working Memory (wm)	8,381	5,321	1,575
Grammar Type * Error Type	-3,328	2,836	-1,173
**Grammar Type * wm**	5,905	2,896	**2,039***
Error Type * Working Memory	-0,121	2,872	-0,042
grammar Type * Error Type * wm	2,935	2,903	1,011
R^2 = 0.066			

Table S2. Fixed effects results of the logistic regression analysis conducted on the model including ungrammatical items, GT, and error type _S3

Generalization HP

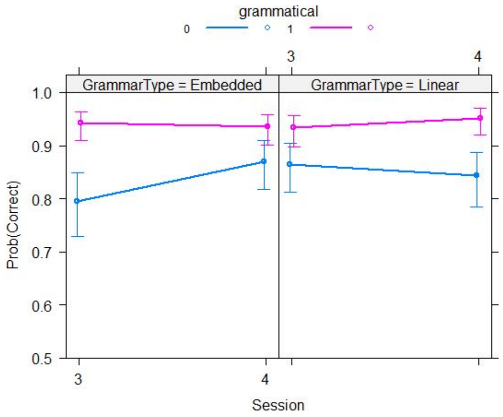

Figure S2: The Effect of session number (S3 and S4) in HP. Grammatical items =1 Ungrammatical items = 0

- *Models including syntactic external predictors*

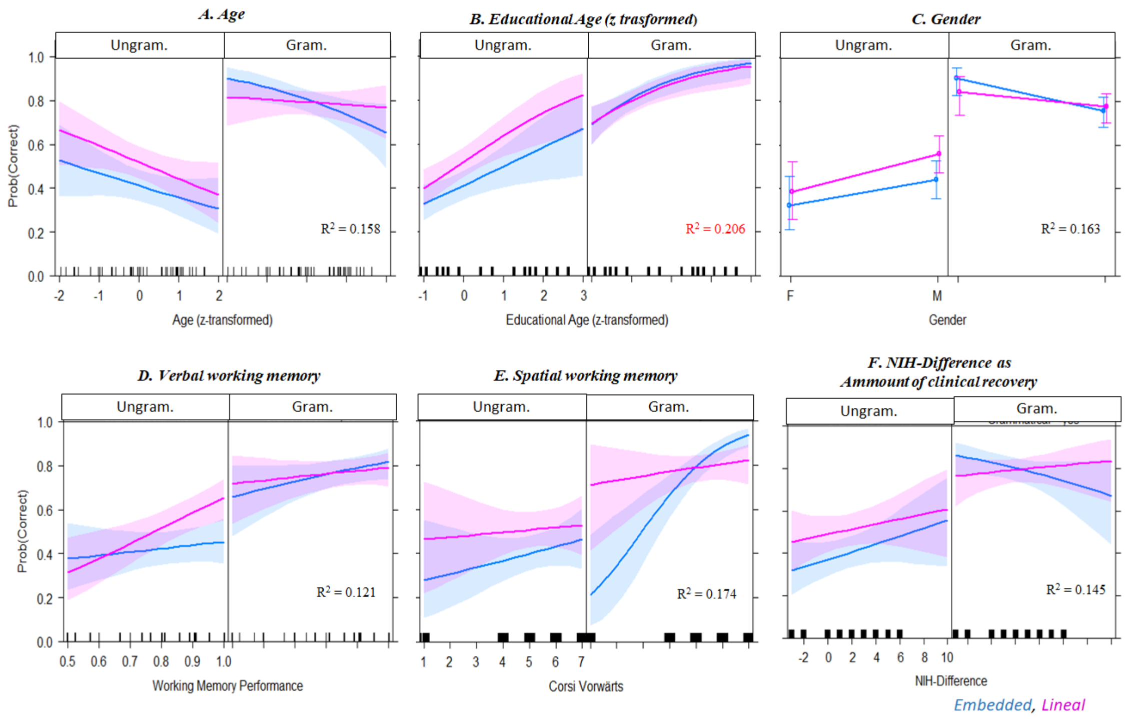

Figure S2: Syntax-external predictors for AG classification accuracy after training in the models including grammaticality and grammar type: Educational age was the model with the strongest R2. Educational age, like age, is a positive predictor independently on grammaticality and grammar type of the items. Gender did not show a significant fixed effect for AG classification accuracy, but a significant interaction with grammaticality: males had a better performance in ungrammatical items, while females in grammatical ones. Concerning the predictor verbal working memory (wm, as measured by the performance on the wm session), there is a significant interaction between wm, grammaticality and grammar type: working memory performance positively interacts with the classification accuracy of ungrammatical sentences of linear but not of embedded ones. Decisive is the contribution of spatial working memory (measured by the Corsi forward) for explaining the grammaticality effect: the effect of corsi forward was stronger not only in embedded than in linear grammar, but also in grammatical rather than ungrammatical conditions. Moreover, there is a significant interaction between grammar type, grammaticality and Corsi forward: the better the performance in Corsi, the better the performance particularly in embedded ungrammatical items and even stronger in grammatical embedded items. F) shows the effect of NIH difference between acute and chronic stroke-phase and AG-learning: the more the recovery amounts the better is the AG classification's accuracy of grammatical items only.

- *Demographic data:*
  a. Age

**A. I. Age_S3**			
$R^2 = .158$			
Intercept	0,62	0,115	5,394***

Grammar Type (= Embedded)	-0,088	0,058	-1,52
**Grammatical (= no)**	**-0,764**	**0,058**	**-13,067*** **
**Age (z-transformed)**	**-0,252**	**0,117**	**-2,155***
**Grammar Type * Grammatical**	**-0,128**	**0,057**	**-2,235***
Grammar Type * Age	-0,063	0,058	-1,097
Grammatical * Age	-0,018	0,058	-0,309
GT * Grammatical * Age	0,1	0,057	1,759
**II. Age_S1**			
Intercept	-0,011	0,082	-0,138
Grammaticality (= 0)	-0,863	0,073	-11,753***
Grammar (= Embedded)	-0,161	0,074	-2,186*
Age	-0,093	0,085	-1,095
Grammaticality * Grammar	0,184	0,073	2,514*
**Grammaticality * Age**	**0,136**	**0,077**	**1,768**
**Grammar * Age**	**0,138**	**0,077**	**1,788**
Grammaticality * Grammar *Age	-0,051	0,077	-0,665

Table S3A. Fixed effects results of the logistic regression analysis conducted on the model including Age

In **S1 and S3** Model including

- *Grammaticality.*:

```
Fixed effects:
                                       Estimate Std. Error z value Pr(>|z|)
(Intercept)                             0.27958    0.07457   3.749 0.000177 ***
Grammatical[S.no]                      -0.77372    0.04452 -17.380  < 2e-16 ***
Session[S.1]                           -0.29693    0.04523  -6.564 5.22e-11 ***
scale_Age                              -0.16697    0.07668  -2.177 0.029456 *
Grammatical[S.no]:Session[S.1]         -0.04473    0.04425  -1.011 0.312085
Grammatical[S.no]:scale_Age             0.05356    0.04510   1.188 0.235005
Session[S.1]:scale_Age                  0.05460    0.04595   1.188 0.234798
Grammatical[S.no]:Session[S.1]:scale_Age  0.07782    0.04493   1.732 0.083252 .
---
Signif. codes:  0 ‘***’ 0.001 ‘**’ 0.01 ‘*’ 0.05 ‘.’ 0.1 ‘ ’ 1
```

- *GT*:

```
Fixed effects:
                                               Estimate Std. Error z value Pr(>|z|)    
(Intercept)                                    0.256308   0.075244   3.406 0.000658 ***
GrammarType[S.Embedded]                       -0.092216   0.042552  -2.167 0.030222 *  
Session[S.1]                                  -0.257347   0.043307  -5.942 2.81e-09 ***
scale_Age                                     -0.196660   0.077604  -2.534 0.011273 *  
GrammarType[S.Embedded]:Session[S.1]          -0.009342   0.042515  -0.220 0.826081    
GrammarType[S.Embedded]:scale_Age              0.042261   0.043195   0.978 0.327899    
Session[S.1]:scale_Age                         0.050291   0.044016   1.143 0.253226    
GrammarType[S.Embedded]:Session[S.1]:scale_Age 0.092577   0.042914   2.157 0.030983 *  
---
Signif. codes:  0 ‘***’ 0.001 ‘**’ 0.01 ‘*’ 0.05 ‘.’ 0.1 ‘ ’ 1
```

b. *Educational Age*

**B. I. educational age_S3**			
R^2 = .206 S3			
Intercept	0,624	0,102	6,1***
Grammar Type (= Embedded)	-0,091	0,059	-1,563
**Grammatical (= no)**	**-0,773**	**0,059**	**-13,02*** **
**Educational Age (z-transformed)**	**0,513**	**0,11**	**4,657*** **
**Grammar Type * Grammatical**	**-0,126**	**0,058**	**-2,16* **
Grammar Type * Edu. Age	-0,013	0,065	-0,193
Grammaticality * Edu. Age	-0,09	0,067	-1,339
Grammar Type * Grammatical * Edu. Age	-0,057	0,065	-0,869
**II. educational age_S1**			
Intercept	.018	.084	.213
Grammaticality (= 0)	-0,892	0,075	-11,88***
Grammar (= Embedded)	-0,19	0,076	-2,519*
Educational Age	-0,03	0,092	-0,32
**Grammaticality * Grammar**	0,171	0,075	2,279*
Grammaticality * Ed. Age	-0,395	0,084	-4,693***
**Grammar * Ed. Age**	-0,182	0,084	-2,157*
Gramm * GT *Ed. Age	0,001	0,084	0,007

Table S3B. Fixed effects results of the logistic regression analysis conducted on the model including Education

c. *Gender*

Gender did not show a significant effect for AG classification accuracy in the post-training session, but there was a significant interaction between gender and grammaticality: males had a better performance in ungrammatical items, while females in grammatical ones. The grammaticality effect remained highly significant as well as the interaction between grammar type and grammaticality as in the model without this external variable. Gender difference was independent on sessions

**C. Gender**			
$R^2 = .163$			
Intercept	0,637	0,14	4,558***
Gender (= female)	0,046	0,139	0,333
Grammar Type (= Embedded)	-0,034	0,07	-0,483
**Grammatical (= no)**	**-0,941**	**0,072**	**-13,106*** **
Gender * Grammar Type	0,112	0,07	1,6
Gender * Grammatical	-0,348	0,072	-4,854***
**Grammar Type * Grammatical**	**-0,153**	**0,07**	**-2,193***
Gender * GT * Grammatical	-0,058	0,07	-0,83

Table S3C. Fixed effects results of the logistic regression analysis conducted on the model including Gender

In **S1 and S3** Model including

Grammaticality:

```
Fixed effects:
                                        Estimate Std. Error z value Pr(>|z|)
(Intercept)                              0.26261    0.09486   2.768  0.00563 **
Grammatical[S.no]                       -0.94413    0.05561 -16.979  < 2e-16 ***
Session[S.1]                            -0.34442    0.05669  -6.075 1.24e-09 ***
Gender[S.F]                             -0.01424    0.09460  -0.151  0.88031
Grammatical[S.no]:Session[S.1]          -0.04378    0.05510  -0.795  0.42687
Grammatical[S.no]:Gender[S.F]           -0.32175    0.05558  -5.789 7.07e-09 ***
Session[S.1]:Gender[S.F]                -0.08270    0.05673  -1.458  0.14491
Grammatical[S.no]:Session[S.1]:Gender[S.F]  0.00280    0.05519   0.051  0.95954
---
Signif. codes:  0 ‘***’ 0.001 ‘**’ 0.01 ‘*’ 0.05 ‘.’ 0.1 ‘ ’ 1
```

GT:

```
Fixed effects:
                                              Estimate Std. Error z value Pr(>|z|)
(Intercept)                                    0.23927    0.08963   2.670  0.00759 **
GrammarType[S.Embedded]                       -0.08158    0.04872  -1.675  0.09401 .
Session[S.1]                                  -0.25599    0.04976  -5.145 2.68e-07 ***
Gender[S.F]                                   -0.02935    0.08940  -0.328  0.74271
GrammarType[S.Embedded]:Session[S.1]          -0.02708    0.04835  -0.560  0.57536
GrammarType[S.Embedded]:Gender[S.F]            0.03104    0.04872   0.637  0.52400
Session[S.1]:Gender[S.F]                      -0.02937    0.04979  -0.590  0.55530
GrammarType[S.Embedded]:Session[S.1]:Gender[S.F] -0.05165    0.04834  -1.069  0.28529
---
Signif. codes:  0 ‘***’ 0.001 ‘**’ 0.01 ‘*’ 0.05 ‘.’ 0.1 ‘ ’ 1
```

In HP: In S3 no effect between gender and grammaticality, but between Gender and GT: In E males are better than females.

```
Fixed effects:
                                   Estimate Std. Error z value Pr(>|z|)
(Intercept)                         2.01641    0.15824  12.743   <2e-16 ***
GrammarType[S.Embedded]            -0.14774    0.07593  -1.946   0.0517 .
Gender[S.female]                   -0.17840    0.15473  -1.153   0.2489
GrammarType[S.Embedded]:Gender[S.female]  0.19081    0.07594   2.513   0.0120 *
---
Signif. codes:  0 ‘***’ 0.001 ‘**’ 0.01 ‘*’ 0.05 ‘.’ 0.1 ‘ ’ 1
```

d. Verbal working memory

D. I. verbal wm LHSP_S3			
$R^2 = 0.121$ (S3)			
Intercept	-0,674	0,635	-1,061
Grammar Type (= Embedded)	0,164	0,314	0,523
**Grammatical (= no)**	**-0,841**	**0,318**	**-2,649****
Working Memory	1,491	0,744	2,004*
Grammar Type * Grammatical	0,524	0,311	1,684
Grammar Type * Working Memory	-0,326	0,369	-0,884
Grammatical * Working Memory	0,247	0,372	0,662
Grammar Type * Grammatical * Working Memory	-0,77	0,365	-2,107*
II. R = .12 in LHSP_S1			
Intercept	0,231	0,462	0,5
Grammar Type (= Embedded)	0,509	0,413	1,231
**Grammatical (= no)**	**-0,8**	**0,407**	**-1,964***
Working Memory	-0,307	0,539	-0,569
**Grammar Type * Grammatical**	0,291	0,407	0,714
Grammar Type * Working Memory	-0,792	0,481	-1,645

Grammatical * Working Memory	0,157	0,475	0,33
Grammar Type * Grammatical * Working Memory	-0,123	0,474	-0,26
HP			
III. R = .095 _ S3			
Intercept	2,175	0,168	12,94***
Grammar Type (= Embedded)	-0,09	0,083	-1,077
**Grammatical (= no)**	-0,562	0,084	-6,697***
Working Memory	0,137	0,162	0,844
**Grammar Type * Grammatical**	-0,153	0,083	-1,83
Grammar Type * Working Memory	-0,006	0,08	-0,077
Grammatical * Working Memory	0,11	0,081	1,36
Grammar Type * Grammatical * Working Memory	0,194	0,08	2,419*

Table S3D. Fixed effects results of the logistic regression analysis conducted on the model including Verbal working memory

In **S1 and S3** Model including

Grammaticality:

```
Fixed effects:
                                                  Estimate Std. Error z value Pr(>|z|)
(Intercept)                                       0.249957   0.082433   3.032  0.00243 **
Grammatical[S.no]                                -0.612614   0.045549 -13.449  < 2e-16 ***
Session[S.1]                                     -0.294350   0.046441  -6.338 2.33e-10 ***
scale(WorkingMemory)                              0.090620   0.081402   1.113  0.26561
Grammatical[S.no]:Session[S.1]                   -0.004471   0.045306  -0.099  0.92139
Grammatical[S.no]:scale(WorkingMemory)            0.021314   0.045286   0.471  0.63788
Session[S.1]:scale(WorkingMemory)                -0.138123   0.045553  -3.032  0.00243 **
Grammatical[S.no]:Session[S.1]:scale(WorkingMemory) -0.012241   0.044847  -0.273  0.78488
---
Signif. codes:  0 ‘***’ 0.001 ‘**’ 0.01 ‘*’ 0.05 ‘.’ 0.1 ‘ ’ 1
```

Grammar type:

```
Fixed effects:
                                                     Estimate Std. Error z value Pr(>|z|)
(Intercept)                                          0.231651   0.086270   2.685  0.00725 **
GrammarType[S.Embedded]                             -0.104437   0.045000  -2.321  0.02030 *
Session[S.1]                                        -0.252706   0.045682  -5.532 3.17e-08 ***
scale(WorkingMemory)                                 0.160624   0.085597   1.877  0.06058 .
GrammarType[S.Embedded]:Session[S.1]                 0.005642   0.044851   0.126  0.89990
GrammarType[S.Embedded]:scale(WorkingMemory)        -0.125062   0.045182  -2.768  0.00564 **
Session[S.1]:scale(WorkingMemory)                   -0.103479   0.045371  -2.281  0.02256 *
GrammarType[S.Embedded]:Session[S.1]:scale(WorkingMemory) -0.050734   0.044921  -1.129  0.25873
---
Signif. codes:  0 ‘***’ 0.001 ‘**’ 0.01 ‘*’ 0.05 ‘.’ 0.1 ‘ ’ 1
```

e. *Spatial working memory*

E. spatial wm (forward) I_S1	LHSP		
R^2 = 0.193 (S3)			
Intercept	-0,612	0,58	-1,055
**Grammar Type (= Embedded)**	**-0,909**	**0,269**	**-3,375*****
Corsi (forward)	0,236	0,11	2,149*
**Grammatical (= no)**	**-0,02**	**0,269**	**-0,076**
Grammar Type * Corsi	0,162	0,052	3,11**
Grammar Type * Grammatical	0,47	0,267	1,758
**Corsi * Grammatical**	**-0,148**	**0,052**	**-2,847****
GT * Corsi * Grammatical	-0,118	0,052	-2,284*
II. R= .193 _**S1**			
Intercept	-0,078	0,358	-0,218
Grammar Type (= Embedded)	0,223	0,312	0,717
Corsi (vorwärts)	0,014	0,068	0,199
Grammatical (= no)	-0,005	0,31	-0,016
Grammar Type * Corsi	-0,076	0,06	-1,276
Grammar Type * Grammatical	0,694	0,31	2,24*
**Corsi (vorwärts) * Grammatical**	**-0,168**	**0,059**	**-2,841****
**GT * Corsi* Grammatical**	**-0,099**	**0,059**	**-1,672**

Table S3E. Fixed effects results of the logistic regression analysis conducted on the model including Spatial working memory

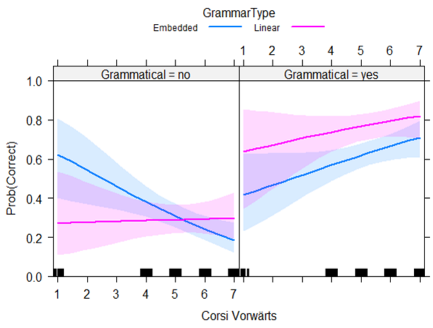

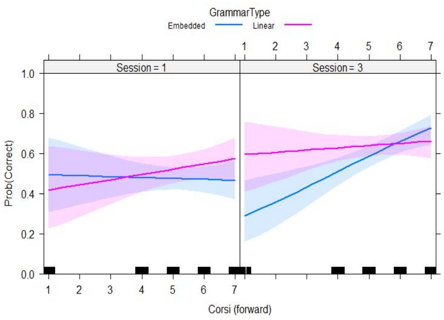

Figure S4: A. Corsif*GT*grammaticality_S1 B. Corsif*GT*Session 1,3

Table 3E-III/IV. **Fixed effects results of the logistic regression analysis conducted on the model including Spatial working memory**

Corsi (backward)			
III) R^2 = .183 in **S3**			
Intercept	0,094	0,434	0,217
Grammar Type (= Embedded)	-0,569	0,2	-2,851**
Corsi (rückwärts)	0,104	0,087	1,195
**Grammatical (= no)**	**-0,219**	**0,198**	**-1,105**
**Grammar Type * Corsi**	**0,1**	**0,041**	**2,475***
**Grammar Type * Grammatical**	**0,102**	**0,197**	**0,516**
**Corsi * Grammatical**	**-0,116**	**0,04**	**-2,861****
GT * Corsi * Grammatical	-0,046	0,04	-1,158
IV) R = .183 In S1			
Intercept	0,045	0,298	0,152
Grammar Type (= Embedded)	0,022	0,268	0,084
Corsi (rückwärts)	-0,01	0,058	-0,174
Grammatical (= no)	-0,977	0,266	-3,677***
Grammar Type * Corsi (rückwärts)	-0,039	0,052	-0,744
Grammar Type * Grammatical	0,72	0,266	2,707**
Corsi (vorwärts) * Grammatical	0,023	0,052	0,444
**GT * Corsi* Grammatical**	**-0,107**	**0,052**	**-2,079***

Table S3E. Fixed effects results of the logistic regression analysis conducted on the model including Corsi

f. Stroke's severity

Lesion size was not a predictor for AG accuracy (E= .081, SE= .135, z= .601), as well as NIHSS_ at testing (E= -.002., SE= .084 , z= -.031).

g. Difference between NIHSS at testing and NIHSS_admission (acute stroke phase)

Table 4F: There was a significant interaction between NIHSS difference and grammaticality: the more the recovery amount the better was the AG classification's accuracy of grammatical items only. The grammaticality effect remained highly significant as well as the interaction between grammar type and grammaticality as in the model without this external variable.

Predictors	Estimate (log-odds)	Std. Error	z-Value
F. NIHSS Difference			
R^2 = .145_S3			
Intercept	0,566	0,148	3,819***
**Grammatical (= no)**	**-0,854**	**0,072**	**-11,912*** **
Grammar Type (= Embedded)	-0,05	0,071	-0,707
NIH-difference	0,018	0,045	0,402
**Grammatical * Grammar Type**	**-0,195**	**0,07**	**-2,782** **
Grammatical * NIH-difference	0,044	0,021	2,097*
Grammar Type * NIH-difference	-0,023	0,021	-1,127
GT * Grammatical * NIH-differnce	0,037	0,021	1,804

Table S3F Effect of stroke recovery, measured as the difference between NIHSS in the chronic phase versus the NIHSS at discharge, and AG classification accuracy after the training

Including S1 and S3 and

- *Grammaticality* (R =.164):

```
Fixed effects:
                                      Estimate Std. Error z value Pr(>|z|)
(Intercept)                           0.207046   0.097265   2.129   0.0333 *
Grammatical[S.no]                    -0.836052   0.054549 -15.327  < 2e-16 ***
Session[S.1]                         -0.327341   0.055443  -5.904 3.55e-09 ***
NIH_dif                               0.031946   0.029842   1.070   0.2844
Grammatical[S.no]:Session[S.1]       -0.020019   0.054171  -0.370   0.7117
Grammatical[S.no]:NIH_dif             0.032304   0.015857   2.037   0.0416 *
Session[S.1]:NIH_dif                  0.017545   0.016451   1.066   0.2862
Grammatical[S.no]:Session[S.1]:NIH_dif -0.008817 0.015821  -0.557   0.5773
---
Signif. codes:  0 ‘***’ 0.001 ‘**’ 0.01 ‘*’ 0.05 ‘.’ 0.1 ‘ ’ 1
```

- *GT*:

```
Fixed effects:
                                          Estimate Std. Error z value Pr(>|z|)
(Intercept)                                0.18473    0.09872   1.871   0.0613 .
GrammarType[S.Embedded]                   -0.12015    0.05122  -2.346   0.0190 *
Session[S.1]                              -0.26725    0.05208  -5.132 2.87e-07 ***
NIH_dif                                    0.03604    0.03056   1.179   0.2382
GrammarType[S.Embedded]:Session[S.1]      -0.05996    0.05114  -1.173   0.2410
GrammarType[S.Embedded]:NIH_dif            0.01191    0.01524   0.782   0.4344
Session[S.1]:NIH_dif                       0.01272    0.01581   0.805   0.4211
GrammarType[S.Embedded]:Session[S.1]:NIH_dif 0.02898  0.01521   1.906   0.0567 .
---
Signif. codes:  0 ‘***’ 0.001 ‘**’ 0.01 ‘*’ 0.05 ‘.’ 0.1 ‘ ’ 1
```

h. Patients' language competence

- *Token Test*

TT performance in the chronic phase was not a predictor for AG Learning (E= .012, SE= .019. z= .612).

- *Aphasia on AAT*

Predictor	Estimate (log-odds)	Std. Error	z-Value
G. Aphasia (AAT)			
Aphasia and S3 and			
**I) GT: R^2 = .006**			
Intercept	0,509	0,118	4,298***
**Grammar Type** (= Embedded)	-0,085	0,054	-1,565
Group (= chronic Aphasia)	-0,173	0,175	-0,99
Group (= no Aphasia)	0,042	0,169	0,25
GT * Group (= chronic A.)	0,007	0,081	0,081
Grammar Type * Group (= no A.)	0,037	0,077	0,48
**2) Grammaticality: R^2 = .139**			
Intercept	0,555	0,117	4,759***
**Grammaticality** (= no)	-0,778	0,059	-13,21***
Group (= chronic Aphasia)	-0,17	0,172	-0,988
Group (= no Aphasia)	-0,006	0,167	-0,037
Grammatical * Group (= chronic A.)	0,006	0,087	0,07
Grammatical * Group (= no A.)	-0,134	0,085	-1,586
Aphasia and S1 and			
**3) GT : R^2 = 0.011**			
Intercept	-0,006	0,078	-0,082
Group (= chronic Aphasia)	0,111	0,116	0,961

Group (= no Aphasia)	-0,061	0,111	-0,546
**Grammar Type** (= Embedded)	-0,097	0,068	-1,411
Group (= chronic) * Grammar Type	0,117	0,104	1,125
Group (= no) * Grammar Type	0,028	0,098	0,289
4) Grammaticalty: $\mathbf{R^2}$ = .173			
Intercept	-0,035	0,079	-0,446
Group (= chronic Aphasia)	0,158	0,117	1,357
Group (= no Aphasia)	-0,06	0,116	-0,52
Grammatical (= no)	-0,847	0,072	-11,779***
Group (= chronic) * Grammatical	0,005	0,107	0,045
Group (= no) * Grammatical	**-0,235**	**0,107**	**-2,206***

Post-hoc: yes = grammatical, no = ungrammatical

**contrast**	**estimate**	**SE**	**df**	**z.ratio**	**p.value**
no aphasia - yes aphasia	-168.485.642	0.27323515	Inf	-616.632.375	**0.0000000105**
**no *aphasia* - no *no_aphasia***	0.45849179	0.28595267	Inf	160.338.348	0.5962861
no aphasia - yes no_aphasia	-17.060.852	0.28055859	Inf	-608.103.006	0.0000000179
no aphasia - no recovery	0.0303483	0.24561328	Inf	0.1235613	0.999996
no aphasia - yes recovery	-120.328.272	0.24417124	Inf	-4.928.028	0.0000123
yes aphasia - no no_aphasia	214.334.821	0.29085372	Inf	736.916.213	0.00000000000262
**yes aphasia - yes no_aphasia**	-0.02122878	0.28509491	Inf	-0.07446214	0.99999968
yes aphasia - no recovery	171.520.471	0.25104148	Inf	683.235.582	0.000000000125
**yes aphasia - yes recovery**	0.48157369	0.24935216	Inf	193.129.945	0.38283923
no no_aphasia - yes no_aphasia	-216.457.699	0.27308921	Inf	-792.626.325	0.000000000000011[illegible]
**no no_aphasia - no recovery**	-0.4281435	0.25100071	Inf	-170.574.619	0.52788952
no no_aphasia - yes recovery	-166.177.452	0.24954652	Inf	-66.591.774	0.000000000413
yes no_aphasia - no recovery	173.643.349	0.24450446	Inf	710.184.799	0.0000000000185
**yes no_aphasia - yes recovery**	0.50280247	0.24274339	Inf	207.133.335	0.30237917
no recovery - yes recovery	-123.363.102	0.19091038	Inf	-646.183.318	0.00000000155

Table S3G. Fixed effects results of the logistic regression analysis conducted on the model including aphasia

- *Object Relative Clause Production (ORCP)*

The logistic regression shows no relation between ORCP Accuracy and chronic aphasia (E= -1.841, SE= 1.278 ,z= -1.158).

**R^2 S3 = .**			
Intercept	**-.090**	**.373**	**.808**
ORCP	.803	.452	1.778
**Grammatical** (= 0)	-.152	.159	-.954
GT	-.196	.161	-1.125
**ORCP * Grammatical**	**-.499**	**.197**	**-2.535***
ORCP* GT	.064	.198	.323
GT*grammaticality	.056	.159	.352
ORCP *GT*grammaticality	-.186	.196	

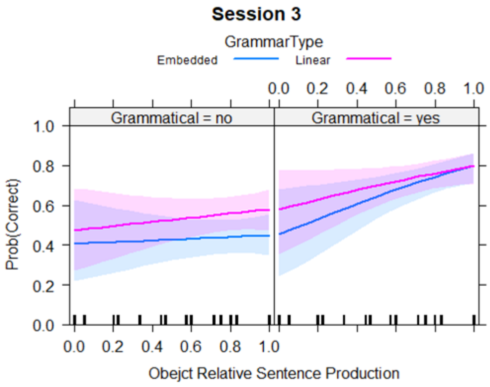

Figure S4: ORCP*GT*grammaticality in S3

In S1 ORCP was not a significant predictor (in this model only grammaticality and the interaction grammaticality * GT was significant)

```
Fixed effects:
                                                   Estimate Std. Error z value Pr(>|z|)
(Intercept)                                         -0.3173     0.2426  -1.308   0.1909
GrammarType[S.Embedded]                             -0.4224     0.2270  -1.861   0.0628 .
Grammatical[S.no]                                   -0.8953     0.2260  -3.962 7.44e-05 ***
ProdAccuracy                                         0.2629     0.2901   0.906   0.3648
GrammarType[S.Embedded]:Grammatical[S.no]            0.5531     0.2260   2.447   0.0144 *
GrammarType[S.Embedded]:ProdAccuracy                 0.3743     0.2736   1.368   0.1713
Grammatical[S.no]:ProdAccuracy                       0.1705     0.2712   0.629   0.5296
GrammarType[S.Embedded]:Grammatical[S.no]:ProdAccuracy -0.2776  0.2713  -1.023   0.3061
---
Signif. codes:  0 ‘***’ 0.001 ‘**’ 0.01 ‘*’ 0.05 ‘.’ 0.1 ‘ ’ 1
```

Generalisation (S3 and S4):

Table S4: Fixed effects results of the logistic regression analysis for S3 and 4 and

GT:

A) Verbal wm

Fixed effects:

	Estimate	Std. Error	z value	Pr(>|z|)	
(Intercept)	0.598440	0.125315	4.775	1.79e-06	***
GrammarType[S.Embedded]	-0.001939	0.046569	-0.042	0.9668	
Session[S.3]	-0.037214	0.048688	-0.764	0.4447	
scale(WorkingMemory)	0.272992	0.119538	2.284	0.0224	*
GrammarType[S.Embedded]:Session[S.3]	-0.116567	0.046316	-2.517	0.0118	*
GrammarType[S.Embedded]:scale(WorkingMemory)	-0.053296	0.046602	-1.144	0.2528	
Session[S.3]:scale(WorkingMemory)	0.009352	0.049541	0.189	0.8503	
GrammarType[S.Embedded]:Session[S.3]:scale(WorkingMemory)	-0.023995	0.046417	-0.517	0.6052	

Signif. codes: 0 ‘***’ 0.001 ‘**’ 0.01 ‘*’ 0.05 ‘.’ 0.1 ‘ ’ 1

$R2 = 0.024$

B) Age

Fixed effects:

	Estimate	Std. Error	z value	Pr(>|z|)	
(Intercept)	0.621946	0.124793	4.984	6.23e-07	***
GrammarType[S.Embedded]	0.038832	0.044602	0.871	0.3840	
Session[S.3]	-0.029558	0.046599	-0.634	0.5259	
scale_Age	-0.267269	0.126885	-2.106	0.0352	*
GrammarType[S.Embedded]:Session[S.3]	-0.123284	0.044407	-2.776	0.0055	**
GrammarType[S.Embedded]:scale_Age	-0.001514	0.041874	-0.036	0.9712	
Session[S.3]:scale_Age	-0.036740	0.044095	-0.833	0.4047	
GrammarType[S.Embedded]:Session[S.3]:scale_Age	-0.035652	0.041826	-0.852	0.3940	

Signif. codes: 0 ‘***’ 0.001 ‘**’ 0.01 ‘*’ 0.05 ‘.’ 0.1 ‘ ’ 1

$R2 = 0.018$

C) Corsi f.

Fixed effects:

	Estimate	Std. Error	z value	Pr(>|z|)	
(Intercept)	0.60118	0.12506	4.807	1.53e-06	***
GrammarType[S.Embedded]	0.03894	0.04422	0.881	0.37852	
Session[S.3]	-0.02754	0.04630	-0.595	0.55194	
scale(Corsi_v_Spanne)	0.26775	0.13221	2.025	0.04284	*
GrammarType[S.Embedded]:Session[S.3]	-0.11546	0.04409	-2.619	0.00883	**
GrammarType[S.Embedded]:scale(Corsi_v_Spanne)	0.13144	0.04111	3.198	0.00139	**
Session[S.3]:scale(Corsi_v_Spanne)	-0.03068	0.04257	-0.721	0.47113	
GrammarType[S.Embedded]:Session[S.3]:scale(Corsi_v_Spanne)	0.01702	0.04085	0.417	0.67684	

Signif. codes: 0 ‘***’ 0.001 ‘**’ 0.01 ‘*’ 0.05 ‘.’ 0.1 ‘ ’ 1

$R2 = 0.038$

D) Corsi bw

```
Fixed effects:
                                                   Estimate Std. Error z value Pr(>|z|)    
(Intercept)                                         0.59587    0.12778   4.663 3.11e-06 ***
GrammarType[S.Embedded]                             0.03573    0.04418   0.809  0.41871    
Session[S.3]                                       -0.03156    0.04634  -0.681  0.49589    
scale(Corsi_r_Spanne)                               0.22209    0.13532   1.641  0.10076    
GrammarType[S.Embedded]:Session[S.3]               -0.12471    0.04404  -2.832  0.00463 ** 
GrammarType[S.Embedded]:scale(Corsi_r_Spanne)       0.10747    0.04132   2.601  0.00930 ** 
Session[S.3]:scale(Corsi_r_Spanne)                 -0.02228    0.04196  -0.531  0.59537    
GrammarType[S.Embedded]:Session[S.3]:scale(Corsi_r_Spanne)  0.02182    0.04099   0.532  0.59454    
---
Signif. codes:  0 ‘***’ 0.001 ‘**’ 0.01 ‘*’ 0.05 ‘.’ 0.1 ‘ ’ 1
```

$R2 = 0.030$

E) Gender

```
Fixed effects:
                                            Estimate Std. Error z value Pr(>|z|)    
(Intercept)                                  0.56221    0.14382   3.909 9.26e-05 ***
GrammarType[S.Embedded]                      0.08150    0.04947   1.647  0.09948 .  
Session[S.3]                                -0.01269    0.05229  -0.243  0.80822    
Gender[S.F]                                 -0.07804    0.14378  -0.543  0.58726    
GrammarType[S.Embedded]:Session[S.3]        -0.13218    0.04922  -2.686  0.00724 ** 
GrammarType[S.Embedded]:Gender[S.F]          0.10745    0.04947   2.172  0.02986 *  
Session[S.3]:Gender[S.F]                     0.02819    0.05226   0.539  0.58956    
GrammarType[S.Embedded]:Session[S.3]:Gender[S.F] -0.02000    0.04922  -0.406  0.68451    
---
Signif. codes:  0 ‘***’ 0.001 ‘**’ 0.01 ‘*’ 0.05 ‘.’ 0.1 ‘ ’ 1
```

$R2 = 0.009$

F) Lesion size

```
Fixed effects:
                                                     Estimate Std. Error z value Pr(>|z|)
(Intercept)                                           0.60753    0.12911   4.705 2.53e-06
GrammarType[S.Embedded]                               0.04013    0.04442   0.903   0.3663
Session[S.3]                                         -0.04004    0.04651  -0.861   0.3894
scale(LessionSize)                                   -0.13220    0.13994  -0.945   0.3448
GrammarType[S.Embedded]:Session[S.3]                 -0.13186    0.04427  -2.979   0.0029
GrammarType[S.Embedded]:scale(LessionSize)           -0.03822    0.04163  -0.918   0.3586
Session[S.3]:scale(LessionSize)                       0.07406    0.04229   1.751   0.0799
GrammarType[S.Embedded]:Session[S.3]:scale(LessionSize)  0.01040    0.04152   0.251   0.8022
---
Signif. codes:  0 ‘***’ 0.001 ‘**’ 0.01 ‘*’ 0.05 ‘.’ 0.1 ‘ ’ 1
```

$R2 = 0.015$

G) NIHSS Difference

```
Fixed effects:
                                           Estimate Std. Error z value Pr(>|z|)    
(Intercept)                                0.612101   0.161020   3.801 0.000144 ***
GrammarType[S.Embedded]                    0.087121   0.052735   1.652 0.098526 .  
Session[S.3]                              -0.082965   0.054688  -1.517 0.129255    
NIH_dif                                   -0.003383   0.048869  -0.069 0.944802    
GrammarType[S.Embedded]:Session[S.3]      -0.142159   0.052505  -2.708 0.006779 ** 
GrammarType[S.Embedded]:NIH_dif           -0.027985   0.015270  -1.833 0.066850 .  
Session[S.3]:NIH_dif                       0.026391   0.015273   1.728 0.083987 .  
GrammarType[S.Embedded]:Session[S.3]:NIH_dif  0.008596   0.015049   0.571 0.567887    
---
Signif. codes:  0 ‘***’ 0.001 ‘**’ 0.01 ‘*’ 0.05 ‘.’ 0.1 ‘ ’ 1
```

$R2 = 0.009$

H) Aphasia in T1

```
Fixed effects:
                                              Estimate Std. Error z value Pr(>|z|)    
(Intercept)                                   0.852539   0.206275   4.133 3.58e-05 ***
GrammarType[S.Embedded]                       0.046787   0.055584   0.842   0.3999    
Session[S.3]                                 -0.017871   0.055530  -0.322   0.7476    
AphasieT1                                    -0.415754   0.382431  -1.087   0.2770    
GrammarType[S.Embedded]:Session[S.3]         -0.127440   0.055771  -2.285   0.0223 *  
GrammarType[S.Embedded]:AphasieT1            -0.086824   0.105224  -0.825   0.4093    
Session[S.3]:AphasieT1                       -0.006741   0.103182  -0.065   0.9479    
GrammarType[S.Embedded]:Session[S.3]:AphasieT1 -0.015765   0.103539  -0.152   0.8790    
---
Signif. codes:  0 ‘***’ 0.001 ‘**’ 0.01 ‘*’ 0.05 ‘.’ 0.1 ‘ ’ 1
```

$R2 = 0.012$

I) ORCP

```
Fixed effects:
                                                 Estimate Std. Error z value Pr(>|z|)  
(Intercept)                                      -0.14617    0.44698  -0.327   0.7437  
GrammarType[S.Embedded]                           0.02982    0.12177   0.245   0.8065  
Session[S.3]                                      0.04657    0.11942   0.390   0.6966  
ProdAccuracy                                      1.09156    0.54568   2.000   0.0455 *
GrammarType[S.Embedded]:Session[S.3]             -0.12285    0.11952  -1.028   0.3040  
GrammarType[S.Embedded]:ProdAccuracy             -0.08073    0.15310  -0.527   0.5980  
Session[S.3]:ProdAccuracy                        -0.08640    0.15130  -0.571   0.5680  
GrammarType[S.Embedded]:Session[S.3]:ProdAccuracy -0.02852    0.15132  -0.188   0.8505  
---
Signif. codes:  0 ‘***’ 0.001 ‘**’ 0.01 ‘*’ 0.05 ‘.’ 0.1 ‘ ’ 1
```

$R2 = 0.038$

Grammaticality:

ORCP

```
Fixed effects:
                                          Estimate Std. Error z value Pr(>|z|)
(Intercept)                               -0.15342    0.45117  -0.340 0.733811
Grammatical[S.no]                         -0.01457    0.11916  -0.122 0.902709
Session[S.3]                               0.05940    0.11922   0.498 0.618298
ProdAccuracy                               1.12922    0.55119   2.049 0.040491 *
Grammatical[S.no]:Session[S.3]             0.03346    0.11894   0.281 0.778473
Grammatical[S.no]:ProdAccuracy            -0.59167    0.15270  -3.875 0.000107 ***
Session[S.3]:ProdAccuracy                 -0.06503    0.15254  -0.426 0.669874
Grammatical[S.no]:Session[S.3]:ProdAccuracy -0.13417    0.15178  -0.884 0.376723
---
Signif. codes:  0 ‘***’ 0.001 ‘**’ 0.01 ‘*’ 0.05 ‘.’ 0.1 ‘ ’ 1
```
